# Problematic pornography use and novel patterns of escalating use: a cross-sectional network analysis with two Independent samples

**DOI:** 10.1016/j.addbeh.2024.108048

**Published:** 2024-05-02

**Authors:** Campbell Ince, Lucy Albertella, Chang Liu, Jeggan Tiego, Leonardo F. Fontenelle, Samuel R. Chamberlain, Murat Yücel, Kristian Rotaru

**Affiliations:** 1 School of Psychological Sciences, Monash University; 2 Brain Park, Turner Institute for Brain and Mental Health, Monash University; 3 Monash Biomedical Imaging, Monash University, 770 Blackburn Rd, Clayton, VIC 3800, Australia; 4 Institute of Psychiatry, Federal University of Rio de Janeiro; 5 D'Or Institute for Research and Education (IDOR); 6 Department of Psychiatry, Faculty of MedicinYasser Khazaale, University of Southampton, UK; 7 Southern Health NHS Foundation Trust, Southampton, UK; 8 QIMR Berghofer Medical Research Institute, Herston, QLD, Australia; 9 Monash Business School, Monash University

**Keywords:** Problematic pornography use, pornography addiction, behavioral addiction, binge behaviors, sexual novelty, network analysis

## Abstract

Modern internet pornography allows users to harness sexual novelty in numerous ways, which can be used to overcome desensitisation through increasing volume of use (quantitative tolerance), progressing to more stimulating genres (qualitative escalation), skipping between stimuli (tab-jumping), delaying orgasm (‘edging’), and engaging in pornographic binges. However, existing research has not yet evaluated how these potentially reciprocal consumption patterns relate to problematic pornography use (PPU). To this end, we recruited two independent samples of male pornography users (*N*_1_= 1,356, *M*_*age*_=36.86, *SD* =11.26; *N*_2_= 944, *M*_*age*_= 38.69, *SD* = 12.26) and examined the relationships between these behavioural dimensions and self-reported difficulties in controlling one’s pornography use. Data were analysed through the network analysis approach (using Gaussian graphical models). As hypothesised, i) quantitative tolerance was centrally placed within the overall network, and ii) acted as a statistical bridge node between other patterns of pornography use (e.g., pornographic binges), and all measured facets of PPU. Our results are consistent with other emerging literature suggesting that tolerance, pornographic binges, tab-jumping, and edging behaviours as relevant features of PPU, and that upscaling overall usage may connect broader patterns of use with problematic engagement. Clinical and theoretical implications, as well as future research directions, are discussed.

## 1 Introduction

Modern technology has transformed how we work, study, socialise, and engage with entertainment. With increased screen time across the globe following rapid technological advances (Bucksch et al., 2016; Smith et al., 2020), scientific and public discourse regarding potentially addictive digital behaviours continues to accelerate (Flayelle et al., 2023; Müller et al., 2022). One such behaviour of interest is the use of Internet pornography. According to a US nationally representative sample, 11% of males and 3% of females self-report feeling addicted to pornography (Grubbs et al., 2019), while one-in-seven male pornography users from a community sample expressed an interest in obtaining professional help due to the behaviour (Kraus et al., 2016). Research increasingly frames such issues as ‘problematic pornography use (PPU)’, typically defined as difficulties controlling one’s pornography consumption despite recognised adverse consequences, particularly when faced with strong urges or heightened emotional states (Bőthe et al., 2018; de Alarcón et al., 2019).

The Diagnostic and Statistical Manual fifth Edition (DSM-5-TR) (American Psychiatric Association, 2022) lists a series of potential “official”, yet very broad diagnostic labels that could, theoretically, be applied for some people with PPU, such as ‘unspecified sexual dysfunction’ (302.70/F52.8; Krueger, 2016), ‘unspecified obsessive-compulsive and related disorder’ (300.3), or ‘unspecified disruptive, impulse-control, and conduct disorder’ (312.9; Stark & Klucken, 2017). Following much debate (Gullo et al., 2022; Sassover & Weinstein, 2022), PPU can now be clinically recognised as an impulse control disorder in the International Classification of Diseases, eleventh edition (ICD-11) under the more specific entity compulsive sexual behaviour disorder (CSBD; Kraus et al., 2018). Some experts also argue that PPU aligns with a behavioural addiction model and could therefore be formally recognised in the ICD-11 as an ‘other specified disorders due to addictive behaviours’ (Brand et al., 2020), although data specific to this classification are currently limited.

Others contend that PPU is a specific Internet use disorder sitting at the intersection between compulsive sexual behaviour and Internet addiction (Brand et al., 2021; Griffiths, 2012; Love et al., 2015; Riemersma & Sytsma, 2013; Toates, 2022). This approach draws attention toward Internet pornography’s design (structural) features, especially the virtually limitless access to sexual novelty that strongly appeals to the human reproductive drive (Hilton, 2013; Love et al., 2015). This allows users to easily diversify their consumption patterns in ways that were less likely or practical in the pre-broadband era, such as upscaling their quantity of use (quantitative escalation) and/or progressing to more diverse or extreme content (qualitative escalation; Bőthe et al., 2018; Lewczuk et al., 2022). As per debates in the broader field of behavioural addiction, whether such patterns of escalation reflect addictive mechanisms remains contentious.

Scholars have acknowledged or refuted the legitimacy of sexual tolerance to varying degrees. Recent reviews generally point to equivocal evidence (or lack of direct examination for tolerance) when focusing on higher-order constructs like sexual addiction or compulsive sexual behaviour (Pistre et al., 2007; Sassover & Weinstein, 2022; Wines, 2007). In contrast, pornography-related tolerance has attracted greater empirical support in recent years (although such work certainly remains in its infancy; Bothe et al., 2020; Chen & Jiang, 2020; Ince et al., 2021, 2023; Palazzolo & Bettman, 2020; Kuhn & Gallinat, 2014; Pistre et al., 2007; Wery & Billieux, 2016). Specifically, emerging self-report (Bőthe et al., 2018; Chen et al., 2021; Ince et al., 2021; Lewczuk et al., 2022) and neuroimaging data (Kühn & Gallinat, 2014; Voon et al., 2014) relying primarily on male samples supports the involvement of tolerance-related processes in PPU. A growing body of qualitative observations also suggest that users can engage with additional mutually reinforcing behaviours to escalate or intensify their pornography use (hereafter, ‘intensity indicators’; Ince etf al., 2023), including pornographic binges marked by hours-long sessions and/or multiple orgasms, frequently shifting between stimuli (‘tab-jumping’), or deliberately prolonging climax to extend a session (‘edging‘; Blinka et al., 2022; Fernandez et al., 2021; Hanseder & Dantas, 2023; Ince et al., 2023; Palazzolo & Bettman, 2020). Even if not considered ‘core’ features of PPU, such dimensions (pornographic binges, tab-jumping, and edging) hold clinical significance and may further our understanding of PPU relative to similar constructs like CSBD and problematic usage of the Internet (Gola et al., 2020). Disentangling the presumably nuanced relationships between these dimensions of pornography use and individual facets of PPU would further our understanding of the facets that drive and maintain this problematic behaviour (Brand et al., 2019; Gola & Potenza, 2018).

Psychological network analysis is a potentially valuable tool to examine the relationships between PPU and various dimensions of pornography use. Traditional approaches examine relationships between latent variables (as in structural equation modelling) or sum scores (as in path analysis, regression models; Kline, 2012). The theoretical assumption of these models is twofold: first, the psychological syndrome of interest is unidimensional; and second, there exists an underlying ‘common cause’ that explains the association between symptoms. (Fried & Nesse, 2015; Kline, 2012). These theoretical assumptions are both conceptually and empirically inconsistent with qualitative and quantitative investigations of PPU. Such work has shown PPU to be a multifaceted problem, where relevant features interact in complex ways (Ince et al., 2023; Chen et al., 2019; Bothe et al., 2020). In contrast, the network perspective postulates that mental health conditions are driven and maintained by dynamic interactions between indicators (e.g., survey items; Boorsbom et al., 2021). This permits researchers to distil a complex multivariate space into pairwise connections ('edges') among variables ('nodes'), which can identify granular relationships that would otherwise be obscured with a sum score or latent variable approach (Borsboom et al., 2021; Epskamp et al., 2018). This is especially relevant in the current context given the unexamined relationships among intensified usage patterns (e.g., qualitative escalation, pornographic binges) and established facets of PPU (Blinka et al., 2022; Hanseder et al., 2023; Ince et al., 2023; Palazzolo & Bettman, 2020).

Statistically, the network approach gives rise to two key indices: centrality estimates and bridge nodes. Centrality estimates, such as node strength or expected influence, measure how strongly a variable is connected throughout the network as a function of the number and size of observed connections (i.e., an index for a node’s overall information; Contreras et al., 2019). Bridge nodes offer complementary information but focus specifically on relationships across discrete constructs (‘communities’ of items) within the network. For instance, certain aspects of depression (fatigue, concentration problems) have been identified as bridge nodes that connect particularly strongly to specific indicators of problematic smartphone use (self-control failure, recklessly continuing smartphone use; Wei et al., 2023). Applied to the current study, bridge nodes may identify which under-researched intensity indicators may act as intermediary variables that link other escalating behaviours with measured dimensions of PPU.

The network approach has been increasingly applied to addictive behaviour research, including problematic smartphone use (Guo et al., 2023; Liu et al., 2022, 2023), social networking (Li et al., 2023; Svicher et al., 2021), and hypersexuality (Marchetti, 2023; Werner et al., 2018; Zarate et al., 2022), and has recently been extended to PPU (Bőthe et al., 2020; Chen et al., 2021; Jiang, Lu, et al., 2022a, 2022b). Although such work suggests that tolerance is a phenomenon relevant to PPU, other forms of escalating use (intensity indicators) remain largely unexamined in the quantitative literature. Some argue that the interplay between various escalating behaviours helps to explain how certain users may develop compulsive or potentially addictive engagement with pornography (de Alarcón et al., 2019; Mead & Sharpe, 2020; Meerkerk et al., 2006). A network analysis could further such knowledge by identifying the relationships among various escalating behaviours (intensity indicators) and subsequent links to specific facets of PPU.

As such, we applied psychological network analysis to examine the links between various intensity indicators (qualitative and quantitative tolerance, binges, edging, and tab-jumping) and established facets of PPU among two independent samples of males who use Internet pornography. We limited our sample to males given that the exploratory research informing our investigation relied almost exclusively on male respondents (Blinka et al., 2022; Fernandez et al., 2021; Hanseder et al., 2023; Ince et al., 2023; Palazzolo & Bettman, 2020), while other work suggests that PPU is substantially more common among men and is driven by different mechanisms compared with women (e.g., novelty seeking; Kafka, 2010; Kor et al., 2014).

We hypothesised that quantitative tolerance, indicative of escalating time with pornography, would be especially central to the overall network given previous links between increasing use and compromised control (e.g., diminished control over urges). Based on accumulating qualitative research (Blinka et al., 2022; Hanseder et al., 2023; Ince et al., 2023; Palazzolo & Bettman, 2020), we also predicted that quantitative tolerance would act as a bridge node connecting other intensity indicators – such as genre escalation, tab-jumping, edging, and frequency of binges – to well recognised facets of PPU (e.g., difficulty resisting urges to use pornography).

## 2 Methods

### 2.1 Participants and recruitment

We recruited male pornography users (defined as having consumed Internet pornography at least once in the past twelve months) through two large online crowdsourcing platforms (Sample 1: CloudResearch’s *Connect* platform (USA only); Sam*ple 2: Prolific* platform (UK only)). The survey was advertised as “a study on male sexual behaviours (including pornography use) and how these relate to sexual and psychological well-being”, with no negative connotations toward PPU or similar problems. We provided the following widely used definition of pornography: “*Pornography is defined here as material that creates or elicits sexual feelings or thoughts and contains explicit exposure or descriptions of sexual acts involving the genitals (e.g*., *vaginal or anal intercourse, oral sex, or masturbation)*” (Bőthe et al., 2018; Reid et al., 2011). All respondents were reimbursed within the recommended ranges for each recruitment platform. As mentioned above, we restricted the sample to males given recognised gender differences in rates and mechanisms associated with PPU (Kafka, 2010; Kor et al., 2014). The study protocol was approved by the Monash University Human Research Ethics Committee (project #37969) and all respondents provided informed consent. The study was conducted in accordance with the Declaration of Helsinki.

### 2.2 Measures

Respondents provided sociodemographic information on age, education, relationship status, and religiosity. Regarding natural history of sexual behaviours, participants indicated their number of sexual partners (lifetime and past six months) and their frequency of pornography use, masturbation without pornography, and partnered sex (each measured as 1= *Never*, 9= *Multiple times per day*).

PPU was measured using the five-item Brief Pornography Screener (BPS; Kraus et al., 2020), which is the gold-standard brief screener for PPU measuring key facets like difficulty reducing pornography use, difficulty resisting strong urges, and using pornography to cope with strong emotions in the past six months. Items are rated on a three-point Likert scale (0= *Never* , 1= *Occasionally*, 2= *Very often*), with scores ≥4 indicating possible PPU (Chen et al., 2021; Kraus et al., 2020). Internal consistency reliability in both samples was considered good (McDonald’s omega = 0.89 [95%*CI* = 0.88-0.90] for both Samples 1 and 2).

Given that the BPS does not contain items related to tolerance/escalation, we also administered the *Tolerance* subscale of the Problematic Pornography Consumption Scale (Bőthe et al., 2018). This included two items for quantitative escalation (“*I felt that I had to watch more and more pornography for satisfaction” and “I felt that I needed more and more pornography in order to satisfy my needs*”) and one for qualitative escalation (“*I gradually watched more “extreme” pornography, because the porn I watched before was less satisfying*”). These three items were measured on a seven-point Likert scale (1= *Never*, 7= *All the time or almost all the time*). For our analyses, we averaged the two items for quantitative tolerance to create a single node for this dimension, and included this alongside the single item for qualitative tolerance.

Based on Wordecha et al.’s (2018) observational findings regarding patterns of binge pornography among individuals seeking treatment for CSBD, we defined pornographic binges as a sitting of at least two hours long and/or involving multiple orgasms. This definition was provided to respondents, who first indicated whether they had ever binged with pornography (binary yes/no). Those who responded affirmatively were then asked “In the past six months, how often (on average) have you binged with pornography?” (1= *No binges in the past six months*, 9= *Multiple times per day*). Those without a history of pornographic binges were re-coded as “0”. Both samples had very low endorsement for scale points 7-9 (*“4-6 times a week or more*”, “*Daily*”, and *“Multiple times per day*”). These three points were cumulatively endorsed at 1.33% and 0.53% for Samples 1 and 2, respectively, and were therefore re-coded into the next highest scale point (6; “*2-3 times per week”*).

Edging behaviours (“*When you use pornography, how often do you delay climax in order to prolong the session?*”) and tab-jumping tendencies (“*When using pornography, how frequently do you change to new stimuli during one sitting (e.g. jumping between multiple tabs or thumbnails)?*”) were both measured as separate items on a five-point Likert scale (1= *Never or almost never*, 5= *Constantly*; Ince et al., 2021).

### 2.3 Statistical analysis

JASP 0.17.2 (JASP Team, 2023) was used to calculate descriptive statistics, internal consistency reliability and inferential statistics (Mann-Whitney *U*, independent samples *t*-tests, and chi-square tests of independence). Statistical significance was set at α <.05. R-Studio version 4.2.2 (R Core Team, 2021; https://www.r-project.org/; Vienna, Austria) was used for network analysis.

#### 2.3.1 Network estimation

We used the *qgraph* package to construct and visualise a Gaussian graphical model with polychoric correlations to account for skewed data (Borsboom et al., 2021; Epskamp et al., 2012). This utilised a LASSO regularisation (statistical penalty) to shrink spurious edges to zero to favour a sparse network structure (Fried et al., 2019). This penalty is controlled by a tuning parameter (lambda) determined by the Extended Bayes Information Criterion (EBIC; Foygel & Drton, 2010). We also set the EBIC hypertuning parameter (γ=0.50) at the conservative end of the conventional range (0-0.50; Hevey, 2018). The Fruchterman-Reingold algorithm (Fruchterman & Reingold, 1991) was used to visualise the network. This algorithm places the most statistically prominent variables (nodes) most centrally in the layout, positions related nodes proximally to one another, and uses thicker lines to indicate stronger associations (edges). Node predictability was also calculated to quantify how well any given node is statistically predicted by neighbouring nodes, interpreted akin to *R*^*2*^ (Haslbeck & Waldorp, 2018).

#### 2.3.2 Network inference

We used the *qgraph* package (Epskamp et al., 2012) to calculate node expected influence (EI), which represents the absolute sum of all edge weights connected to a node. Recent work shows that node EI is more appropriate than traditional centrality indices (strength, closeness, and betweenness) for psychological networks, especially when the network contains negative edges (Bringmann et al., 2019; Robinaugh et al., 2016). Noting that EI upwardly biases strongly correlated items (in this case, the five items from the unidimensional BPS), we re-estimated the model using a BPS composite score. This permitted a more granular analysis of node centrality that may be obscured by including the BPS items as individual nodes. We therefore considered two models: one with BPS items as individual nodes (Model 1), and the other using a composite score for the BPS (Model 2). We used EI to index node centrality in both Models 1 and 2 as it can handle models with and without negative edges (Robinaugh et al., 2016).

Given our focus on the relationships between different patterns of pornography use and established features of PPU, the networks contained two predefined communities: the first representing PPU (measured by the BPS) and the second representing pornography use intensity indicators (quantitative tolerance, qualitative tolerance,, binges, edging, and tab-jumping). We used the *networktools* package (Jones, 2017) to then examine which variables act as ‘bridge nodes’ that statistically connect the two communities. This was quantified as bridge expected influence (BEI), which represents the absolute sum of all edge weights that connect a node to its neighbouring community. As such, a higher BEI value suggests that a node may exert a more diffuse influence across the network (Jones et al, 2021). BEI was only calculated for Model 1 given that the PPU community in Model 2 was conceptually redundant as it contained only a single node (BPS composite score).

#### 2.3.3 Network accuracy and stability

We examined network robustness using the *bootnet* package (Epskamp et al., 2018). We evaluated edge weight accuracy with non-parametric bootstrapping (2,500 bootstrap samples), whereby narrower 95% confidence intervals indicate greater accuracy (Epskamp et al., 2018). We also used the bootstrap difference test to examine differences between two bootstrapped edge weights and centrality estimates.

EI and BEI stability were examined with the correlation stability (CS) coefficient generated with 2,500 case-dropping bootstrap samples. The CS coefficient represents the greatest number of cases that can be dropped to maintain a 0.70 correlation (at 95% probability) between the estimated centrality estimates and a subset of estimated networks (Epskamp et al., 2018). Following expert guidelines, the CS coefficient should exceed 0.50 and at a minimum 0.25 (Epskamp et al., 2018).

#### 2.3.4 Sensitivity analyses

We conducted numerous sensitivity analyses to ensure stability of the results. Firstly, the network comparison test (*networkcomparisontest* package Van Borkulo et al., 2022) was used to compare the network characteristics (edges, nodes, as well as global and local strength; 2,500 iterations) across the two samples.

Secondly, consistent with previous work using three-point ordinal data (Scarth et al., 2023; Chattratrai et al., 2022; Baggio et al., 2022), we re-estimated Model 1 as a mixed graphical model (MGM) with all three-point items (i.e. all BPS items) modelled as categorical and all remaining items as continuous. Model 2 was not re-estimated as an MGM given that the three-point BPS items were summed into a composite score. As described in the Supplementary materials, these MGMs were estimated with the “AND” regression rule and the EBIC hypertuning parameter was set to the conventional γ=0.25 (Haslbeck & Waldorp, 2018). Moreover, the MGMs were estimated with and without paranormal transformation to account for skewed data (Liu et al., 2022).

Thirdly, we re-estimated the networks after removing the BPS items related to increasing time spent with pornography (BPS_1: “*You find yourself using pornography more than you want to*”; BPS_2: “*You have attempted to “cut back” or stop using pornography but were unsuccessful*.”). This was due to the possibility that such items share close relationships with various nodes from the ‘intensity indicators’ community (especially edging, binge frequency, and tab-jumping). After removing these items, the BPS network contained items pertaining only to other key facets of PPU, namely difficulties resisting strong urges (BPS_3), using pornography to cope with strong emotions (BPS_4), and continuing to use pornography despite feeling guilty (BPS_5).

Fourthly, recognising that PPU severity follows a zero-inflated (quasi-normal) distribution (Tiego et al., 2019) and that related dimensions may interact differently across different levels of severity, we re-estimated the networks in two ways: i) removing individuals with a BPS score of zero and comparing the results to the original samples, and ii) comparing the networks for individuals below vs. above the established cut-off score (BPS≥4).

## 3 Results

The initial samples consisted of *N*_*1*_= 1,521 and *N*_*2*_= 976. After *N*= 197 (aggregated across samples) were removed due to quality checks (Supplementary File S1, Appendix A), the final samples consisted of *N*_*1*_=1,356 and *N*_*2*_=944 respondents. Information on sociodemographics and natural history of sexual behaviours is presented in Table 1. Abbreviations, mean score and standard deviations for variables included in the networks are presented in Table 2.

### 3.1 Network structure

**Figure 1:**
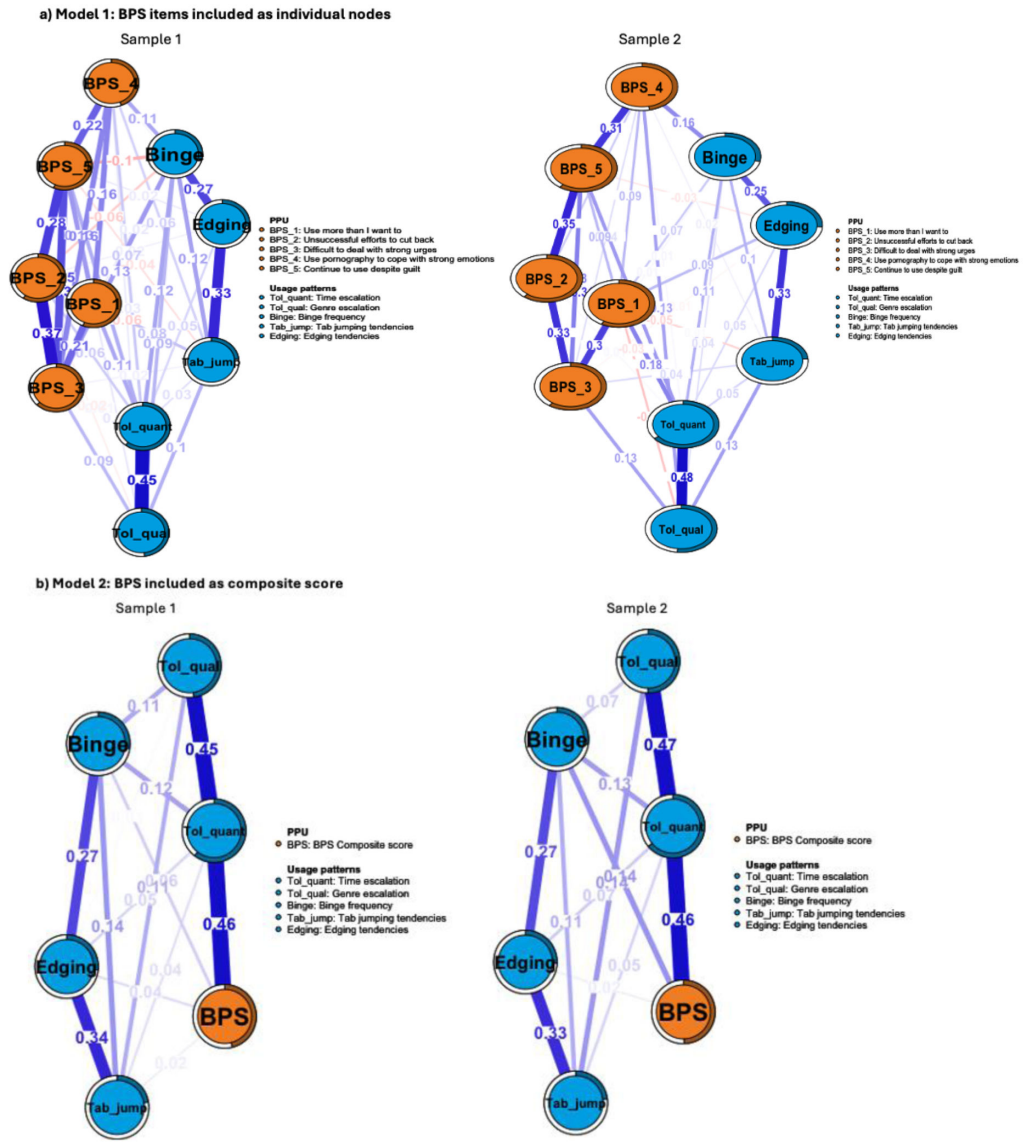
Estimated networks for Samples 1 and 2.

Key characteristics for the estimated networks (number of edges retained, mean edge weight, and cross-community edges) are presented in Table 3. The visualised networks are presented in Figure 1.

For Model 1, the strongest edges were observed within the BPS community, ranging between 0.00-0.37 (Sample 1) and 0.04-0.35 (Sample 2). Noteworthy edges within the ‘intensity indicators’ (usage patterns) network were between the qualitative and quantitative tolerance (Sample 1 edge weight: 0.39; Sample 2 edge weight: 0.41), edging and tab-jumping (edge weight: 0.33 in both samples), edging and binge frequency (Sample 1 edge weight: 0.26; Sample 2 edge weight: 0.25), and tolerance and binge frequency (both samples). As shown in Supplementary Figure B1, edge bootstrapped 95% *CI*s were narrow, indicating accurate and reliable edge weights. Supplementary Figure B2 shows the bootstrapped differences for all edge weights.

For Model 2, the nodes with strongest links with PPU severity (BPS composite score) were quantitative tolerance (edge weight= 0.46 in both samples) and binge frequency (edge weights for Samples 1 and 2, respectively: 0.06, 0.14). Only quantitative tolerance and binge frequency were connected with all other nodes in Model 2. Edging also shared notable connections with both tab-jumping and binge frequency (edge weights between 0.26-0.33 across samples), indicating an indirect relationship to quantitative tolerance.

### 3.2 Node centrality

#### Model 1 (BPS items modelled as individual nodes)

**Figure 2:**
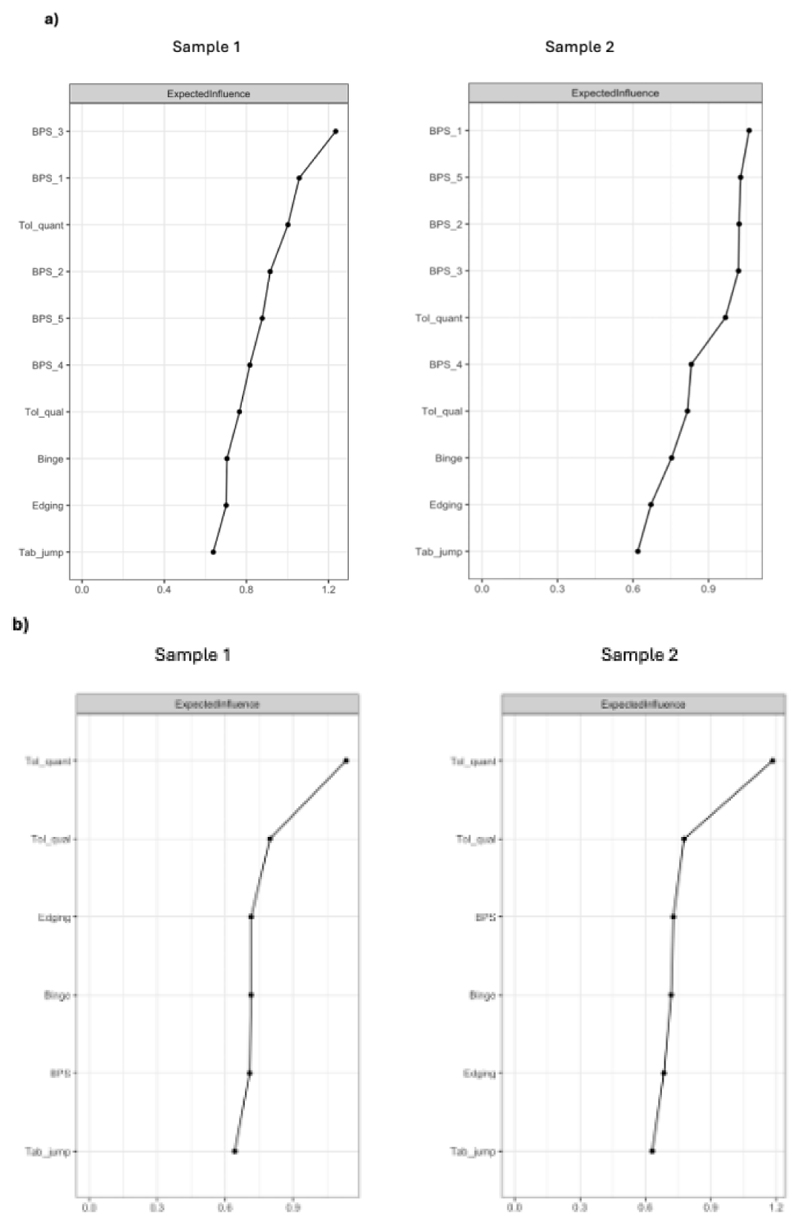
Node centrality (expected influence) estimates for across samples. Upper panel a) represents Model 1 (BPS items included as separate nodes). Lower panel b) represents Model 2 (BPS included as composite score).

For Sample 1, the most central nodes in Model 1 were BPS_1 (using pornography more than you want to) and BPS_3 (difficulty resisting strong urges), followed equally by quantitative escalation and BPS_2 (unsuccessful efforts to reduce pornography use). For sample 2, all five BPS items and quantitative escalation were equally central to the estimated network. Node centrality estimates across samples are shown in Fig. 2 Fig. 2a.

Supplementary Figure B3 shows bootstrapped difference tests for node EI. The CS coefficient for node EI exceeded 0.50 (CS_EI_= 0.75 for both samples), indicating adequate stability (Supplementary Figure B4).

#### Model 2 (BPS modelled as composite score)

Across samples, quantitative escalation was the most central node in Model 2 (Fig. 2b). Supplementary Figure B3 shows Model 2 bootstrapped difference tests for node EI. The CS coefficient for node EI exceeded 0.50 (CS_EI_= 0.75 for both samples), indicating adequate stability (Supplementary Figure B4).

### 3.3 Bridge nodes

#### Model 1 (BPS items modelled as individual nodes)

**Figure 3:**
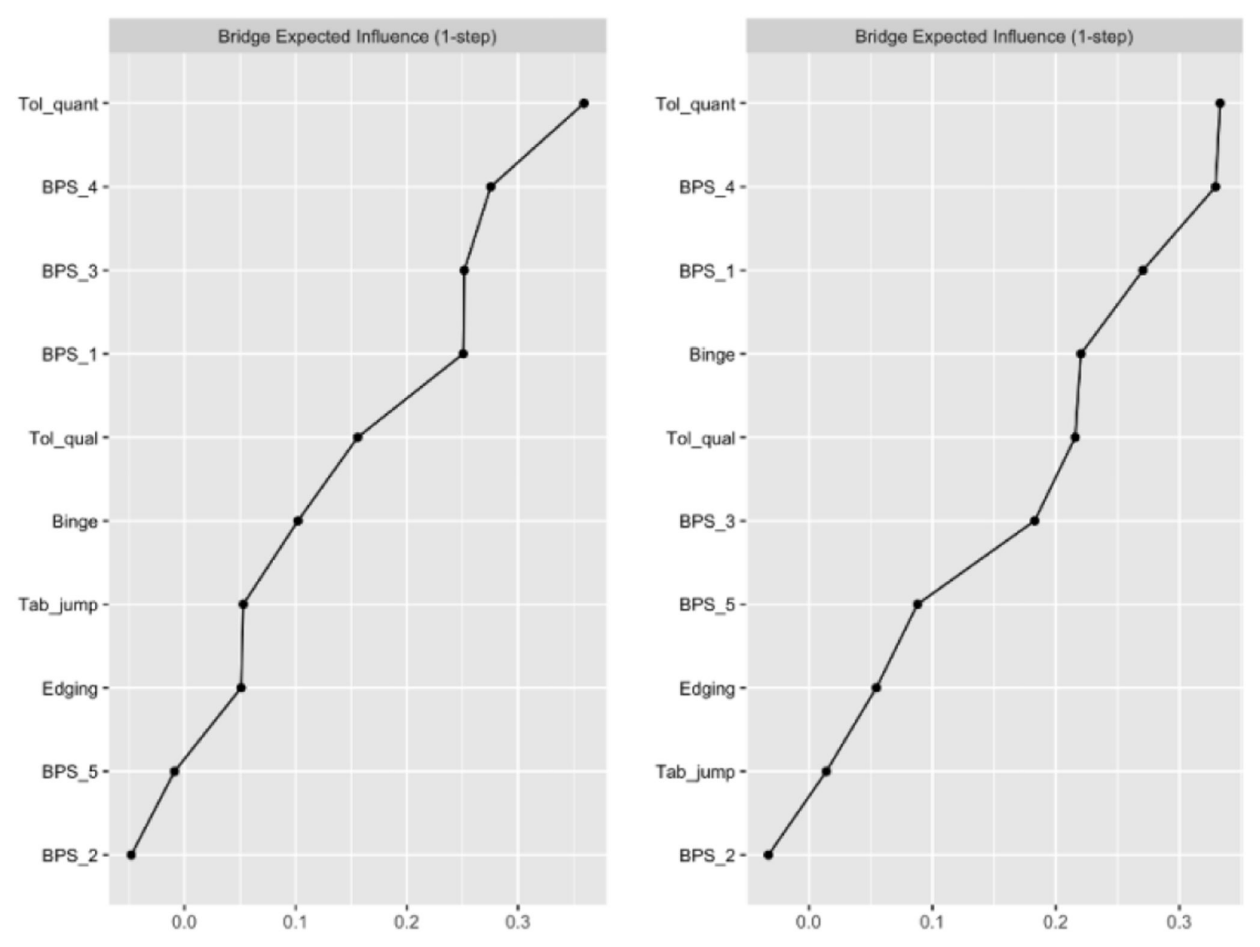
Bridge centrality (bridge expected influence) estimates across samples. *Note*. Bridge centrality estimates not applicable for Model 2 given that the PPU community consisted of a single node (BPS composite score).

For Sample 1, quantitative tolerance and three BPS items (BPS_1: using pornography more than you want to); BPS_3: difficulty resisting strong urges; BPS_4: using pornography to cope with strong emotions) were identified as the strongest bridge nodes. Similar results were observed for Sample 2, with quantitative tolerance, BPS_4 and BPS_1 identified as the strongest bridge nodes. Bridge centrality estimates are shown in Fig. 3.

Binge frequency was also a notable bridge node that was directly connected with BPS_4 (using pornography to cope with strong emotions) and BPS_3 (difficulty resisting strong urges). Supplementary Figure B5 shows bootstrapped difference tests for BEI. The CS coefficient for BEI in both samples was excellent (CS_BEI_= 0.75 in both Samples 1 and 2; Supplementary Figure B7).

### 3.4 Sensitivity analyses

Our four sensitivity analyses yielded negligible observations, indicating stability of results. Specifically, the network comparison test indicated equivalence across the two samples given no significant differences in the distribution of edge weights (Model 1: *M*= 0.08, *p*= .69; Model 2: *M*= 0.06, *p*= 0.72) nor global strength (Model 1: *S*= 0.13, *p*= .28, Model 2: *S*= .02, *p*= 0.76). As such, both network models were deemed to replicate across samples.

As shown in the Supplementary Materials (see Sensitivity Analyses 1 through 6 [SA1-SA6]), consistent results were observed after i) re-estimating the networks as mixed graphical models with and without paranormal transformations, ii) removing BPS items related to increased time (BPS_1: “*You find yourself using pornography more than you want to*.”; BPS_2: “*You have attempted to “cut back” or stop using pornography but were unsuccessful*.”), iii) re-estimating the networks after removing individuals with a BPS score of zero, and iv) comparing the networks for individuals above vs. above the established cut-off score (BPS≥4). Specifically, quantitative tolerance was consistently found to be a key node (for both node centrality and bridge centrality) across all sensitivity analyses, indicating stability of the results.

## 4 Discussion

The current study recruited two independent samples of male pornography users to identify which under-researched dimensions of intensified pornography use (intensity indicators) are most closely linked to PPU. Consistent with emerging (primarily qualitative) research, our findings confirmed our prediction that various behavioural dimensions (namely, escalating time and/or genres consumed, pornographic binges, edging, and tab-jumping) may be relevant to PPU (Blinka et al., 2022; Ince et al., 2021, 2023; Palazzolo & Bettman, 2020; Wordecha et al., 2018). Of these intensity indicators, we found that quantitative tolerance (escalating time required to reach sexual satisfaction) was most widely connected in the overall network as evidenced by node and bridge centrality estimates. Said differently, quantitative tolerance was particularly linked to not only other intensity indicators (e.g., genre escalation, binge frequency), but also established PPU dimensions (e.g., difficulty resisting urges for pornography). As per previous self-report (Bőthe et al., 2018; Chen et al., 2021; Ince et al., 2021; Lewczuk et al., 2022) and neurobiological data (Banca et al., 2016; Voon et al., 2014), this implies that escalating engagement, alongside diminished pleasure, may be an important PPU characteristic. In support of our second hypothesis, the position of quantitative tolerance as a statistical bridge node suggests an intermediary role between other clinically relevant pornography behaviours (e.g., binges) and impaired behavioural control (Ince et al., 2023; Wordecha et al., 2019). Links between tab-jumping and edging are also intriguing and support the idea that individuals with PPU may increasingly pursue sexual novelty while using pornography (Banca et al., 2016; Blinka et al., 2022; Ince et al., 2023; Love et al., 2015; Voon et al., 2014).

To the best of our knowledge, our study is the first to directly quantify pornographic binges, thereby extending qualitative observations among clinical and sub-clinical PPU samples (Blinka et al., 2022; Ince et al., 2023; Palazzolo & Bettman, 2020; Wordecha et al., 2018). This also corroborates the observations of Lewczuk et al. (2021) in which binge-like pornography behaviours (e.g, the longest ever sitting with pornography and the variability of genres consumed) were associated with PPU severity. We extend these observations by directly operationalising pornographic binges and demonstrating that binge frequency was primarily (but not exclusively) linked to PPU through tolerance, which indicates a potential mediation effect. We also found direct links between binge frequency and PPU-related dimensions, like using pornography for mood management and difficulties resisting pornographic urges. On balance, these binge-related observations are broadly consistent with the literature on substance abuse (Shmulewitz et al., 2023) and other dysregulated behaviours like binge watching (Forte et al., 2021) and binge eating (Joyner et al., 2015; Reents & Pedersen, 2021). However, we must reiterate that research into pornographic binges remains in its infancy and warrants greater attention. To echo Wordecha and colleagues (2018), improving how we define and operationalise pornographic binges is an important step to enhance precision in this area given that current definitions on what constitutes a binge is arbitrary (Flayelle & Lannoy, 2021; Lewczuk et al., 2021; Wordecha et al., 2018). Enhancing such definitions through empirical and clinical evaluation is an avenue for future work.

### 4.1 Theoretical and clinical implications

Our findings align with suggested overlaps between PPU and problematic usage of the internet (‘Internet addiction’) as digitally-mediated sexual novelty seemingly underscores each of our behavioural patterns of interest (especially tolerance, pornographic binges, and tab-jumping; Hilton, 2013; Love et al., 2015). It is intuitive that these usage dimensions may characterise PPU more than other forms of CSBD, especially offline manifestations (Brand et al., 2019; Brand & Potenza, 2023; Riemersma & Sytsma, 2013). Moreover, these features are likely less central to other cybersexual behaviours (e.g., webcamming or live sex shows), which typically involve real-time interactions with few performers, which cannot match the essentially unlimited sexual novelty available through pre-recorded Internet pornography. As such, the frequency at which viewers can rapidly move between stimuli and/or progress to new genres is likely greater in the context of PPU than other forms of cybersex and offline CSBD, although these are avenues for future research.

Our findings regarding tolerance are also notable given the ongoing controversies for tolerance in behavioural (non-substance) addictions. Such debates rightly argue that escalating behaviours do not automatically reflect an addiction model, or even functional impairment, especially if motivated by non-pathological factors like sexual curiosity/exploration, skill mastery, or social connection (Kardefelt-Winther et al., 2017; Sassover & Weinstein, 2022). However, our tolerance-related items (taken from the PPCS; Bőthe et al., 2018) focused specifically on escalation related to perceived (i.e. self-reported) desensitisation/habituation effects and were also directly linked with all measured facets of PPU, which is consistent with neurobiological data related to PPU/CSBD (Banca et al., 2016; Kühn & Gallinat, 2014; Voon et al., 2014).

Our work suggests that organisations providing support for people with PPU should ask affected individuals about a range of pornography related behaviours that might be indicative of problematic patterns of use, including tolerance (Blinka et al., 2022, 2022; Ince et al., 2023; Palazzolo & Bettman, 2020). In a practical sense, the ICD-11 offers a useful starting point through the CSBD criterion describing “little or no satisfaction from the behaviour” (Kraus et al., 2018), from which clinicians can evaluate pornography behaviours that might drive and maintain habituation, such as qualitative and quantitative escalation, pornographic binges, edging, and tab-jumping (Gola et al., 2020). Although clinicians using the DSM-5-TR are guided by less specific diagnostic criteria (see introduction, otherwise refer to Krueger, 2016 or Stark & Klucken, 2017), clinicians would be well served to make similar inquiries and exert clinical judgement about a range of pornography use behaviours. Clinicians may especially note the links between tolerance and mood management and difficulty resisting urges for pornography, which indicates that problematic pornography users self-medicate against negative affective states by upscaling their pornography use (Antons et al., 2019; Gola et al., 2017). Finally, notwithstanding our preliminary (cross-sectional) results, our findings suggest that quantitative tolerance may act as an intermediary between other forms of intensive pornography use (e.g., escalating genres, pornographic binges) and various facets of PPU (e.g., excessive use, difficulty resisting urges). Additional research investigating the role of quantitative tolerance with respect to other signs of PPU is therefore important. Findings may suggest that quantitative tolerance is warranted as a potential intervention target.

### 4.2 Strengths, limitations and future directions

A key strength of this study was our use of the psychological network approach, which revealed granular relationships between individual facets of PPU and various under-researched patterns of pornography use. Moreover, our work was the first to separately investigate the roles of quantitative (time-based) vs. qualitative (content-based) escalation, and is among the first to quantify pornographic binges, edging, and tab-jumping (Ince et al., 2021). Our findings also replicated across two independent samples, which enhances robustness of the findings and addresses replicability concerns in psychological network analysis (Borsboom et al. 2017; Hevey, 2018).

However, our study also contained numerous important limitations. First, our cross-sectional design precludes any causal implications as to the dynamics and trajectory of PPU and related features. Second, our sole use of an all male sample is a significant limitation as women are also affected by CSBD and the important extension of PPU research to incorporate both sexes has recently been discussed (de Alarcon et al., 2019; Grubbs et al., 2020; Kowalewska et al., 2023). Our sample also had limited sociocultural diversity, and our recruitment strategy may have further introduced sampling bias by appealing to those interested in surveys on sexual behaviours. Future work should consider extending our study to better encompass sexual, gender, and cultural diversity (such as through nationally representative samples), as well as among clinical populations.

Third, our variable selection may have influenced the results given that the BPS does not provide comprehensive coverage of relevant PPU dimensions. Other scales like the PPCS (beyond the *Tolerance* subscale used here) include additional dimensions like salience, withdrawal, and conflict. Although tolerance (alongside withdrawal) was reported as a central node in community and sub-clinical samples (Chen et al., 2021) and among individuals who had and had not considered treatment for PPU (Bothe et al., 2020), other work found that salience and withdrawal were more central to PPU than tolerance (Bothe et al., 2020). To this end, while our results speak to the importance of quantitative tolerance, our work should be extended to include measures that cover a broader range of PPU-related dimensions.

Fourth, we recognise that pornography behaviours related to tolerance/escalation, binges, edging, and tab-jumping are not inherently problematic *per se*. This highlights the need to identify predictive, mediating, and moderating factors between such usage patterns and PPU, which may include sexological (e.g., sexual excitation/inhibition, sexual motivations; Bőthe et al., 2021; Nolet et al., 2021), personality (e.g., neuroticism; Asaoka et al., 2020) and psychological (e.g., reward sensitivity; Klein et al., 2022) dimensions. Although the included items related to tolerance specifically inquired about escalating use to achieve or maintain satisfaction (Bothe et al., 2017), we also recognise that escalating behaviours can reflect non-pathological mechanisms such as curiosity, exploration, and skill mastery, and reiterate the importance of such distinctions in future work (Kardefelt-Winther et al., 2017; Sassover & Weinstein, 2022). Person-centred heterogeneity analyses may also help to identify users who exhibit tolerance/escalation without diminished control and to identify different combinations of escalating use.

Fifth, we recognise that network analysis is not without its critiques and limitations. Chief among these is that our cross-sectional data cannot be generalised to longitudinal dynamics between variables. It therefore remains to be seen whether quantitative tolerance may be central to the network or as a key bridge node in a longitudinal analysis (Wichers et al., 2017). Inferences about individual-level symptom dynamics are also drawn from group-level associations (Bos & Wanders, 2016). This mismatch between the level of analysis and the theoretical underpinnings of network theory (which emphasises individual-level symptom interactions) is a recognised limitation of the network approach (Neal et al., 2017). Alternative person-centred approaches (e.g., latent profile analysis) may therefore shed light on relationships among variables that differ across individuals in ways that network analysis is not equipped to do (Chen et al., 2022; Jiang et al., 2022). Additional (missing) nodes also represent a key limitation of network analysis (Neal & Neal, 2021). Notwithstanding the potential shortfalls of over-controlling for potential confounds (Wright, 2021), including other intensity indicators or PPU dimensions would have likely generated different results. Finally, critics of the network approach point to the inconsistency and instability of network parameters, including specific edges (Forbes et al., 2017). Future work using multiple indicators (rather than single items to represent nodes) may help to mitigate the unknown level of measurement error that is often raised regarding network analysis (de Ron et al., 2022). Although our multi-sample design partly addresses such concerns, the relationships may not generalise to other community samples (i.e., non-crowdsourcing), sub-clinical, or treatment seeking samples.

### 4.3 Conclusion

Our work is the first to quantify the links between various dimensions of intensified pornography use (pornographic binges, edging, tab-jumping, and escalations in usage and genres) and recognised facets of PPU. Our cross-sectional results suggest that quantitative tolerance (increasing time spent with pornography to satisfy one’s needs) is particularly related not only to PPU but also to other indices of intensified use. Extending these observations longitudinally will help to establish the dynamics among these usage dimensions and PPU. In the meantime, researchers and clinicians should consider various forms of escalating use when examining PPU, each of which may be driven by maladaptively pursuing sexual novelty. This information would build on our findings and further our understanding of PPU relative to other dysregulated and problematic sexual behaviours.

#### Declaration of Generative AI and AI-assisted technologies in the writing process

During the preparation of this work the first author used ChatGPT to proofread and enhance clarity of the manuscript. After using this tool/service, the authors reviewed and edited the content as needed and take full responsibility for the content of the publication.

## Supplementary Material

Supplementary Materials

## Figures and Tables

**Table 1:** Descriptive statistics and group comparisons for sociodemographics and natural history of sexual behaviours across samples.

Characteristics	Sample 1 (USA; *N*= 1,356) *n *(%) / *M *(SD)	Sample 2 (UK; *N*= 944) *n *(%) / *M *(SD)	Chi square tests of independence / Independent samples *t*-tests
***Sociodemographics***			
Age, years	36.86 (11.26)	38.69 (12.26)	*t*(1916)^a^= -3.63, *p*<.001, *d*= -0.16
Education			
None	<1%	<1%	χ^2^(4, *N*=2,300)= 14.92, *p*<.001, *V*= 0.08
Primary/elementary school	1%	<1%	
Secondary school	31%	25%	
Tertiary	51%	55%	
Higher education	17%	20%	
Relationship status			
Single	40%	29%	χ^2^(4, *N*=2,300)= 67.55, *p*<.001, *V*= 0.17
In a relationship	21%	35%	
Married	34%	32%	
Divorced/separated	4%	2%	
Widowed	<1%	<1%	
Religious denomination^b^			
Agnostic/atheist	47	61	χ^2^(6, *N*=2,300)= 7.21, *p*=0.21, *V*= 0.19
Buddhist	2	1	
Christian/catholic	36	24	
Jewish	2	0	
Muslim	2	3	
Other	8	5	
Prefer not to disclose	4	5	
Religiosity	1.31 (1.47)	0.76 (1.17)	*t*(2,258)^a^= 9.93, *p*<.001, *d*= 0.41
***Natural history of sexual behaviours***			
Pornography use frequency	5.19 (1.84)	5.13 (1.90)	*t*(2,298)= 0.76, *p*=.45 *d*= 0.04
Porn-free masturbation frequency	3.00 (2.26)	3.23 (2.42)	*t*(2,298)= -2.29, *p*=.0.03, *d*= -0.10
Partnered sex frequency^c^	3.82 (2.35)	3.89 (2.40)	*t*(2,118)= -0.75 *p*= 45, *d*= -0.03

*Note*.

a Welch-corrected due to unequal variances.

B Total exceeds 100% due to individuals with multiple religious denominations (*n*=18).

c Only includes individuals with any history of partnered sex (*N*_*1*_=1,219, *N*_*2*_ = 901). Religiosity was measured on a five-point scale (0= **Definitely* not, 4= Definitely* *yes*; Lewczuk et al., 2017). Frequency of sexual behaviours (pornography use, porn-free masturbation, partnered sex) were all measured on a nine-point scale (1= *Never*, 9= *Multiple times per day)*.

**Table 2:** Abbreviations, descriptive statistics, and group comparisons for variables included in the network.

Variables	Abbreviation	Sample 1 (*N*= 1,356) *M (SD)*	Sample 2 (*N*= 944) *M (SD)*	Mann-Whitney *U*/ Independent samples *t-test*
***Community 1 (PPU): BPS items***				
BPS sum score	BPS	2.82 (2.94))	2.85 (2.93)	*W*= 649165, *p*= .55, *r*= .01, *V*=0.07
Using pornography more than you want to	BPS_1	0.64 (0.67)	0.65 (0.67)	*W*= 648724, *p*= .54 , *r*=.01, *V*=0.02
Unsuccessful attempts to cut back/quit	BPS_2	0.47 (0.67)	0.46 (0.66)	*W*= 633966, *p*= .65, *r*<.01, *V*=0.01
Difficulty resisting strong urges	BPS_3	0.56 (0.72)	0.60 (0.71)	*W*= 662688, *p*= .10, *r*=.04, *V*=0.04
Using pornography to cope with strong emotion	BPS_4	0.58 (0.74)	0.54 (0.71)	*W*= 622960, *p*= .22, *r*=.-.03, *V*=0.04
Continue using pornography despite guilt	BPS_5	0.57 (0.73)	0.60 (0.75)	*W*= 652926, *p*= .35, *r*=.02, *V*=0.02
***Community 2: Consumption patterns***				
Escalating time (quantitative tolerance)	Tol_quant	2.43 (1.52)	2.43 (1.51)	*W*= 645191, *p*=0.74, *V*=0.01
Escalating content/genres (qualitative tolerance)	Tol_qual	2.46 (1.68)	2.58 (1.69)	*W*= 669088, p= .05, V= 0.05
Pornographic binges (frequency)	Binge	1.02 (1.56)	0.87 (1.41)	*W*= 611713, *p*= .04, *r*=-.04, *V*=0.05
Edging^a^	Edging	2.99 (1.10)	3.00 (1.09)	*t*(2,320)= 0.14, *p*= .89, *d*=0.04
Tab-jumping	Tab_jump	3.30 (1.10)	3.26 (1.14)	*W*=631987 , *p*= .59, *r*=-.01, *V*=0.03

*Note*: *p<.05. BPS = Brief Pornography Screener (Kraus et al., 2020).

a Group comparison for *Edging* was calculated with independent-samples *t*-test given acceptable normality and equality of variances. Binges with pornography was measured on a nine-point scale (1= *Never*, 9= *Multiple times per day*). Participants with no history of pornographic binges were recoded as ‘0’ (see methods section).

**Table 3:** Network characteristics (edges retained, mean edge weight, and cross-community edges) for Models 1 and 2 across both samples.

Sample		Model 1				Model 2		
		Edges retained	Mean edge weight	Cross-community edges		Edges retained	Mean edge weight	
Sample 1		41/45 (91%)	0.10	22/41 (54%)		15/15 (100%)	0.16	
Sample 2		35/45 (78%)	0.10	15/35 (43%)		13/15 (87%)	0.16	

*Note*. Cross-community edges not calculated for Model 2 given that the PPU community consisted of a single node (BPS composite score).

## Data Availability

Data will be made available on reasonable request.
