# Supplementary material for: Problematic pornography use and novel patterns of escalating use: a cross-sectional network analysis with two Independent samples
Source: Addict Behav. Author manuscript; Available in PMC 2024 May 29. (PMC7616041; doi:10.1016/j.addbeh.2024.108048)

Supplementary Materials

Appendix A: Quality checks from online survey

Appendix B: Tabularised edge weights for Models 1 and 2

Appendix C: Bootstrapped results

Supplementary Figure C1. Bootstrapped 95%CI edge weights

Supplementary Figure C2. Bootstrapped differences for all edge weights

Supplementary Figure C3. Differences in node centrality (expected influence)

Supplementary Figure C4. Case-dropping bootstrap for node centrality (expected influence)

Supplementary Figure C5. Bootstrapped estimates for bridge expected influence

Supplementary Figure C6. Bootstrapped difference test for bridge expected influence

Supplementary Figure C7. Case-dropping bootstraps for bridge expected influence

Appendix D: Sensitivity analyses

Sensitivity analysis 1 (SA1): Networks re-estimated as mixed graphical models.

Sensitivity analysis 2 (SA2): Networks re-estimated as mixed graphical models with paranormal transformation.

Sensitivity analysis 3 (SA3): Models re-estimated after removing time-related PPU items

Sensitivity analysis 4 (SA4): Node redundancy (Goldbricker) and community detection (Walktrap algorithm)

Sensitivity analysis 5 (SA5): Comparing networks after removing scores of BPS=0

Sensitivity analysis 6 (SA6) Comparing networks for individuals below and above BPS cut-off score (BPS≥4).

Appendix A: Quality checks from online survey

	**Sample 1** Final *N*= 1,356	**Sample 2** Final *N*= 944	**Total** Final *N*= 2,300
No recent use	6	6	12
Incomplete responses	48	0	48
Inconsistent country - screener vs survey	2	0	2
Inconsistent sex - screener vs survey	25	2	27
Unrealistic duration (<7 mins completion time) and/or straight lining	8	2	10
Self-rating of response quality	4	2	6
Attention checks	34	14	48
Not completed same survey on other platforms	0	0	0
**Total**	127	26	153

Table S1. Number of individuals removed following quality checks. Note: Recent use was defined as using pornography at least once in the previous 12 months; Inconsistent responses (sex/country) refers to a different response to the Qualtrics survey compared to the screener hosted by the respective crowdsourcing platform (Sample 1: *Connect* by CloudResearch [USA only]; Sample= Prolific [UK only]); Self-rating of quality was determined from responses to the following question: "*Overall, how would you describe your participation in this entire survey?*" (1= *I answered every question carefully and honestly*., 3= *I answered some questions dishonestly or randomly*, 5= *I randomly responded and/or did not respond honestly to any questions*) whereby scores of 3-5 led to participant removal. Participants were required to accurately respond to at least two of the three attention checks embedded in the survey. To ensure that respondents did not complete the survey on multiple platforms, a final item asked whether they had already completed the survey on another Crowdsourcing site.

Appendix B: Tabularised edge weights for Models 1 and 2

Table B1. Edge weights for estimated network (Model 1, Sample 1).

	BPS_1	BPS_2	BPS_3	BPS_4	BPS_5	Tol_quant	Tol_qual	Binge	Tab_jump	Edging
**BPS_1**	-									
**BPS_2**	0.31	-								
**BPS_3**	0.21	0.37	-							
**BPS_4**	0.16	0.00	0.16	-						
**BPS_5**	0.13	0.28	0.25	0.22	-					
**Tol_quant**	0.11	0.06	0.01	0.07	0.11	-				
**Tol_qual**	0.05	0.00	0.10	0.02	-0.01	0.39	-			
**Binge**	0.02	-0.06	0.12	0.11	-0.09	0.11	0.13	-		
**Tab_jump**	0.07	-0.06	0.02	0.06	-0.03	0.05	0.08	0.12	-	
**Edging**	0.00	0.02	0.00	0.01	0.02	0.04	0.01	0.26	0.33	-

Table B2. Edge weights for estimated network (Model 1, Sample 2).

	BPS_1	BPS_2	BPS_3	BPS_4	BPS_5	Tol_quant	Tol_qual	Binge	Tab_jump	Edging
**BPS_1**	-									
**BPS_2**	0.31	-								
**BPS_3**	0.29	0.32	-							
**BPS_4**	0.09	0.07	0.04	-						
**BPS_5**	0.10	0.35	0.18	0.31	-					
**Tol_quant**	0.17	0.01	0.00	0.02	0.14	-				
**Tol_qual**	-0.05	0.00	0.14	0.14	-0.02	0.41	-			
**Binge**	0.07	0.00	0.00	0.15	0.00	0.09	0.10	-		
**Tab_jump**	0.00	-0.04	0.04	0.02	0.00	0.08	0.10	0.09	-	
**Edging**	0.08	0.00	0.00	0.00	-0.03	0.05	-0.01	0.25	0.33	-

Table B3. Edge weights for estimated network (Model 2, Sample 1).

	BPS	Tol_quant	Tol_qual	Binge	Tab_jump	Edging
**BPS**	-					
**Tol_quant**	0.49	-				
**Tol_qual**	0.09	0.42	-			
**Binge**	0.06	0.11	0.16	-		
**Tab_jump**	0.02	0.05	0.09	0.14	-	
**Edging**	0.04	0.05	0.02	0.27	0.34	-

Table B4. Edge weights for estimated network (Model 2, Sample 2).

	BPS	Tol_quant	Tol_qual	Binge	Tab_jump	Edging
**BPS**	-					
**Tol_quant**	0.48	-				
**Tol_qual**	0.08	0.43	-			
**Binge**	0.14	0.10	0.15	-		
**Tab_jump**	0.00	0.08	0.11	0.10	-	
**Edging**	0.02	0.07	0.00	0.26	0.33	-

Appendix C: Bootstrapped results

Supplementary Figure C1. Bootstrapped 95%*CI* edge weights

Model 1

Sample 1

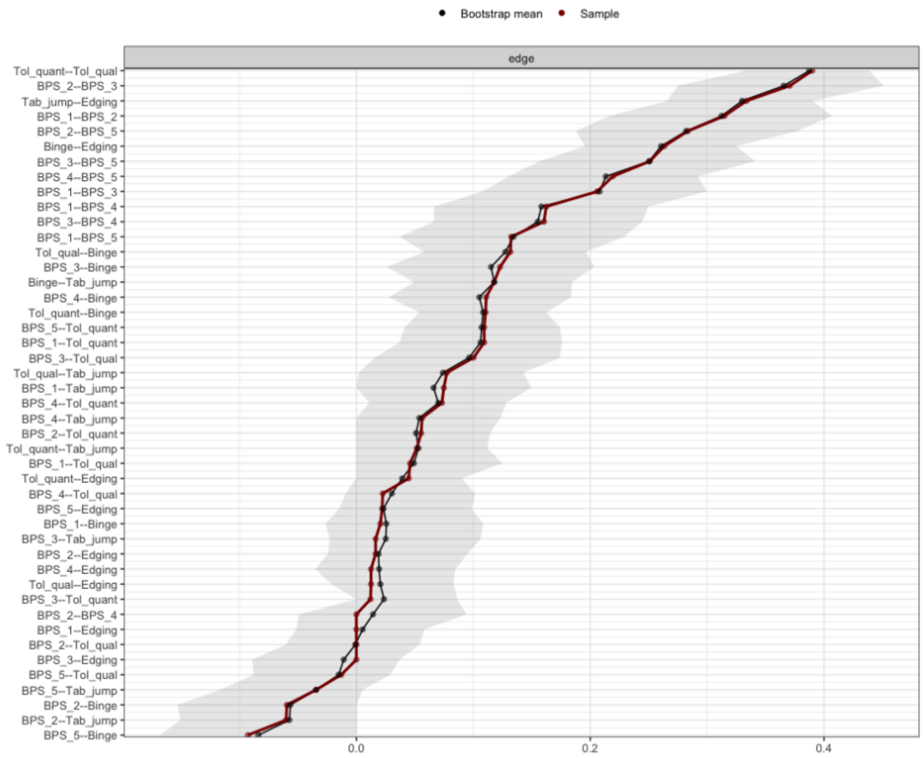

Sample 2

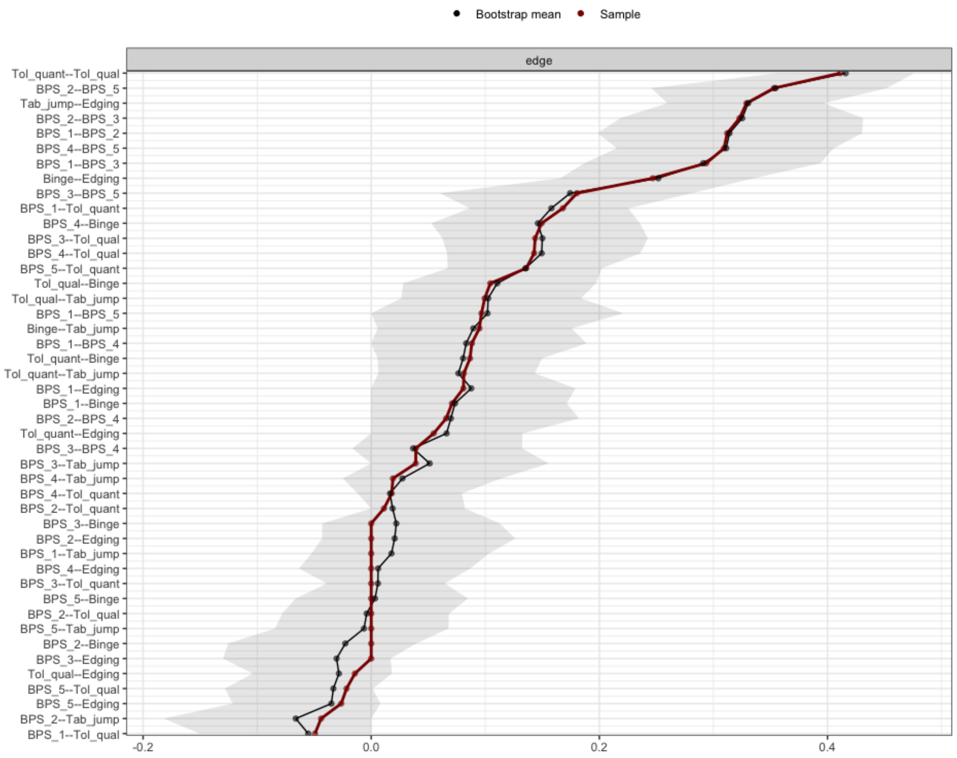

Model 2: BPS modelled as composite score

Sample 1

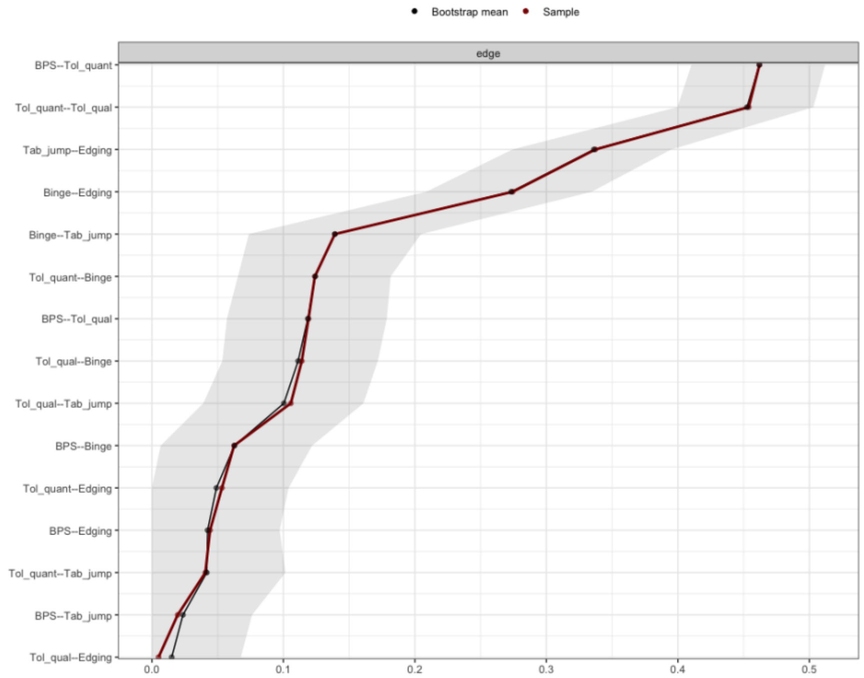

Sample 2

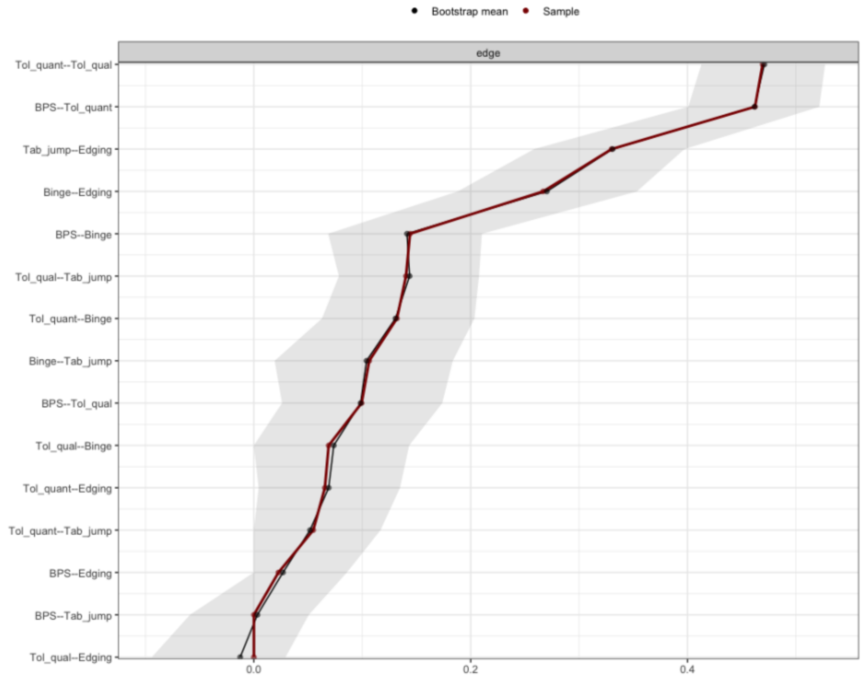

Supplementary Figure C2. Bootstrapped differences for all edge weights. Note: black boxes indicate a statistically significant difference in edge weights (*p*<.05), whereas grey boxes indicate no statistically significant difference.. Colour of boxes on the diagonal represent the magnitude of partial correlations between two edges (dark blue represents strong positive, dark red represents strong negative.**Model 1**

Sample 1 Sample 2

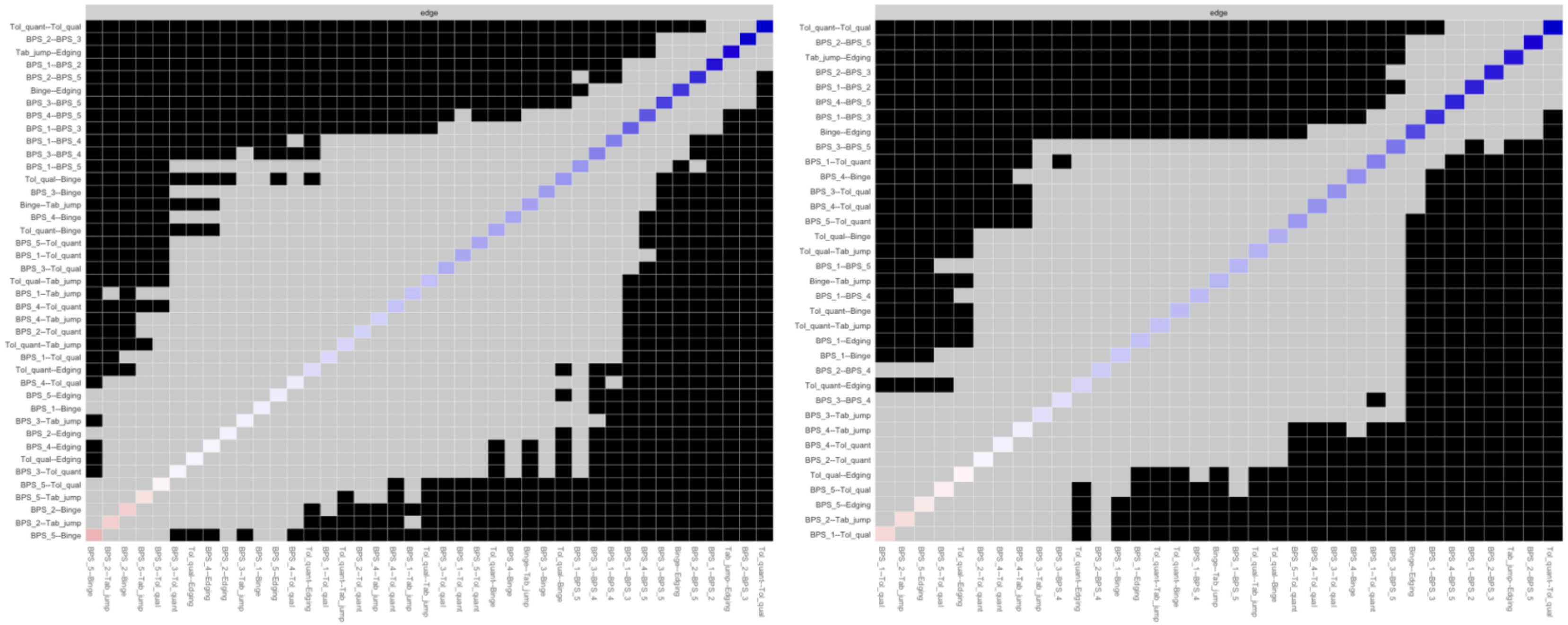

Model 2: BPS modelled as composite score

Sample 1

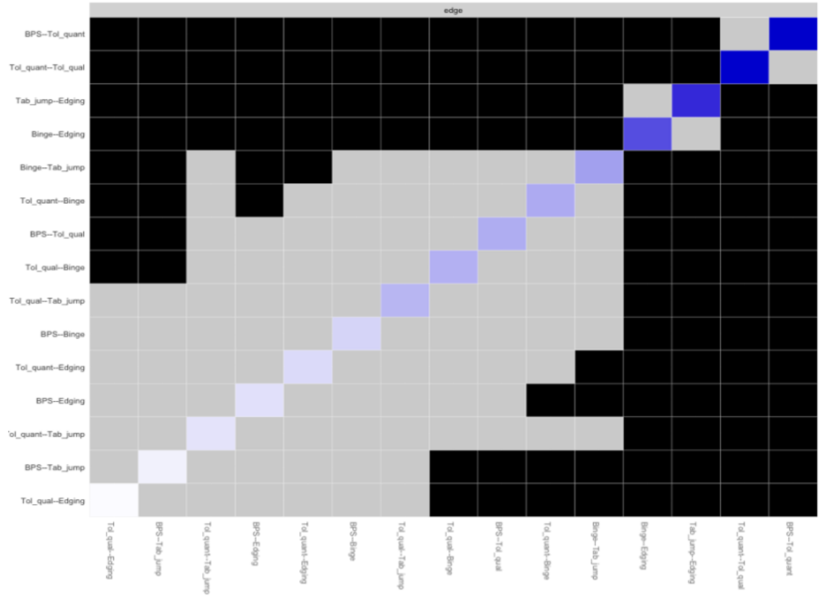

Sample 2

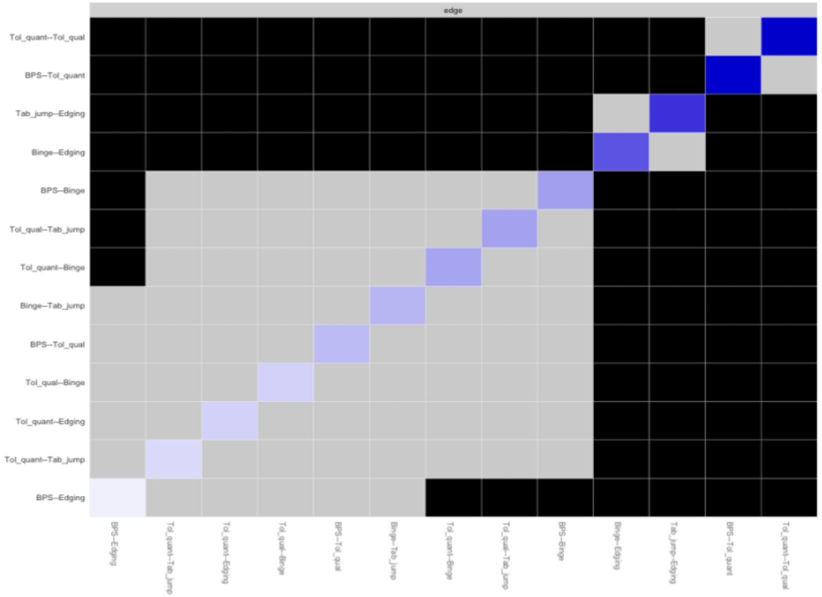

Supplementary Figure C3. Differences in centrality estimates (expected influence)

Model 1

Sample 1 Sample 2

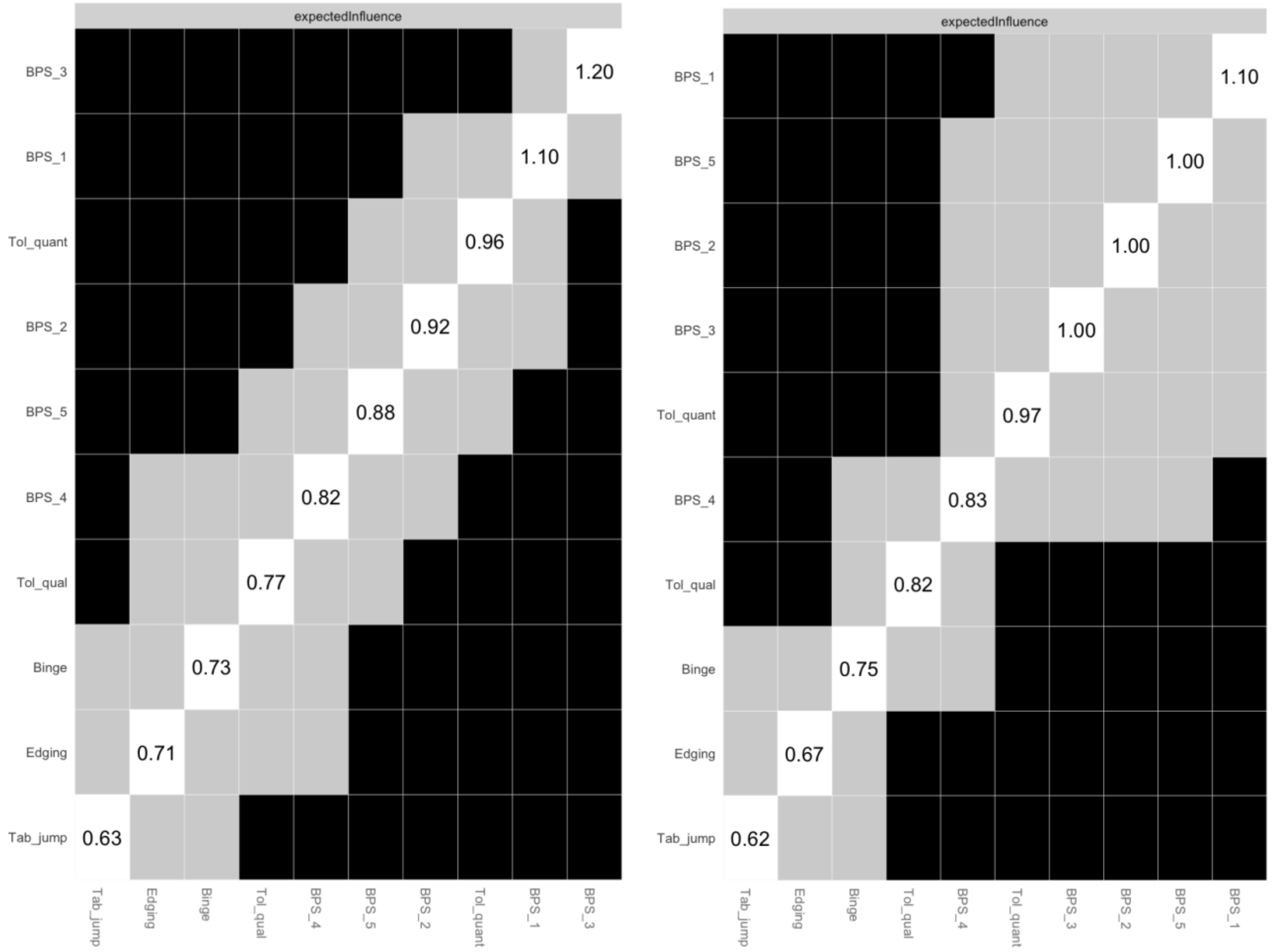

Model 2: BPS modelled as composite score

Sample 1

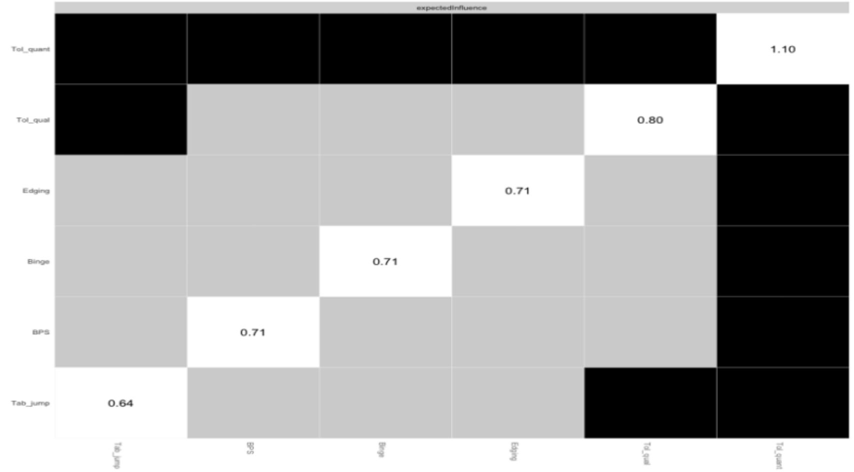

Sample 2

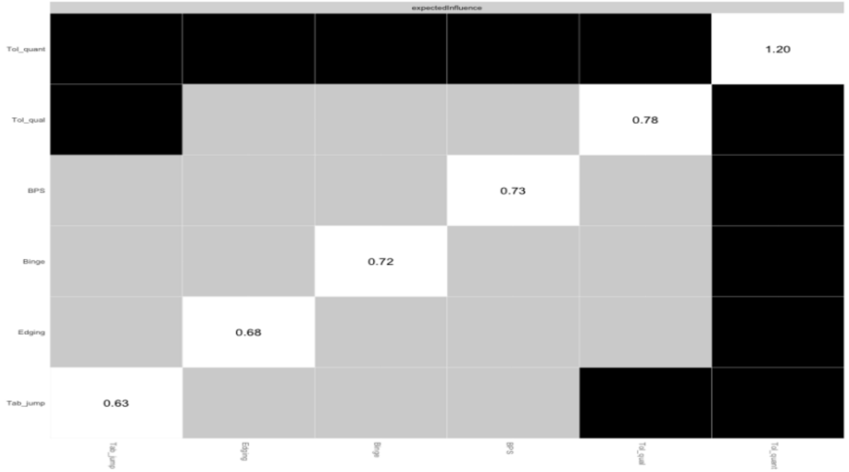

Supplementary Figure C4. Case-dropping bootstrap for centrality estimates (expected influence)

Model 1

Sample 1 | Sample 2

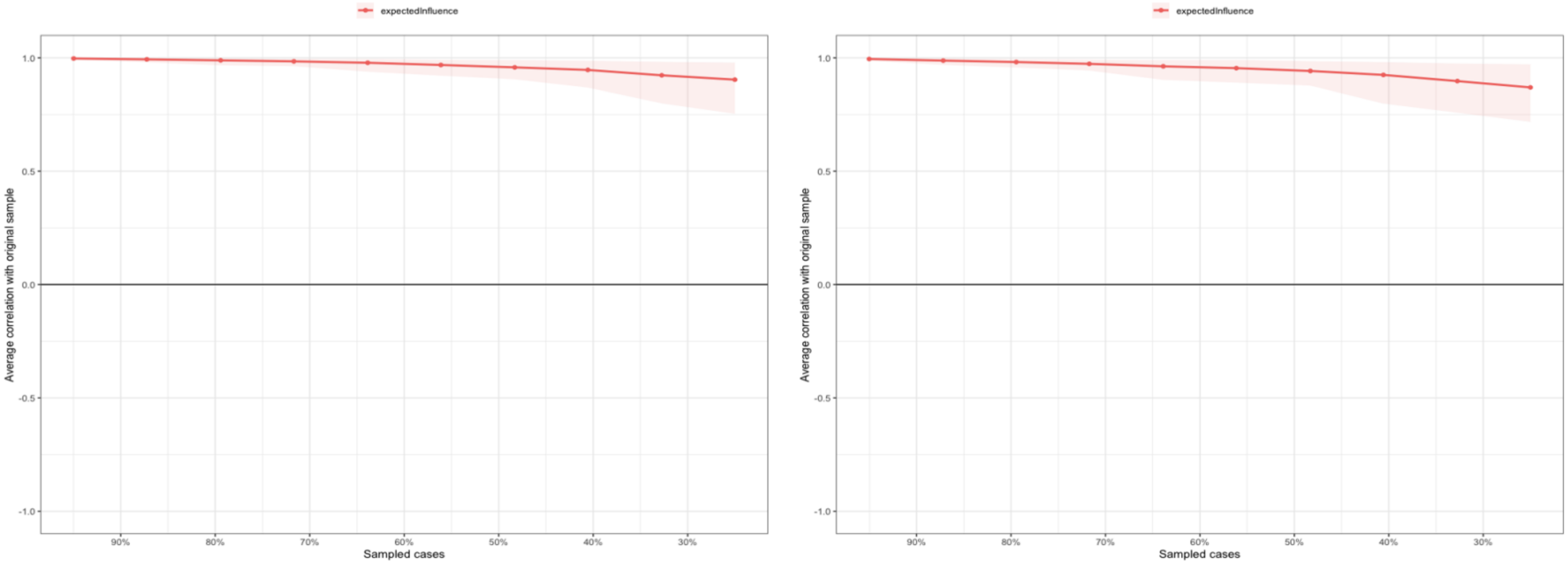

Model 2: BPS modelled as composite score

Sample 1

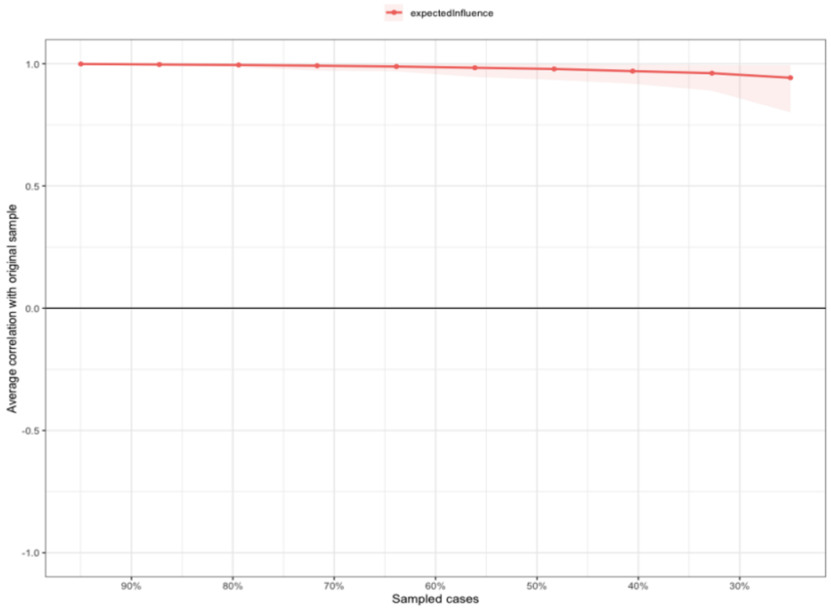

Sample 2

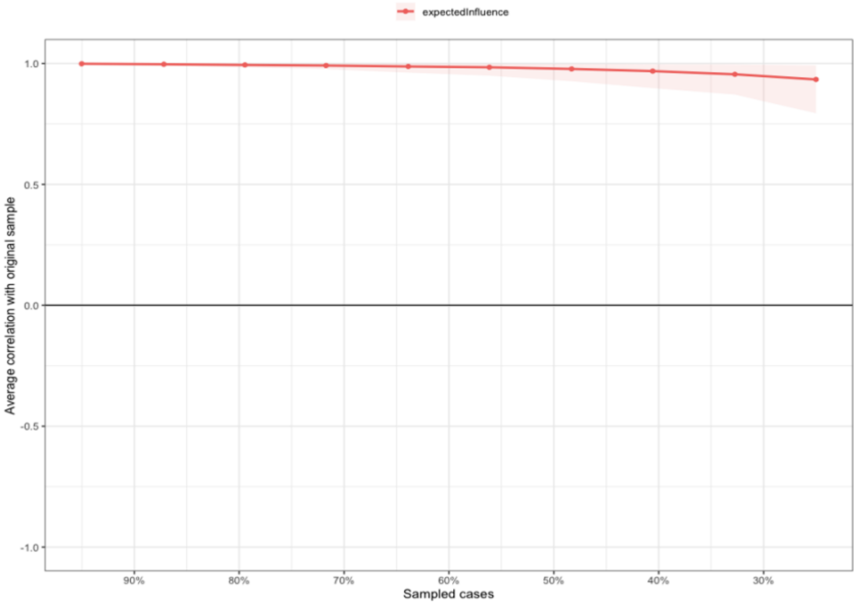

Supplementary Figure C5. Bootstrapped estimates for bridge expected influence

Model 1

Sample 1 Sample 2

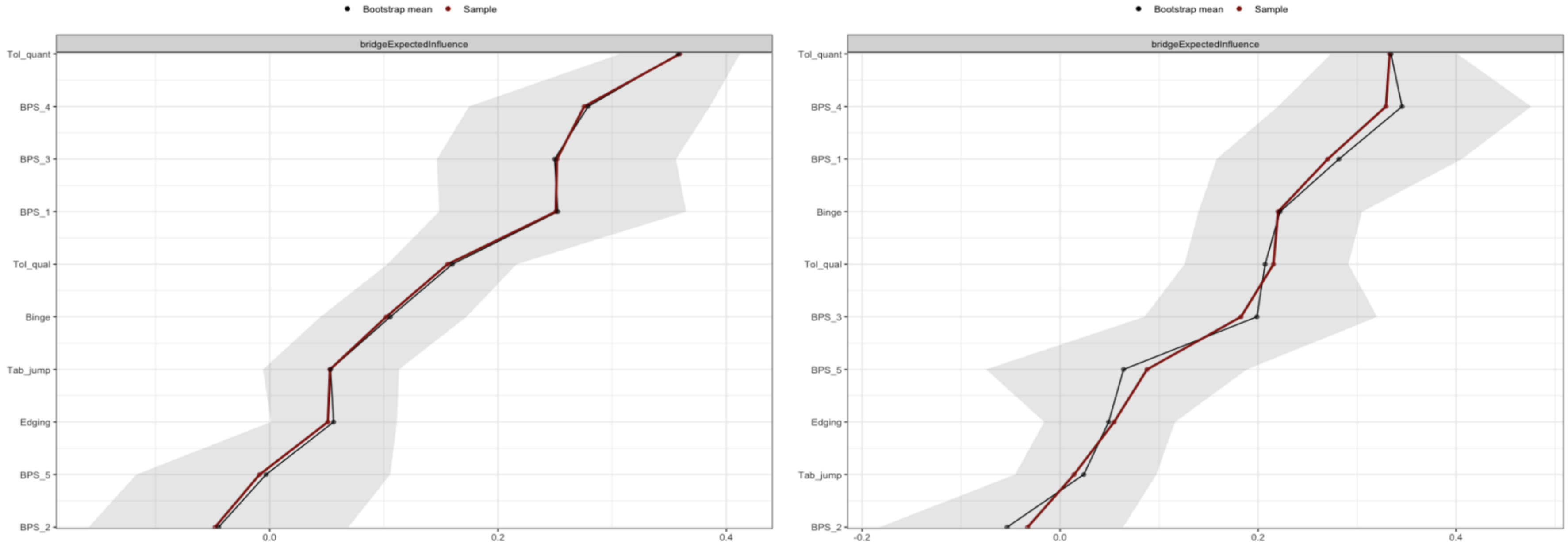

Supplementary Figure C6. Bootstrapped difference test for bridge expected influence

Model 1

Sample 1

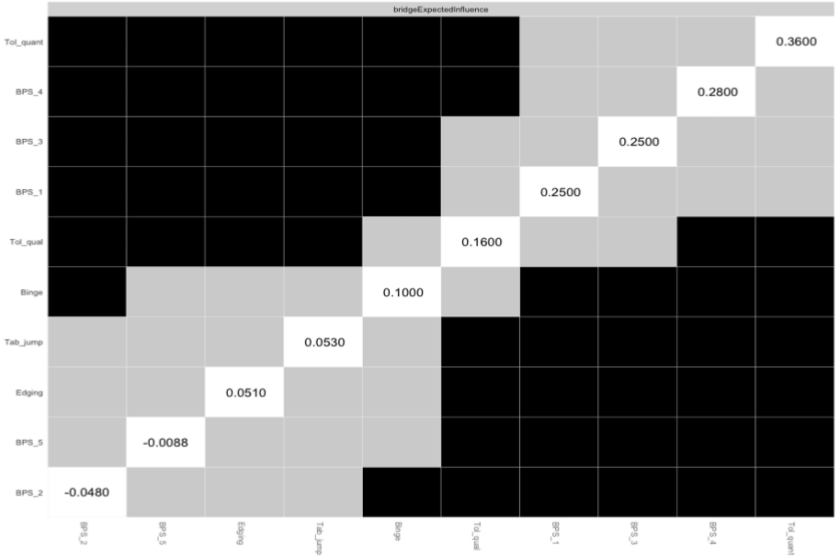

Sample 2

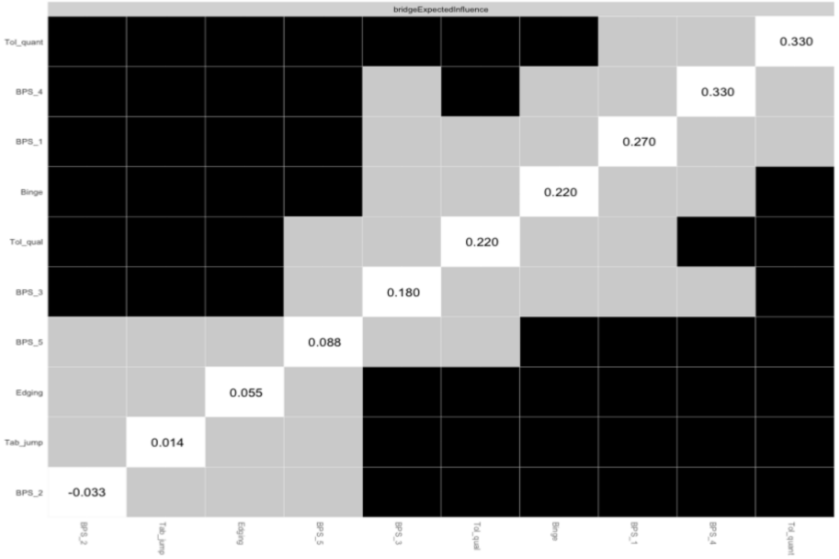

Supplementary Figure C7. Case-dropping bootstraps for bridge expected influence

Model 1

Sample 1 Sample 2

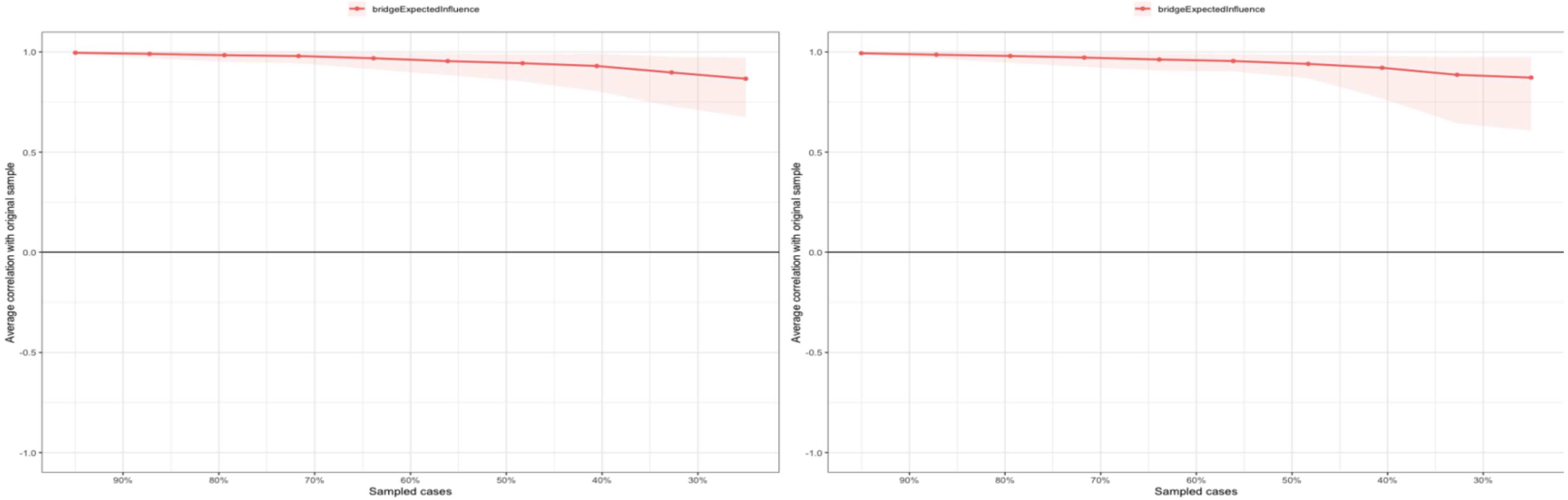

Appendix D: Sensitivity analyses

Sensitivity analysis 1 (SA1): Networks re-estimated as mixed graphical models.

Estimated networks

Model 1: BPS items modelled as separate nodes

Sample 1

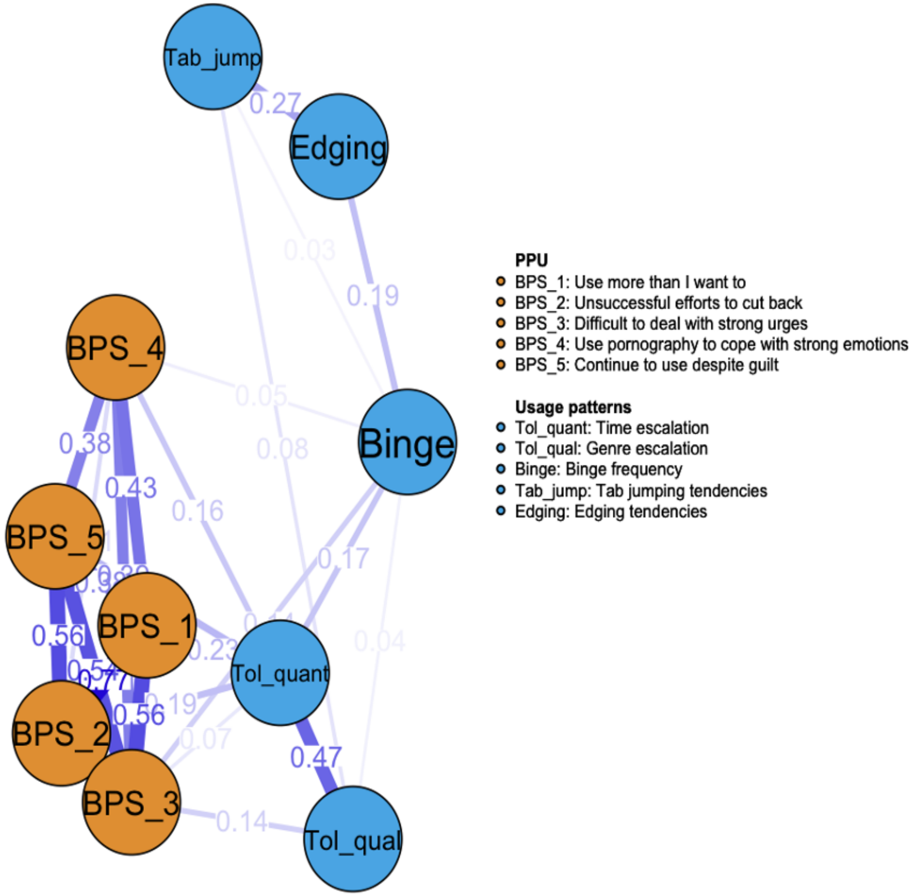

Sample 2

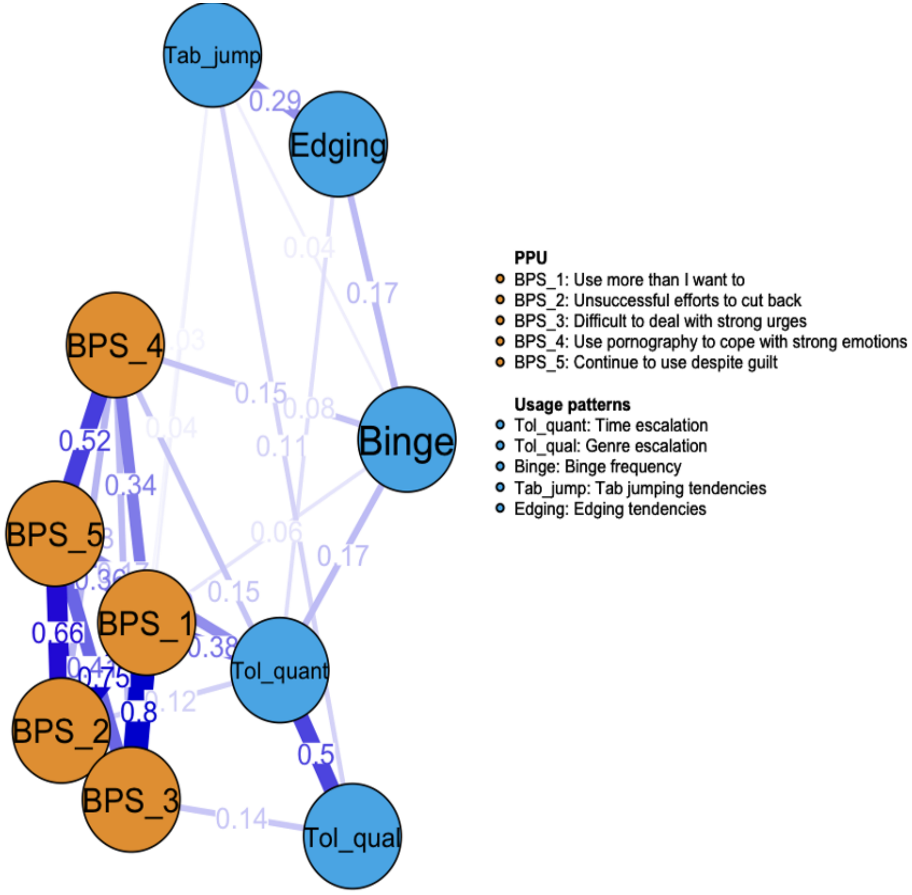

Model 2: BPS modelled as composite score

Sample 1

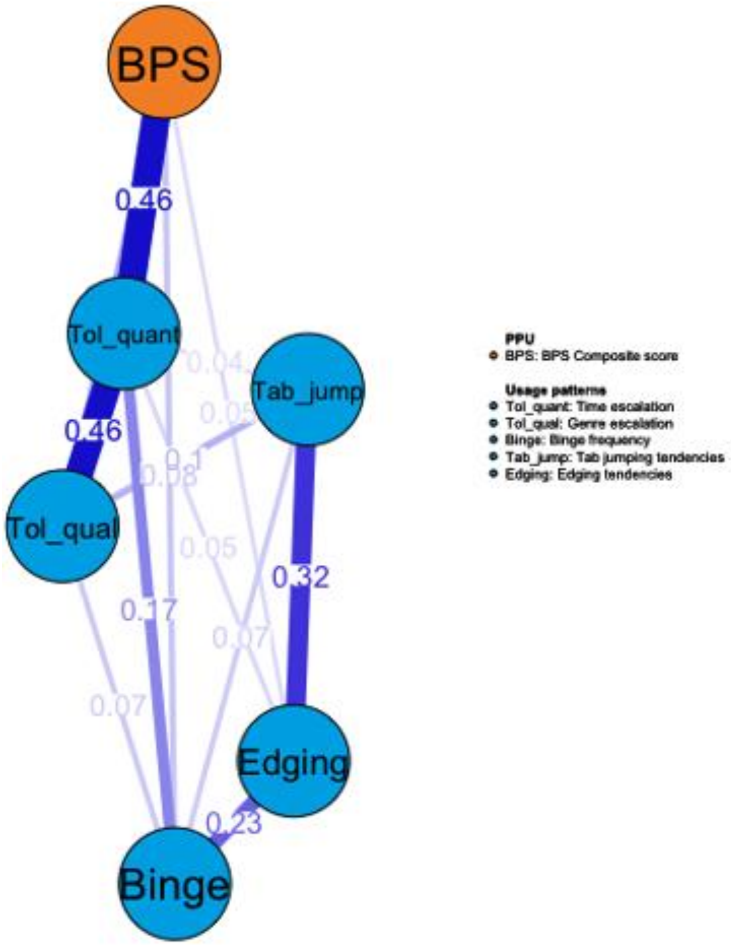

Sample 2

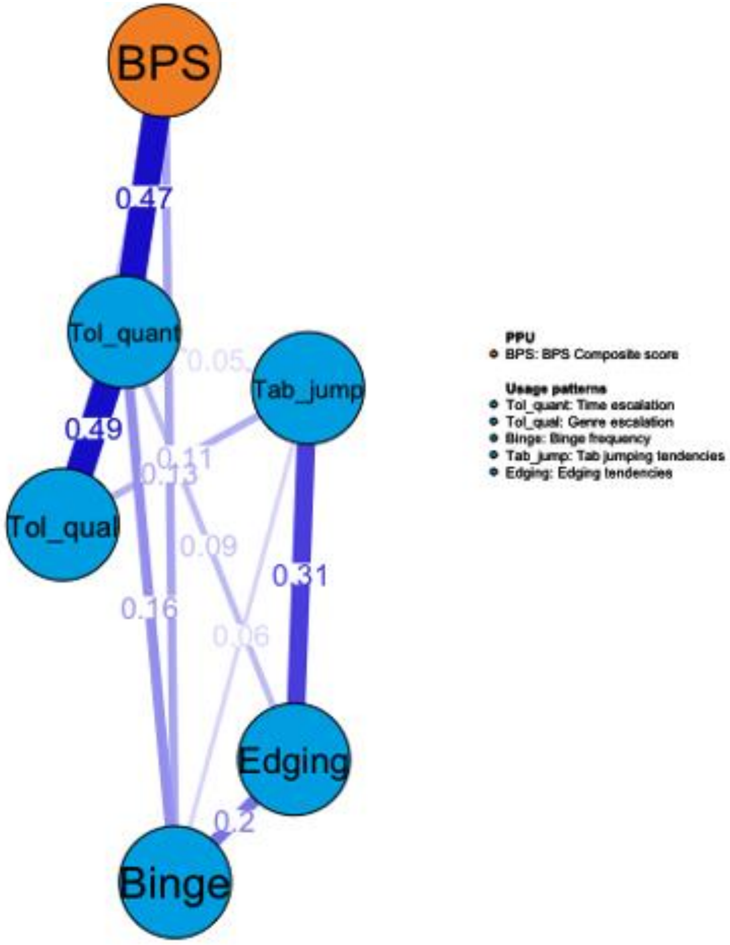

Node centrality

Model 1: BPS items modelled as separate nodes

Sample 1 Sample 2

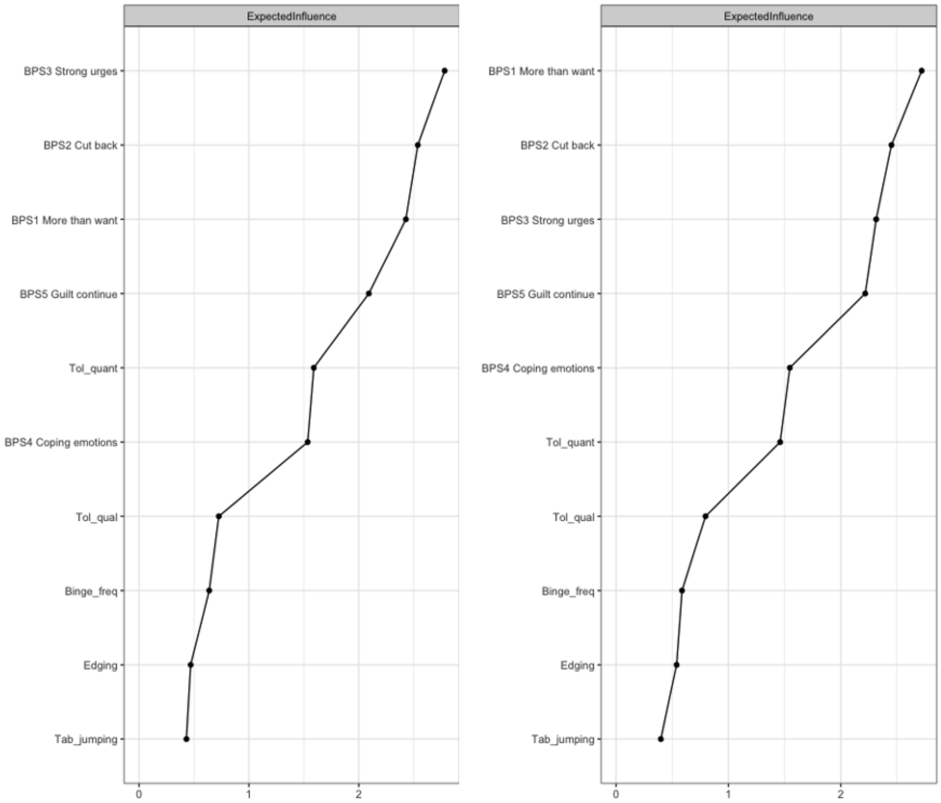

Model 2: BPS modelled as composite score

Sample 1 Sample 2

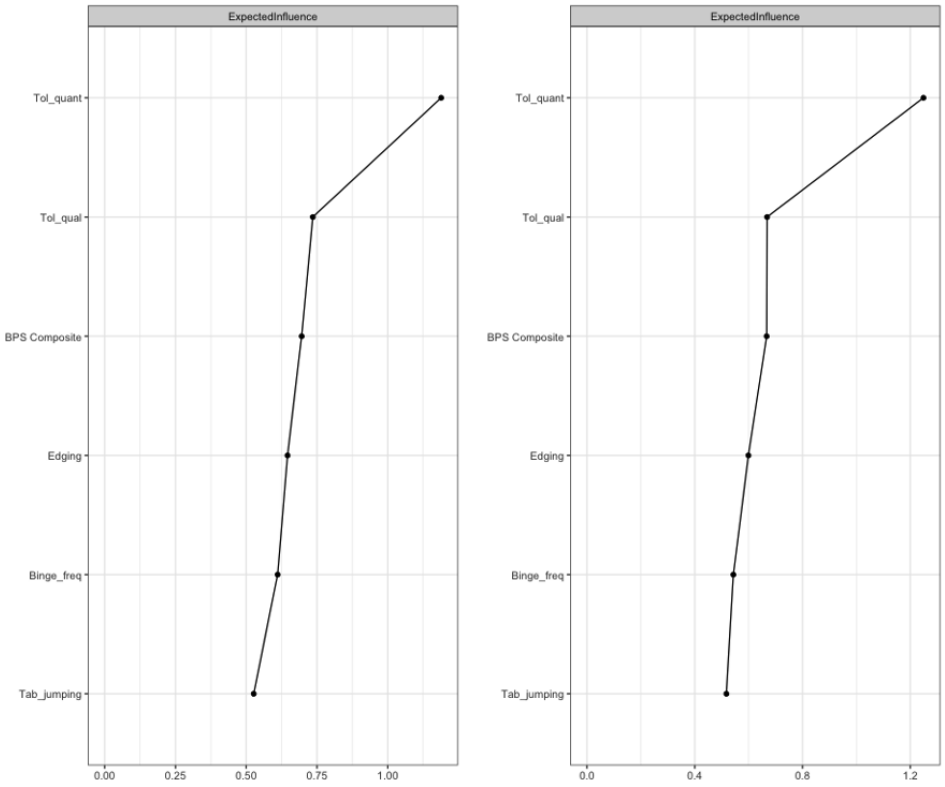

Bridge centrality

Model 1: BPS items modelled as separate nodes

Sample 1 Sample 2

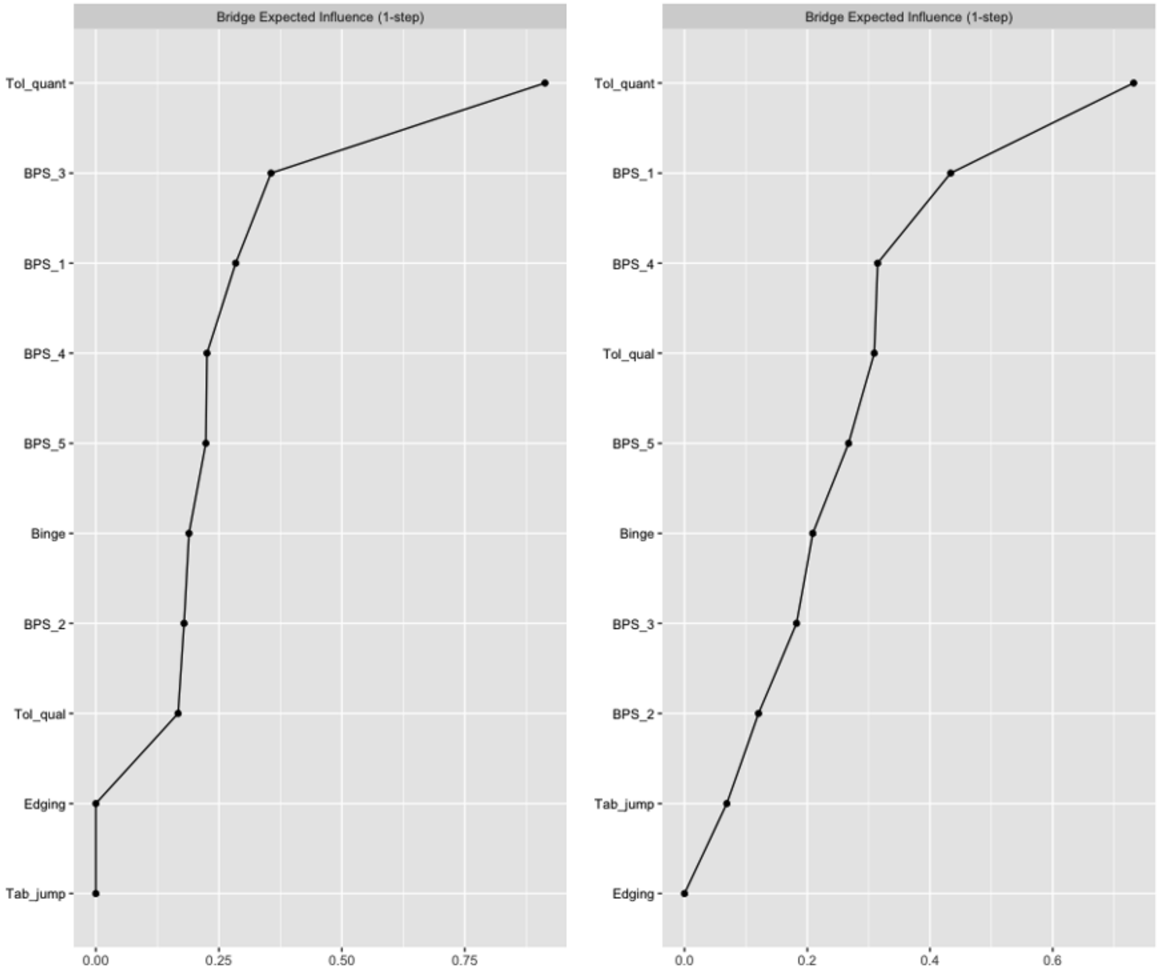

Sensitivity analysis 2 (SA2): Networks re-estimated as ***mixed graphical models with huge (paranormal) transformation.***

Estimated networks

Model 1: BPS items modelled as separate nodes

Sample 1

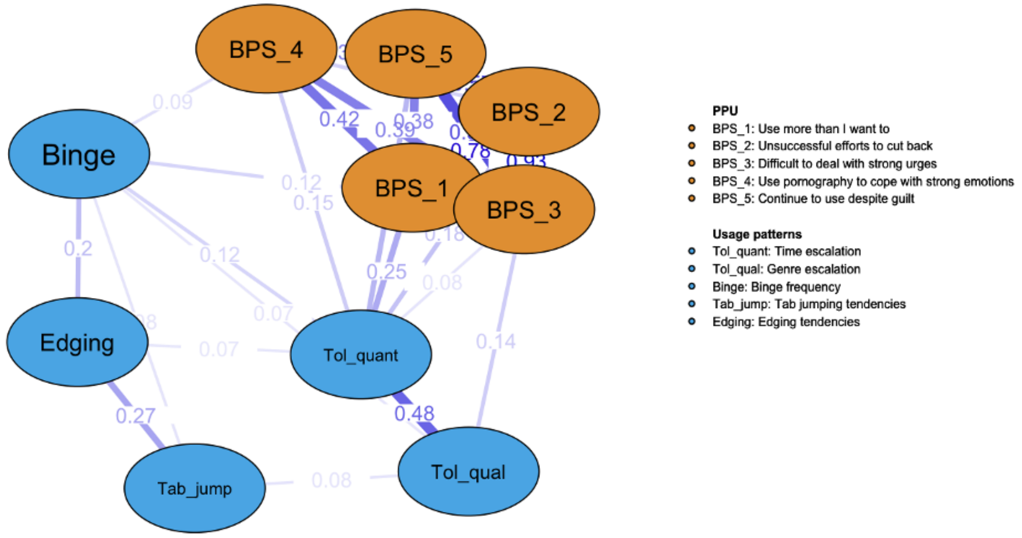

Sample 2

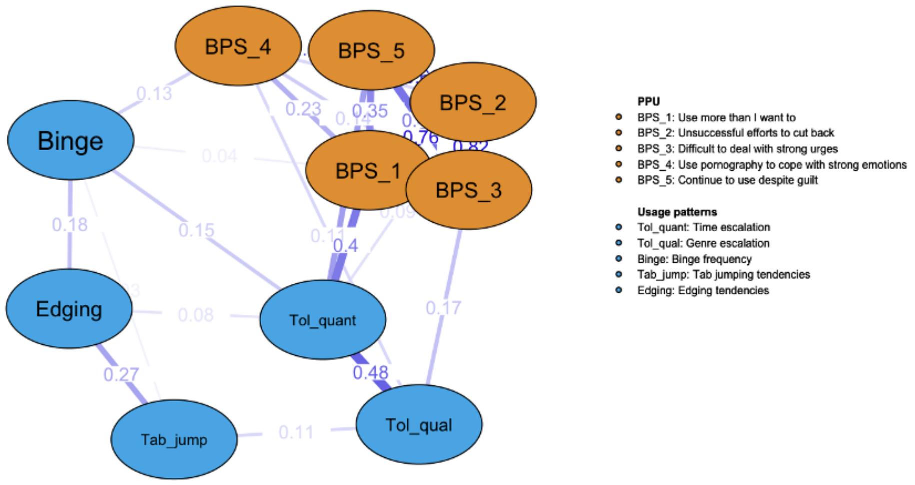

Model 2: BPS modelled as composite score

Sample 1

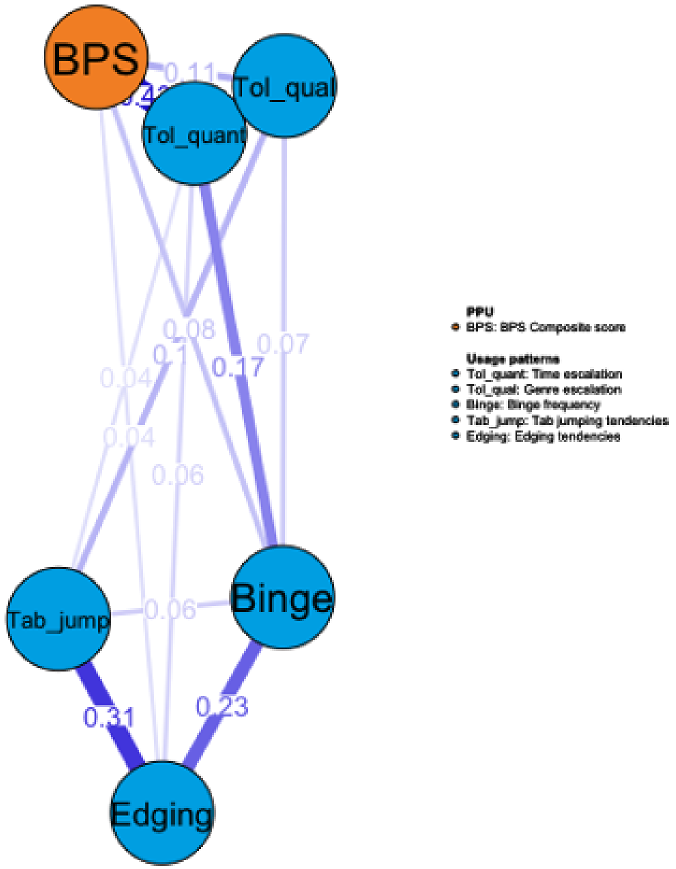

Sample 2

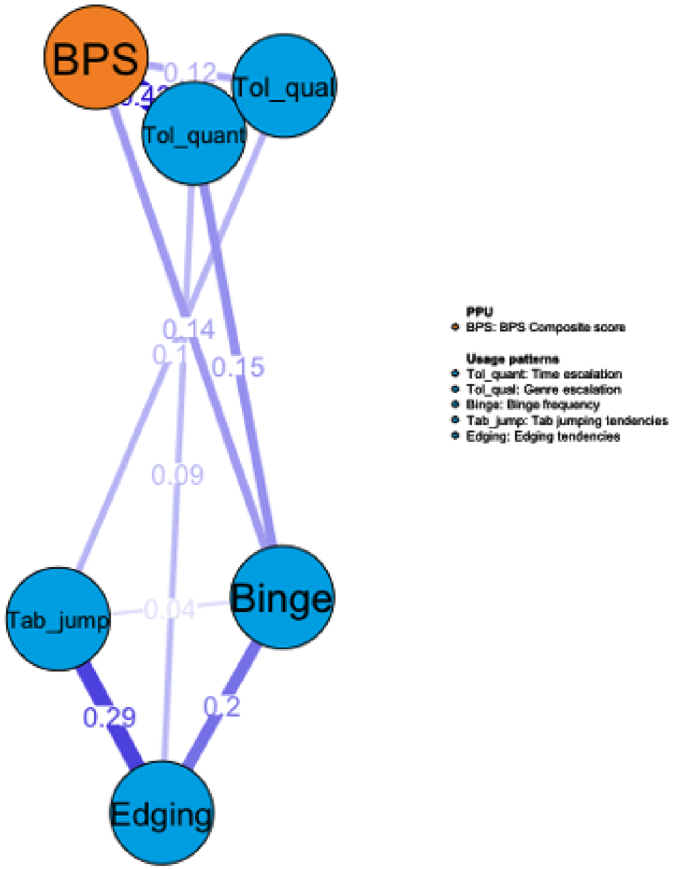

Node centrality

Model 1: BPS items modelled as individual nodes

Sample 1 Sample 2

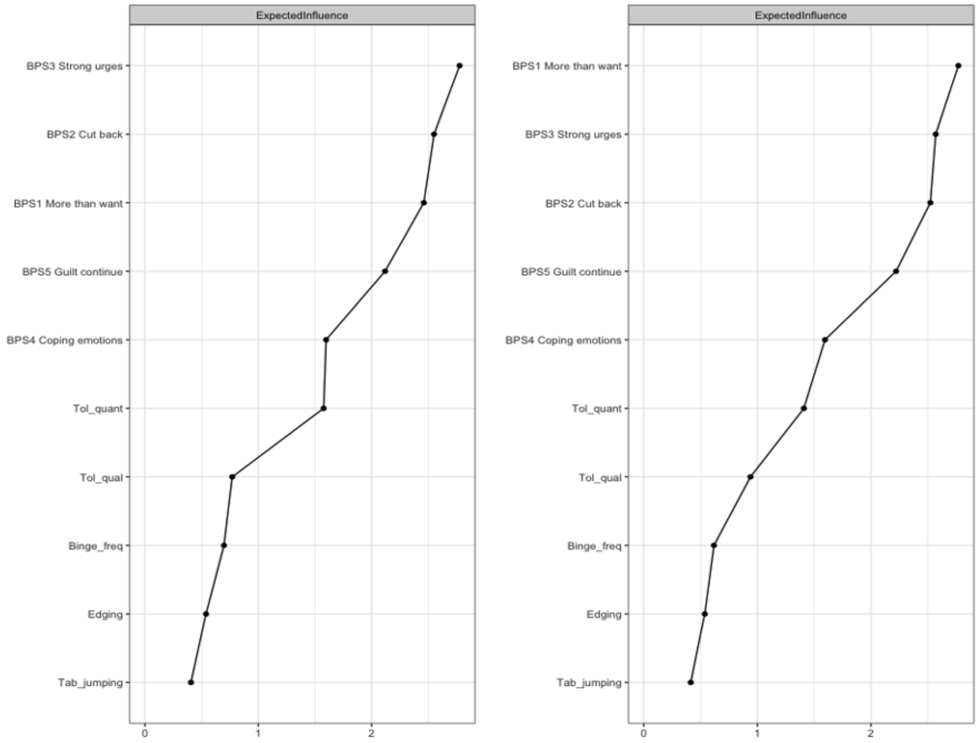

Model 2: BPS modelled as composite score

Sample 1 Sample 2

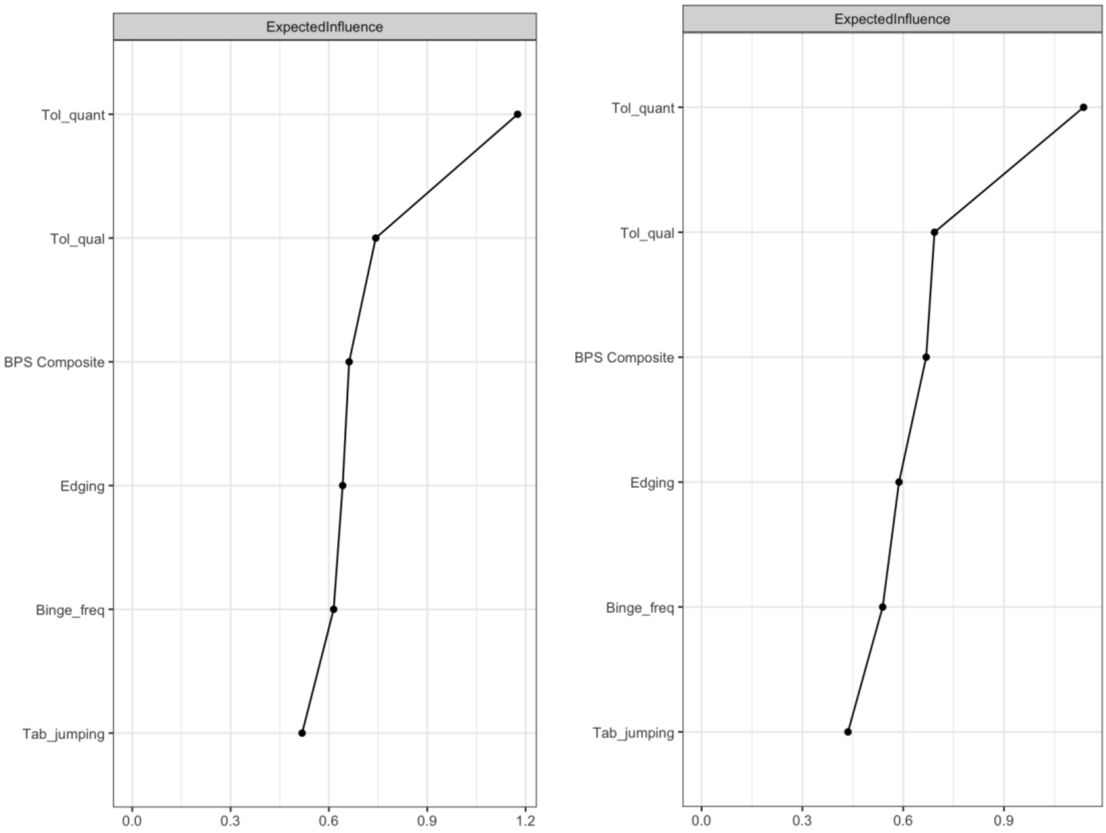

Bridge centrality

Model 1: BPS items modelled as individual nodes

Sample 1 Sample 2

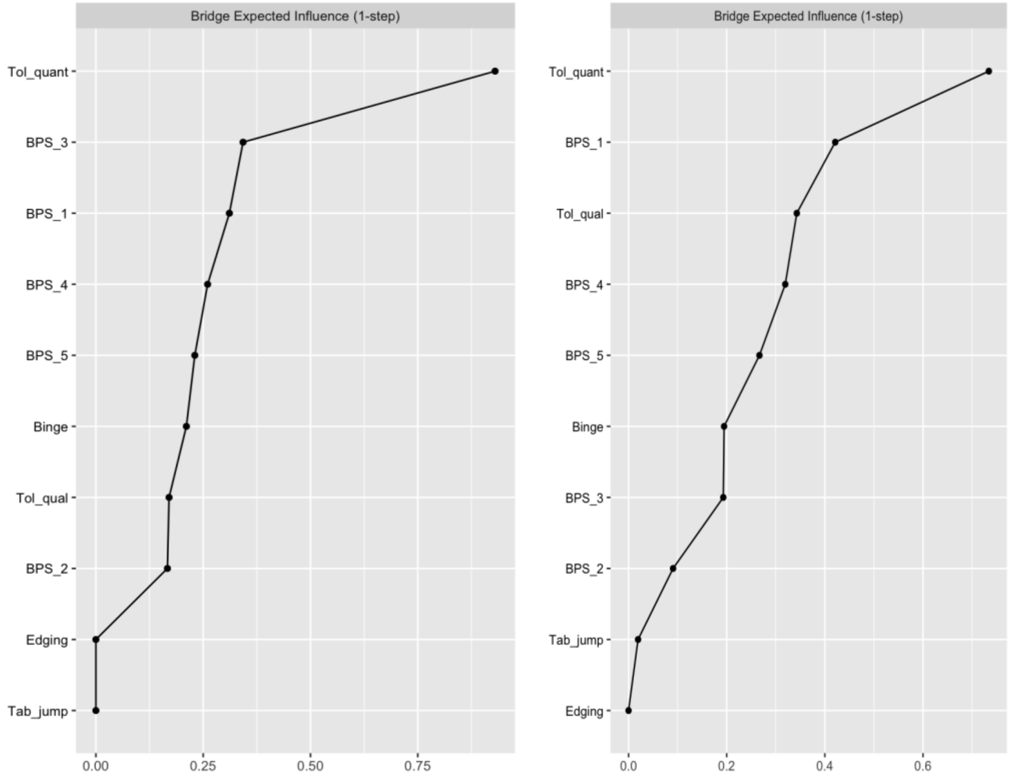

Sensitivity analysis 3 (SA3): Models re-estimated ***after removing time-related PPU items (BPS_1, BPS_2)***

Estimated networks

Model 1: BPS items included as separate nodes

Sample 1

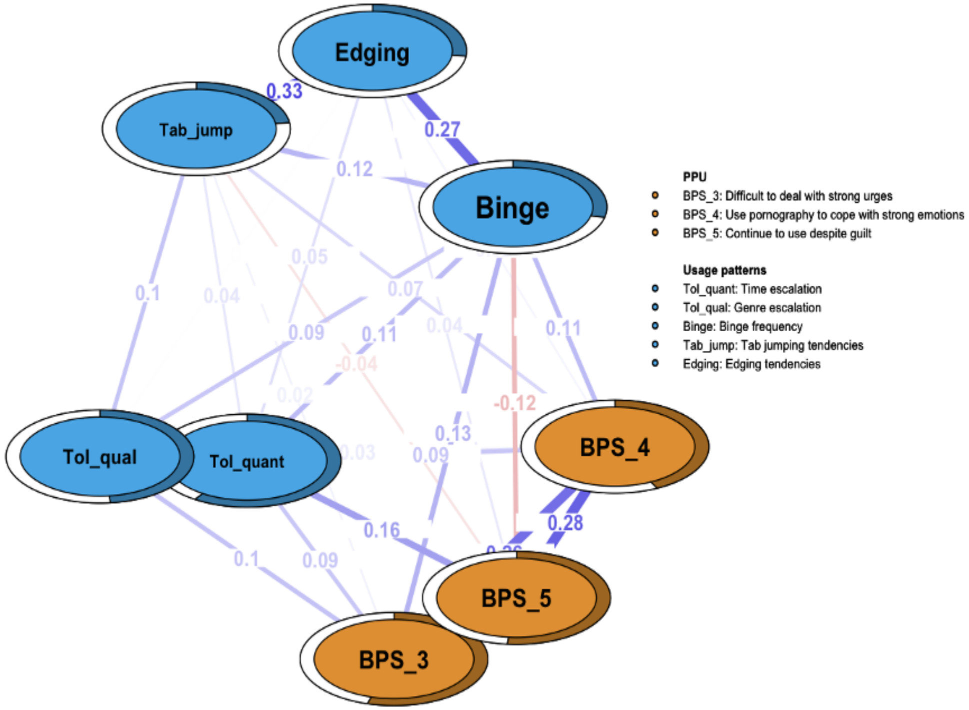

Sample 2

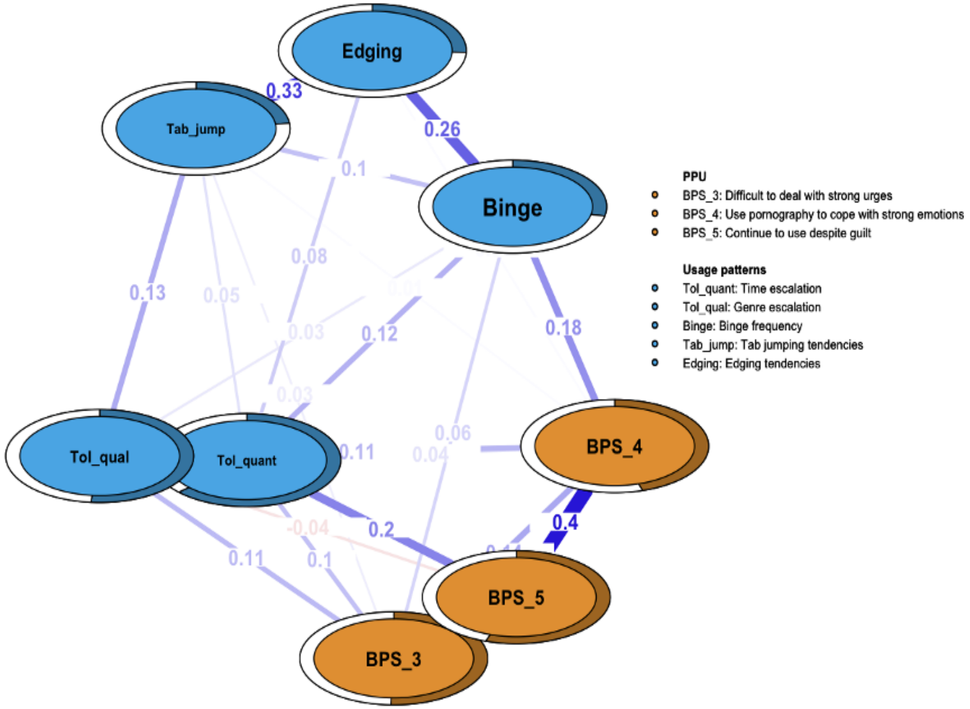

Model 2: BPS modelled as composite score

Sample 1

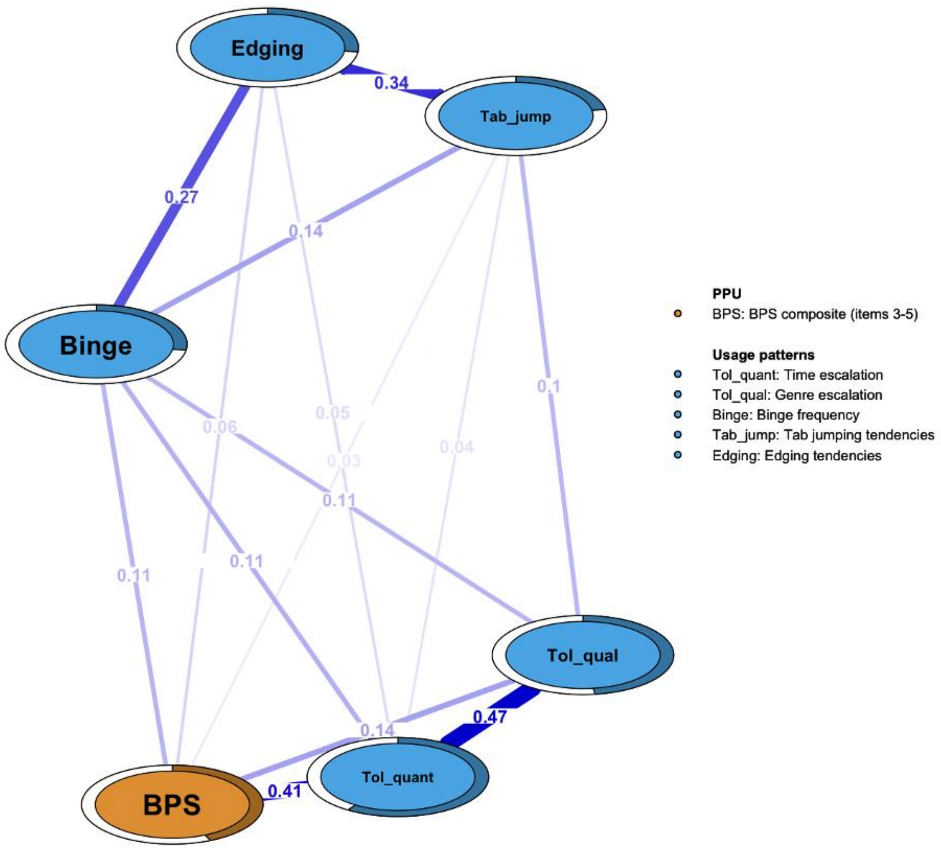

Sample 2

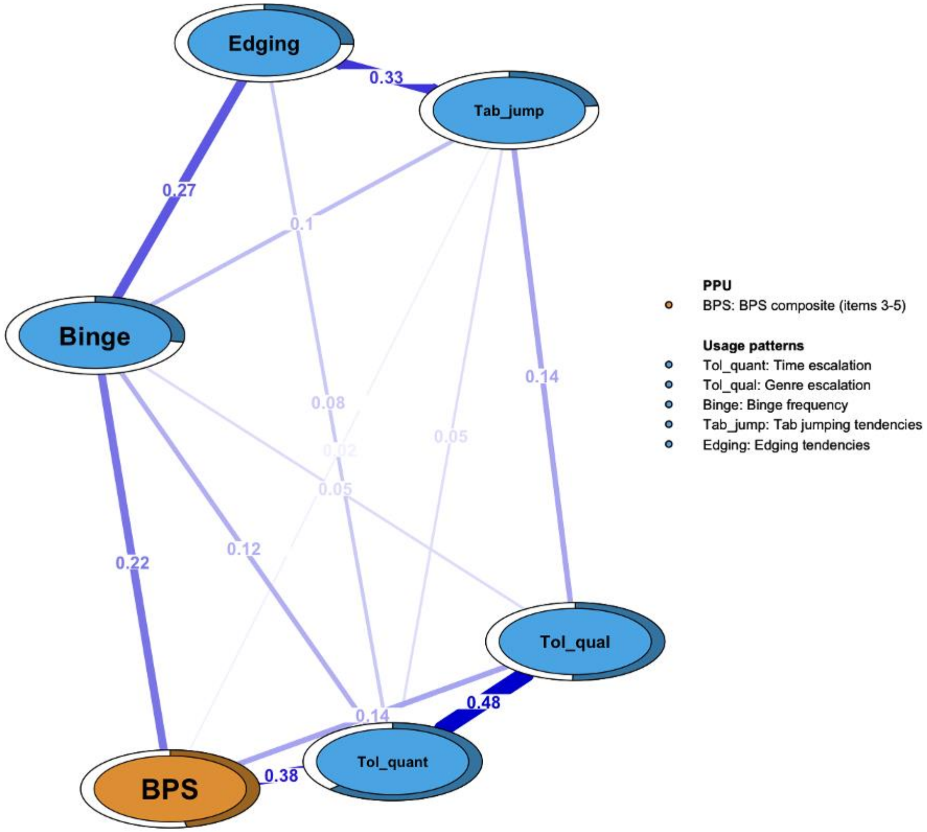

Node centrality

Model 1: BPS items modelled as individual nodes

Sample 1 Sample 2

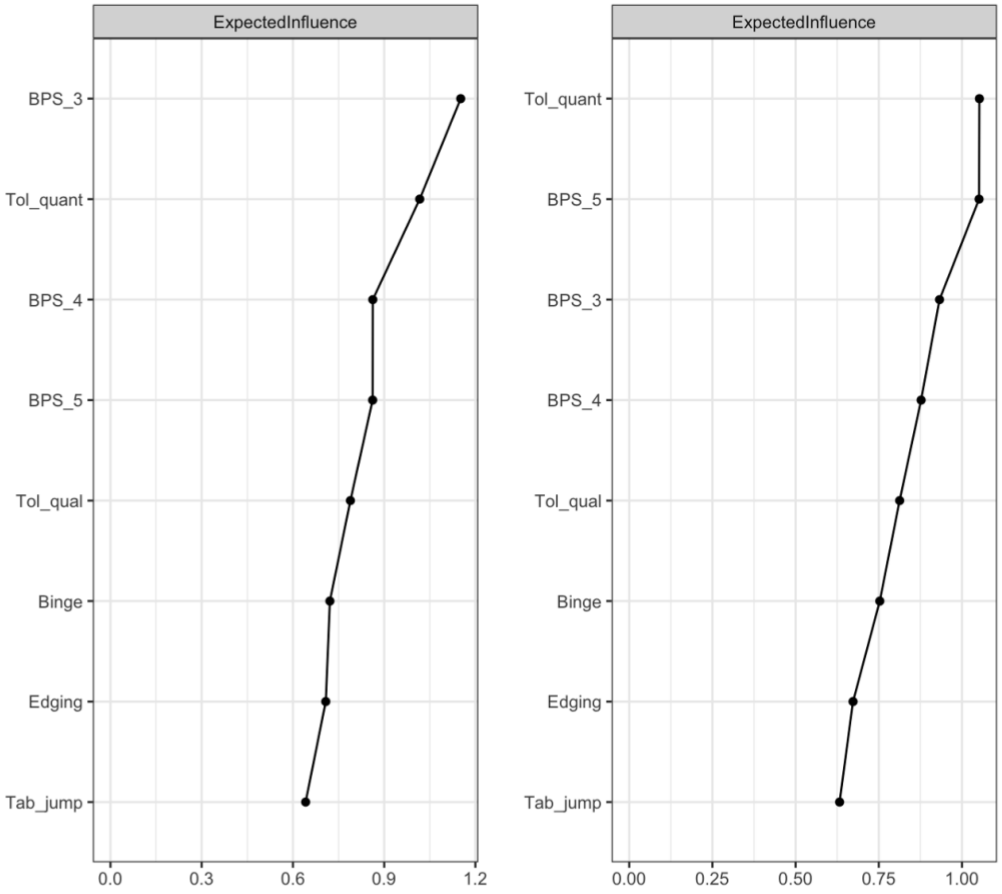

Sample 1 Sample 2

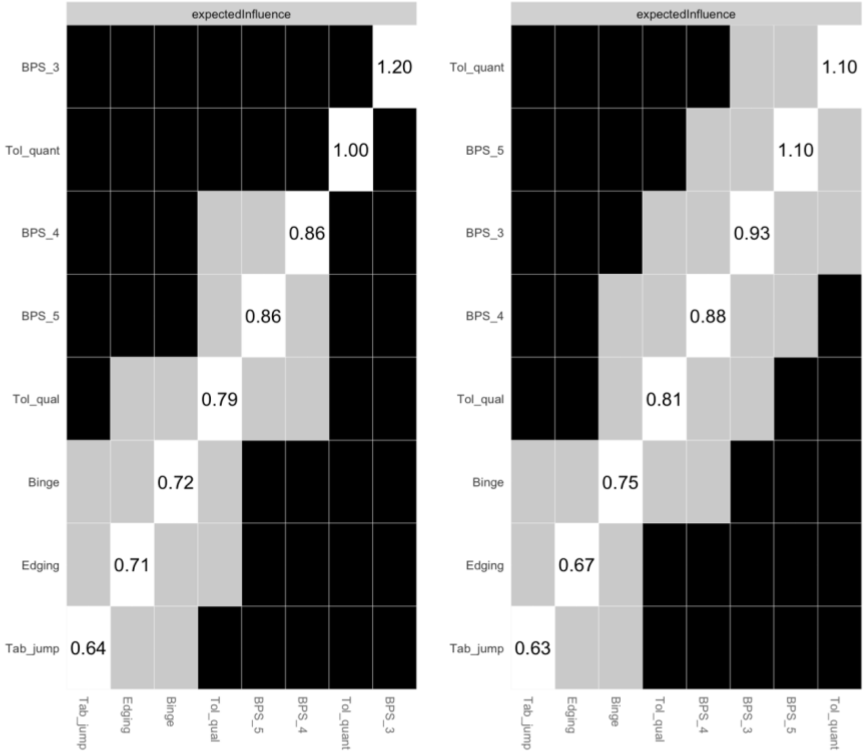

Model 2: BPS modelled as composite score

Sample 1 Sample 2

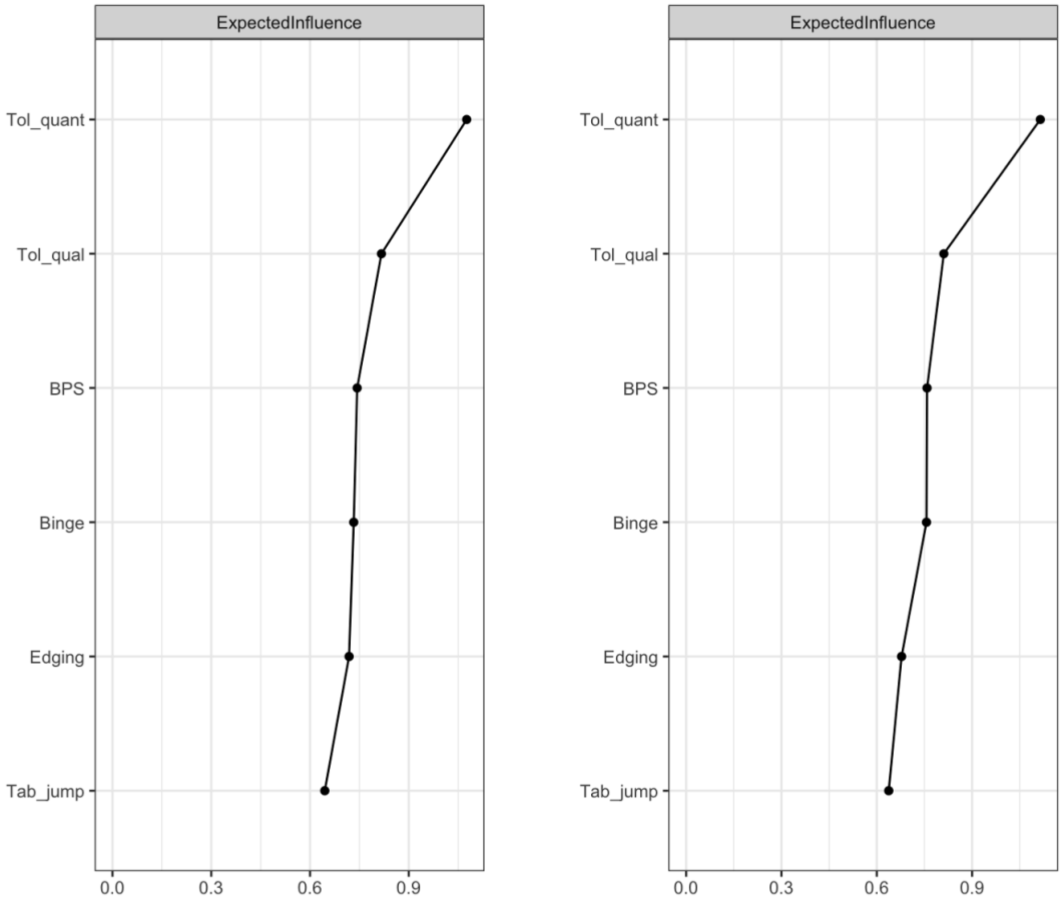

Sample 1 Sample 2

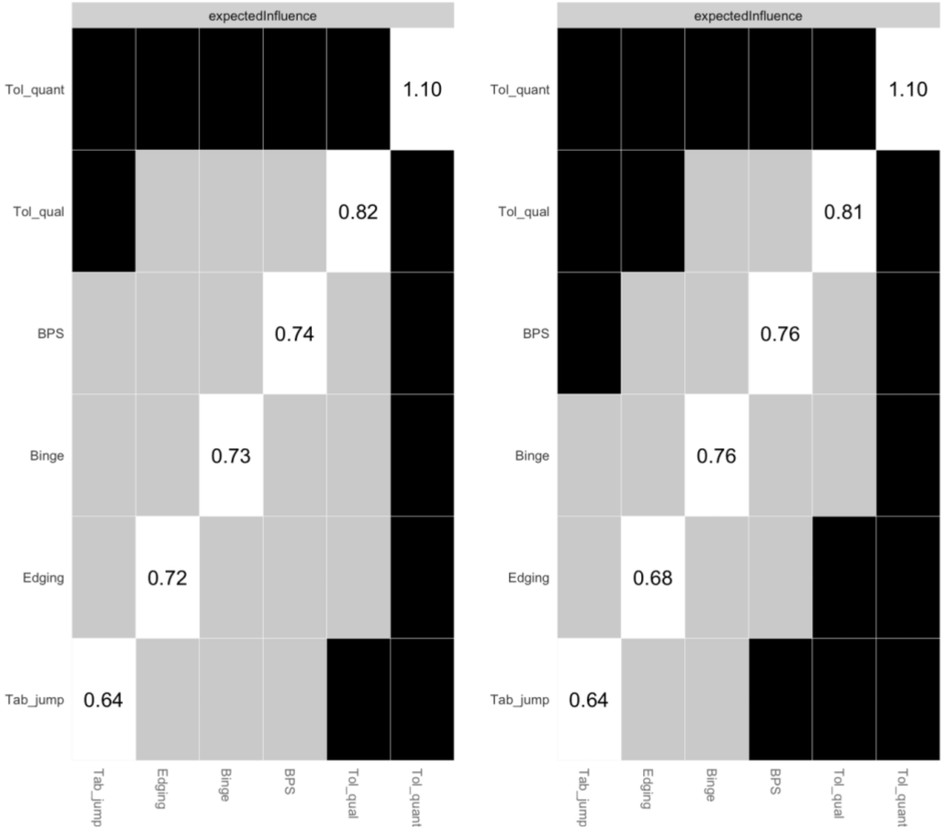

Bridge centrality

Model 1: BPS items modelled as individual nodes

Sample 1

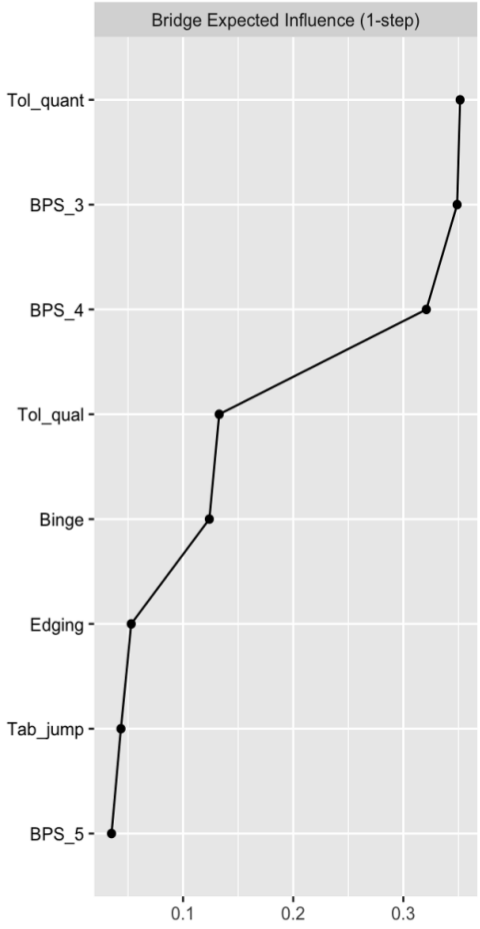

Sample 2

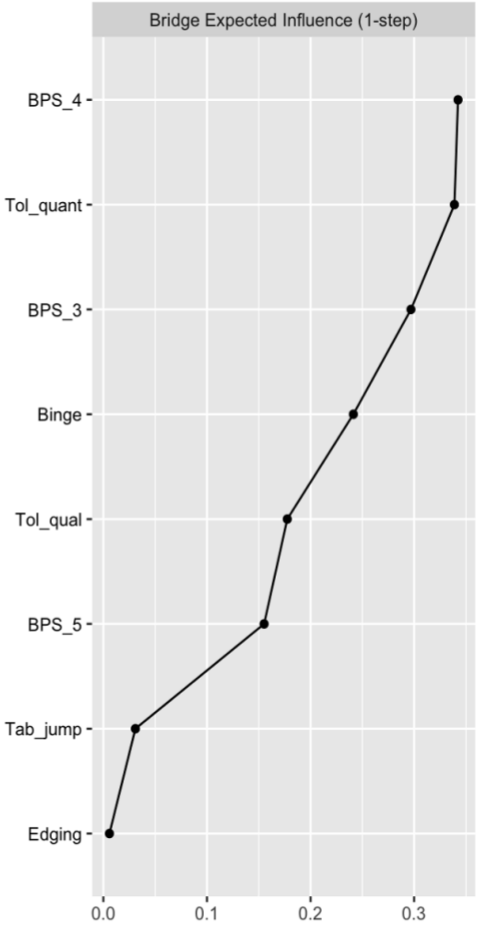

Sample 1

Sample 2

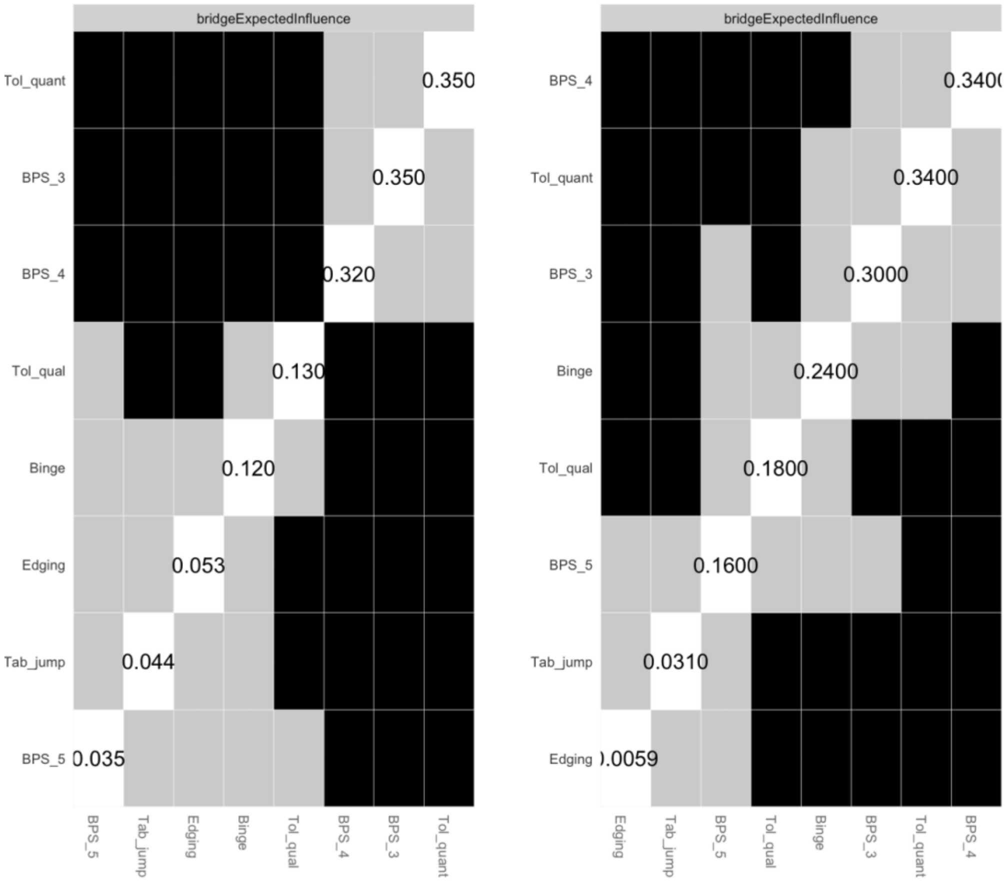
bridgeExpectedInfluence
Tol_quant
BPS_3
BPS_4
Tol_qual
Binge
Edging
Tab_jump
BPS_5
0.350
0.350
0.320
0.130
0.120
0.053
0.044
0.035
BPS_5
Tab_jump
Edging
Binge
Tol_qual
BPS_4
BPS_3
Tol_quant
bridgeExpectedInfluence
BPS_4
Tol_quant
BPS_3
Binge
Tol_qual
BPS_5
Tab_jump
Edging
0.3400
0.3000
0.2400
0.1800
0.1600
0.0310
Edging
Tab_jump
BPS_5
Tol_qual
Binge
BPS_3
Tol_quant
BPS_4

Network stability: correlation stability coefficients

Model 1: BPS items modelled as individual nodes

Expected influence: Correlation stability coefficient (expected influence): Sample 1= 0.75, Sample 2= 0.75

Sample 1 Sample 2

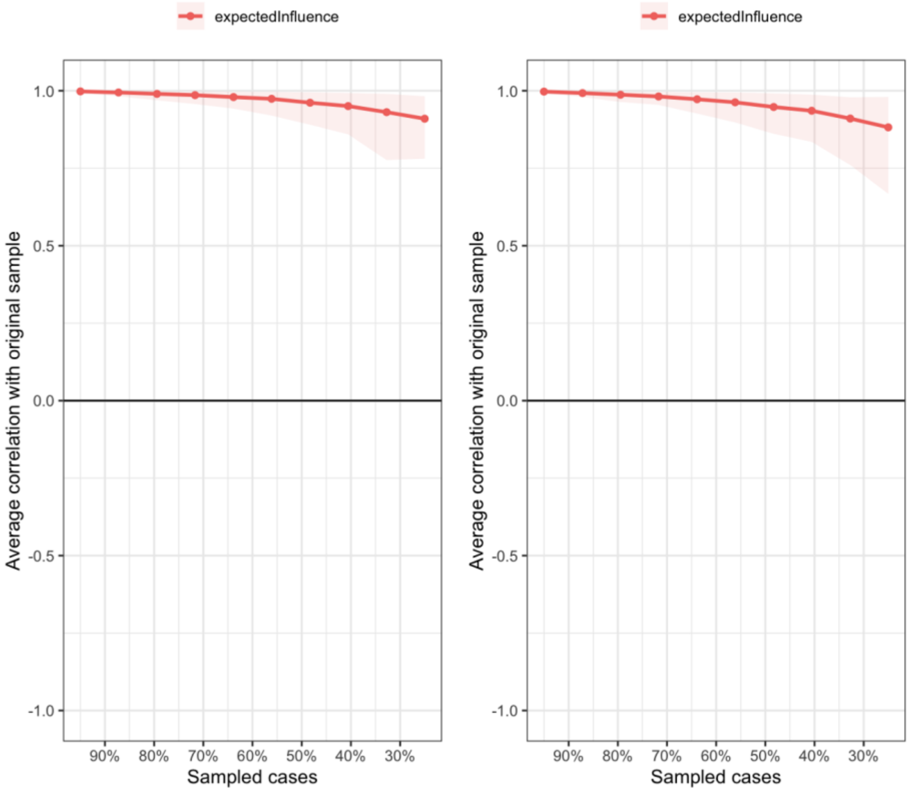

Bridge expected influence: Correlation stability coefficient (bridge expected influence): Sample 1= 0.75, Sample 2= 0.75

Sample 1 Sample 2

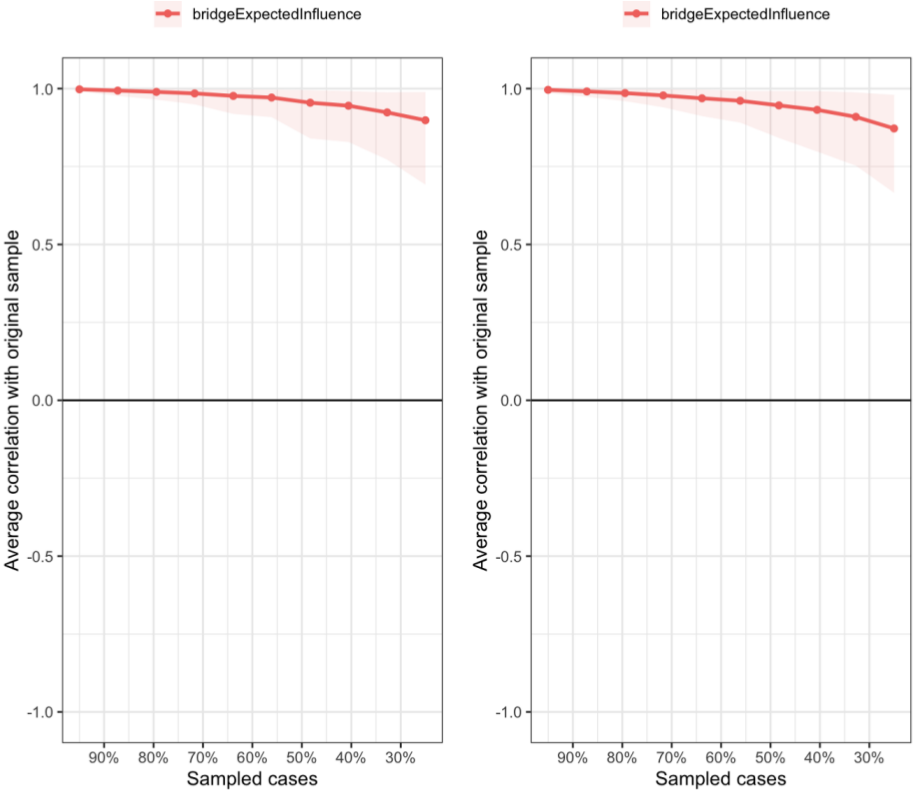

Model 2: BPS modelled as composite score

Expected influence: Correlation stability coefficient (expected influence): Sample 1= 0.75, Sample 2= 0.75.

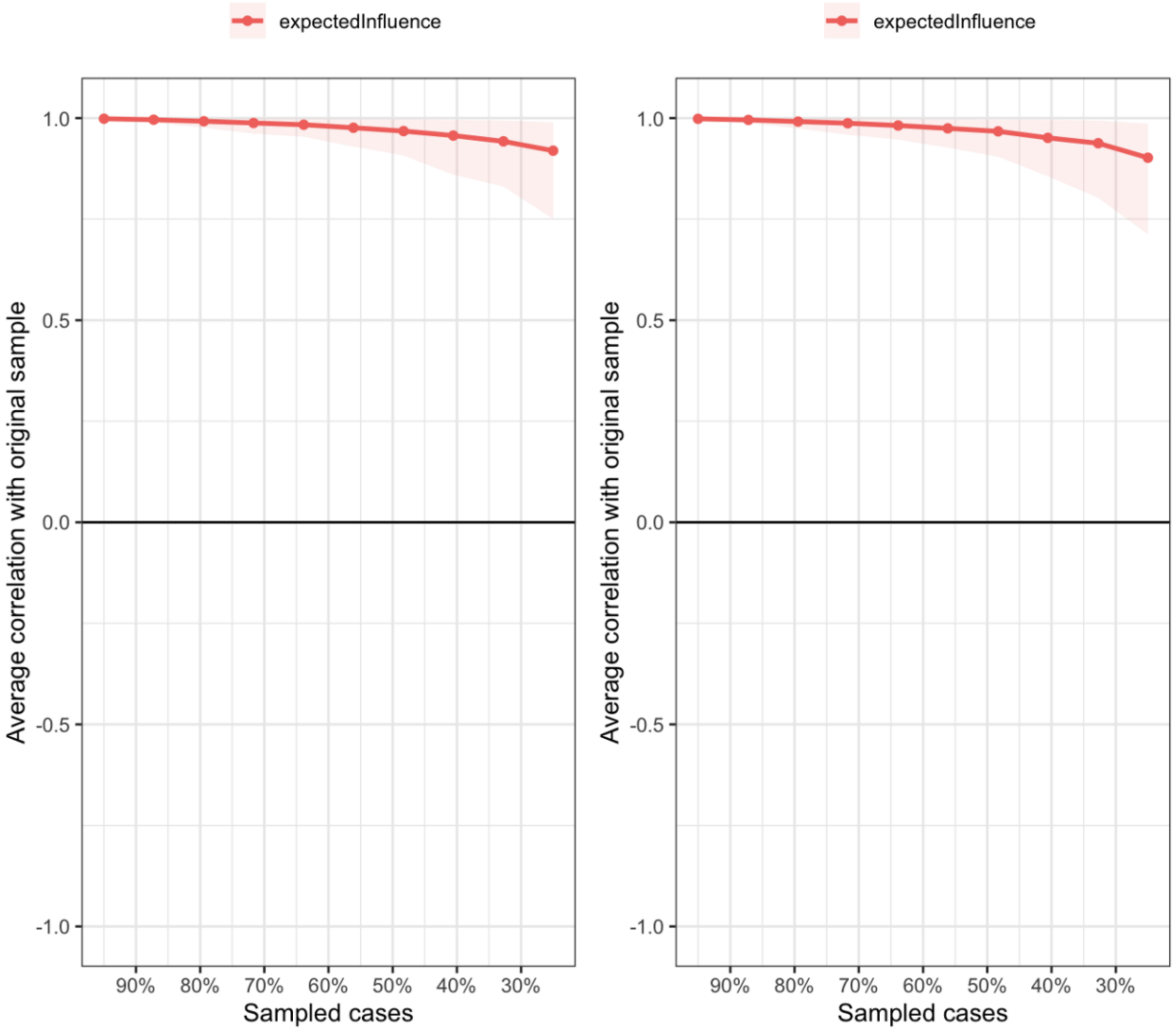

.

Sensitivity analysis 4 (SA4): ***Node redundancy*** (*Goldbricker*) and *community detection* (Walktrap algorithm) on the original networks.

Node redundancy with *Goldbricker* function

<u>Model 1</u>: BPS items modelled as separate nodes

Sample 1

```
  |==========================================================================================================| 100%
Suggested reductions: Less than 25 % of correlations are significantly different for the following pairs:
[1] "No suggested reductions"
  |==========================================================================================================| 100%
Suggested reductions: Less than 25 % of correlations are significantly different for the following pairs:
[1] "No suggested reductions"
```

Outcome: *No redundancy identified.*

Sample 2

```
  |==========================================================================================================| 100%
Suggested reductions: Less than 25 % of correlations are significantly different for the following pairs:
[1] "No suggested reductions"
  |==========================================================================================================| 100%
Suggested reductions: Less than 25 % of correlations are significantly different for the following pairs:
[1] "No suggested reductions"
```

Outcome: *No redundancy identified.*

<u>Model 2</u>: BPS modelled as composite score

Sample 1

```
  |==========================================================================================================| 100%
Suggested reductions: Less than 25 % of correlations are significantly different for the following pairs:
[1] "No suggested reductions"
  |==========================================================================================================| 100%
Suggested reductions: Less than 25 % of correlations are significantly different for the following pairs:
[1] "No suggested reductions"
```

Outcome: *No redundancy identified.*

Sample 2

```
  |==========================================================================================================| 100%
Suggested reductions: Less than 25 % of correlations are significantly different for the following pairs:
[1] "No suggested reductions"
  |==========================================================================================================| 100%
Suggested reductions: Less than 25 % of correlations are significantly different for the following pairs:
[1] "No suggested reductions"
```

Community detection with *Walktrap* algorithm

Model 1: BPS items modelled as separate nodes

Sample 1

```
Algorithm:  Walktrap

Number of communities:  3

V01 V02 V03 V04 V05 V06 V07 V08 V09 V10
  1   1   1   1   1   2   2   3   3   3
Algorithm:  Walktrap

Number of communities:  3

V01 V02 V03 V04 V05 V06 V07 V08 V09 V10
  1   1   1   1   1   2   2   3   3   3
```

Sample 2

```
Algorithm:  Walktrap

Number of communities:  3

V01 V02 V03 V04 V05 V06 V07 V08 V09 V10
  1   1   1   1   1   2   2   3   3   3
Algorithm:  Walktrap

Number of communities:  3

V01 V02 V03 V04 V05 V06 V07 V08 V09 V10
  1   1   1   1   1   2   2   3   3   3
```

Model 2: BPS modelled as composite score

Sample 1

```
Algorithm:  Walktrap

Number of communities:  2

V1 V2 V3 V4 V5 V6
 1  1  1  2  2  2
```

```
Algorithm:  Walktrap

Number of communities:  2

V1 V2 V3 V4 V5 V6
 1  1  1  2  2  2
```

Sample 2

```
Algorithm:  Walktrap

Number of communities:  2

V1 V2 V3 V4 V5 V6
 1  1  1  2  2  2
```

```
Algorithm:  Walktrap

Number of communities:  2

V1 V2 V3 V4 V5 V6
 1  1  1  2  2  2
```

Sensitivity analysis 5 (SA5): Comparing networks ***after removing scores of BPS=0***

NB: The proportion of individuals with a BPS score of 0 is as follows: Sample 1: 433/1356 (32%); Sample 2: 266/944 (28%).

Estimated networks

Model 1: BPS items modelled as separate nodes

Sample 1

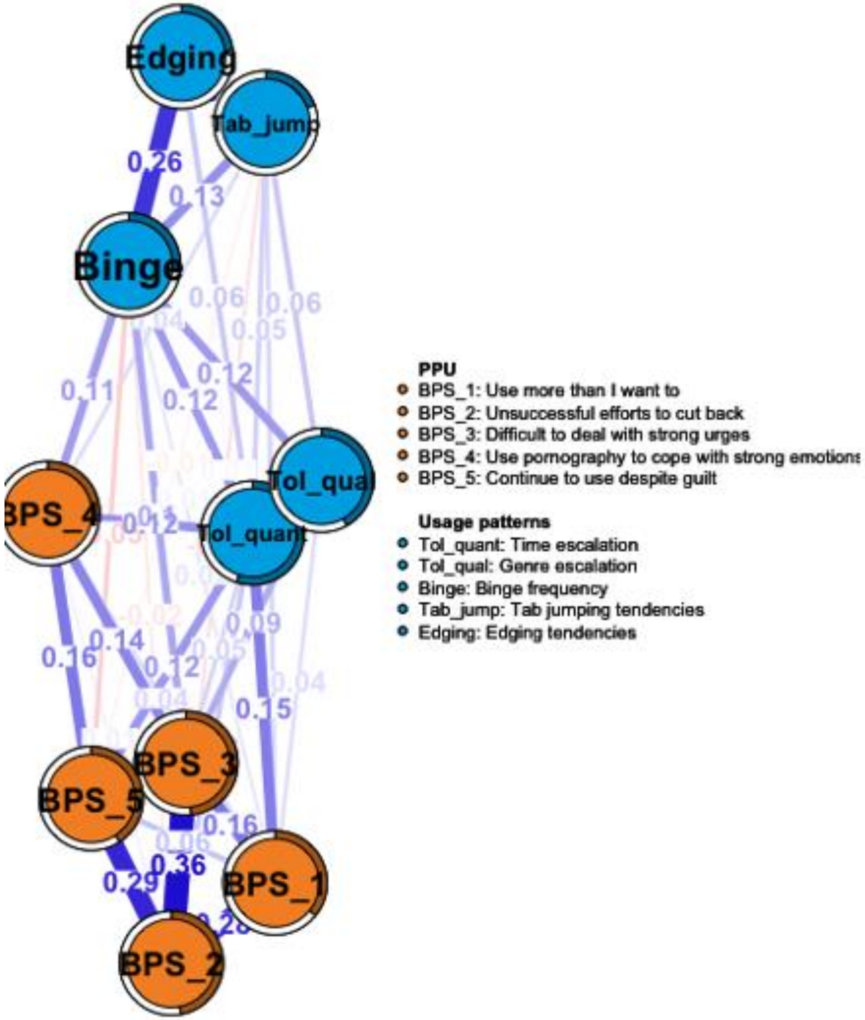

Sample 2

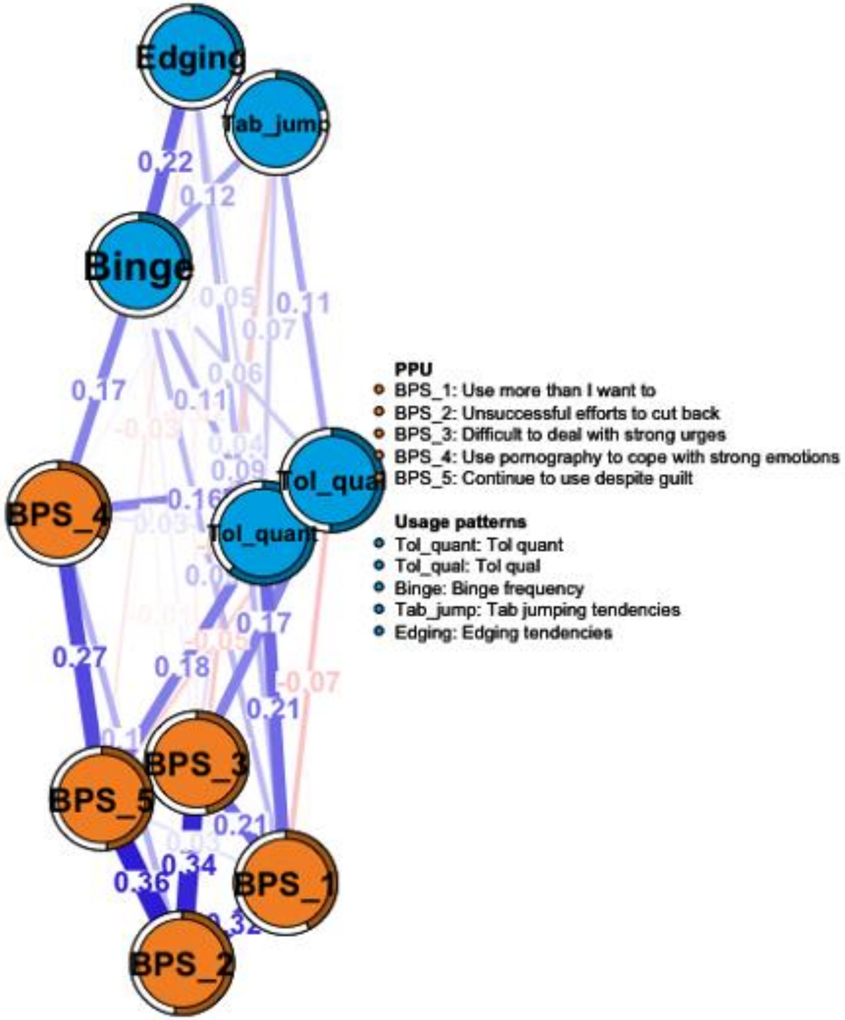

Model 2: BPS modelled as composite score

Sample 1

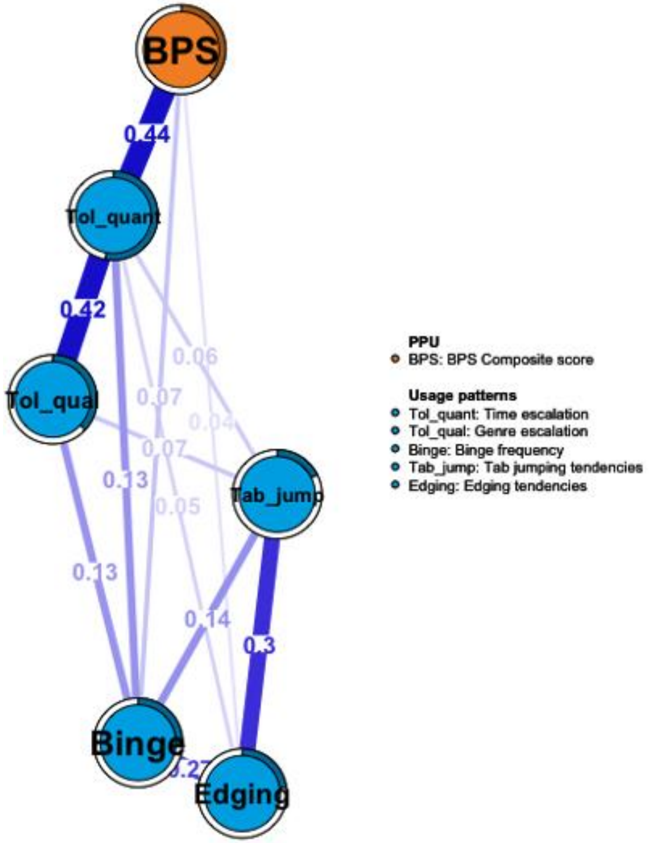

Sample 2

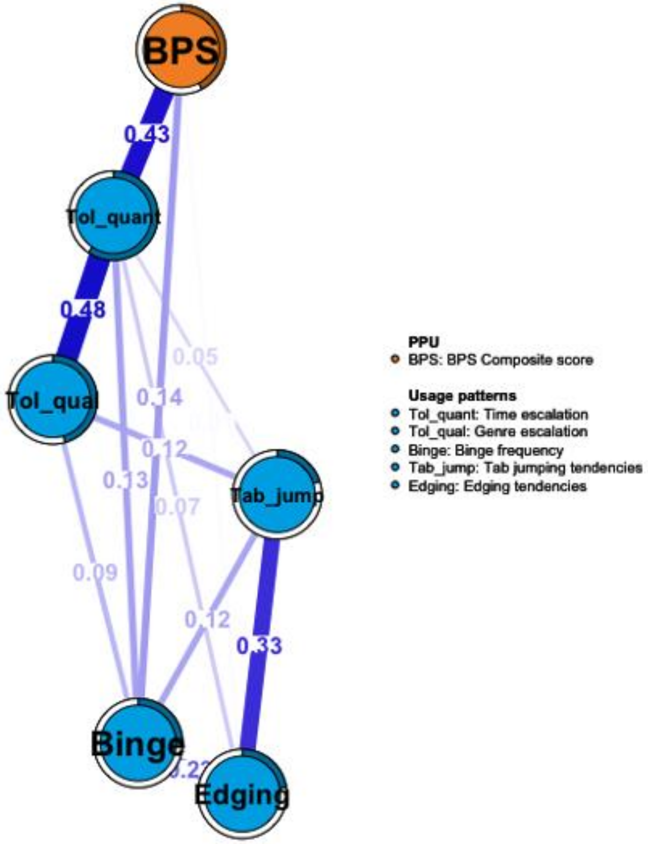

NCT: comparison with original (full) sample

Model 1: BPS items modelled as separate nodes

Sample 1

```
NETWORK INVARIANCE TEST
Test statistic M:
0.1226242
p-value 0.6637345

GLOBAL STRENGTH INVARIANCE TEST
Global strength per group:  4.874233 4.326052
Test statistic S:  0.5481811
p-value 0.08156737

EDGE INVARIANCE TEST
       Var1      Var2    p-value Test statistic E
11     BPS_1     BPS_2 0.67732907       0.03007560
21     BPS_1     BPS_3 0.55337865       0.04178350
22     BPS_2     BPS_3 0.88084766       0.01085134
31     BPS_1     BPS_4 0.07117153       0.12262419
32     BPS_2     BPS_4 0.74690124       0.01329482
33     BPS_3     BPS_4 0.76729308       0.01855419
41     BPS_1     BPS_5 0.33226709       0.06773409
42     BPS_2     BPS_5 0.89924030       0.00797759
43     BPS_3     BPS_5 0.70611755       0.02326879
44     BPS_4     BPS_5 0.32706917       0.05907858
51     BPS_1 Tol_quant 0.42862855       0.04264579
52     BPS_2 Tol_quant 0.93402639       0.00447490
53     BPS_3 Tol_quant 0.55177929       0.03406256
54     BPS_4 Tol_quant 0.28308677       0.05058609
55     BPS_5 Tol_quant 0.85325870       0.00943776
61     BPS_1  Tol_qual 0.84486206       0.01058293
62     BPS_2  Tol_qual 1.00000000       0.00000000
63     BPS_3  Tol_qual 0.86005598       0.01035430
64     BPS_4  Tol_qual 0.82287085       0.01304553
65     BPS_5  Tol_qual 0.66413435       0.01289640
66 Tol_quant  Tol_qual 0.85885646       0.00701054
71     BPS_1     Binge 0.95321871       0.00430455
72     BPS_2     Binge 0.62814874       0.03772178
73     BPS_3     Binge 0.70891643       0.02478543
74     BPS_4     Binge 0.98080768       0.00148396
75     BPS_5     Binge 0.52618952       0.04130405
76 Tol_quant     Binge 0.91083567       0.00519132
77  Tol_qual     Binge 0.73970412       0.01657551
81     BPS_1  Tab_jump 0.62534986       0.02953863
82     BPS_2  Tab_jump 0.65773691       0.03113671
83     BPS_3  Tab_jump 0.75529788       0.01644208
84     BPS_4  Tab_jump 0.75449820       0.01809398
85     BPS_5  Tab_jump 0.73930428       0.02262602
86 Tol_quant  Tab_jump 0.97840864       0.00100171
87  Tol_qual  Tab_jump 0.77209116       0.01547736
88     Binge  Tab_jump 0.85805678       0.00924409
91     BPS_1    Edging 0.68492603       0.00689896
92     BPS_2    Edging 0.67932827       0.01642769
93     BPS_3    Edging 1.00000000       0.00000000
94     BPS_4    Edging 0.97161136       0.00251787
95     BPS_5    Edging 0.67213115       0.02200779
96 Tol_quant    Edging 0.70491803       0.01666886
97  Tol_qual    Edging 0.48620552       0.01251881
98     Binge    Edging 0.99160336       0.00041723
99  Tab_jump    Edging 0.43542583       0.03789092

CENTRALITY INVARIANCE TEST p-value
          expectedInfluence
BPS_1           0.004398241
BPS_2           0.703318673
BPS_3           0.229508197
BPS_4           0.029188325
BPS_5           0.360655738
Tol_quant       0.004398241
Tol_qual        0.348660536
Binge           0.416233507
Tab_jump        0.387844862
Edging          0.286685326
```

[illegible]

Sample 2

```
NETWORK INVARIANCE TEST
Test statistic M:
0.08819186
p-value 0.9968013

GLOBAL STRENGTH INVARIANCE TEST
Global strength per group:  4.703865 4.773766
Test statistic S:  0.06990088
p-value 0.9048381

EDGE INVARIANCE TEST
       Var1      Var2   p-value Test statistic E
11     BPS_1     BPS_2 0.8764494      0.01169440
21     BPS_1     BPS_3 0.2814874      0.08291895
22     BPS_2     BPS_3 0.8212715      0.01721580
31     BPS_1     BPS_4 0.2566973      0.08819186
32     BPS_2     BPS_4 0.6805278      0.03379200
33     BPS_3     BPS_4 0.5861655      0.03909957
41     BPS_1     BPS_5 0.5029988      0.06217557
42     BPS_2     BPS_5 0.8944422      0.01013613
43     BPS_3     BPS_5 0.7964814      0.02112468
44     BPS_4     BPS_5 0.5629748      0.04109502
51     BPS_1 Tol_quant 0.4526190      0.04091232
52     BPS_2 Tol_quant 0.8996401      0.00688255
53     BPS_3 Tol_quant 1.0000000      0.00000000
54     BPS_4 Tol_quant 0.8696521      0.01121725
55     BPS_5 Tol_quant 0.4342263      0.04216829
61     BPS_1  Tol_qual 0.7932827      0.02465260
62     BPS_2  Tol_qual 1.0000000      0.00000000
63     BPS_3  Tol_qual 0.6929228      0.02766199
64     BPS_4  Tol_qual 0.7552979      0.02052218
65     BPS_5  Tol_qual 0.6577369      0.03165054
66 Tol_quant  Tol_qual 0.2662935      0.05352695
71     BPS_1     Binge 0.7836865      0.01826699
72     BPS_2     Binge 0.5933627      0.01100501
73     BPS_3     Binge 0.7636945      0.01651211
74     BPS_4     Binge 0.7596961      0.01945781
75     BPS_5     Binge 1.0000000      0.00000000
76 Tol_quant     Binge 0.7025190      0.02131062
77  Tol_qual     Binge 0.5217913      0.04145422
81     BPS_1  Tab_jump 1.0000000      0.00000000
82     BPS_2  Tab_jump 0.8440624      0.01806622
83     BPS_3  Tab_jump 0.9928029      0.00095900
84     BPS_4  Tab_jump 0.9060376      0.00815265
85     BPS_5  Tab_jump 1.0000000      0.00000000
86 Tol_quant  Tab_jump 0.7932827      0.01460550
87  Tol_qual  Tab_jump 0.8112755      0.01472045
88     Binge  Tab_jump 0.6997201      0.02408653
91     BPS_1    Edging 0.8780488      0.00986629
92     BPS_2    Edging 1.0000000      0.00000000
93     BPS_3    Edging 0.6453419      0.02050305
94     BPS_4    Edging 1.0000000      0.00000000
95     BPS_5    Edging 0.9540184      0.00204804
96 Tol_quant    Edging 0.9620152      0.00183547
97  Tol_qual    Edging 0.5881647      0.01431455
98     Binge    Edging 0.6757297      0.02802034
99  Tab_jump    Edging 0.9144342      0.00597214

CENTRALITY INVARIANCE TEST p-value
          expectedInfluence
BPS_1            0.06597361
BPS_2            0.65933627
BPS_3            0.31067573
BPS_4            0.25869652
BPS_5            0.25989604
Tol_quant        0.02199120
Tol_qual         0.64534186
Binge            0.81047581
Tab_jump         0.95401839
Edging           0.74250300
```

Model 2: BPS modelled as composite score

Sample 1

```
NETWORK INVARIANCE TEST
Test statistic M:
0.04524831
p-value 0.9872051

GLOBAL STRENGTH INVARIANCE TEST
Global strength per group:  2.354794 2.209553
Test statistic S:  0.1452403
p-value 0.02039184

EDGE INVARIANCE TEST
        Var1      Var2    p-value Test statistic E
7        BPS Tol_quant 0.45981607       0.02496623
13       BPS  Tol_qual 0.05117953       0.04524831
14 Tol_quant  Tol_qual 0.44662135       0.02895458
19       BPS     Binge 0.84286285       0.00911191
20 Tol_quant     Binge 0.79608157       0.01105477
21  Tol_qual     Binge 0.67333067       0.01959794
25       BPS  Tab_jump 0.68252699       0.01962314
26 Tol_quant  Tab_jump 0.64854058       0.02087400
27  Tol_qual  Tab_jump 0.42582967       0.03851928
28     Binge  Tab_jump 0.99120352       0.00075472
31       BPS    Edging 0.85045982       0.00901215
32 Tol_quant    Edging 0.99560176       0.00020177
33  Tol_qual    Edging 0.59896042       0.00506950
34     Binge    Edging 0.98360656       0.00103417
35  Tab_jump    Edging 0.51459416       0.03249506

CENTRALITY INVARIANCE TEST p-value
          expectedInfluence
BPS              0.11715314
Tol_quant        0.71331467
Tol_qual         0.05317873
Binge            0.57696921
Tab_jump         0.28428629
Edging           0.43182727
```

Sample 2

```
NETWORK INVARIANCE TEST
Test statistic M:
0.03503044
p-value 1

GLOBAL STRENGTH INVARIANCE TEST
Global strength per group:  2.362131 2.278978
Test statistic S:  0.08315292
p-value 0.3314674

EDGE INVARIANCE TEST
        Var1      Var2   p-value Test statistic E
7        BPS Tol_quant 0.4418233       0.03100793
13       BPS  Tol_qual 0.4862055       0.01839327
14 Tol_quant  Tol_qual 0.8596561       0.00738250
19       BPS     Binge 0.8916433       0.00722654
20 Tol_quant     Binge 0.9232307       0.00508199
21  Tol_qual     Binge 0.6373451       0.02590990
25       BPS  Tab_jump 1.0000000       0.00000000
26 Tol_quant  Tab_jump 0.9876050       0.00085859
27  Tol_qual  Tab_jump 0.6865254       0.02161754
28     Binge  Tab_jump 0.8468613       0.01241564
31       BPS    Edging 0.7337065       0.01754212
32 Tol_quant    Edging 0.9540184       0.00309550
33  Tol_qual    Edging 1.0000000       0.00000000
34     Binge    Edging 0.5885646       0.03503044
35  Tab_jump    Edging 0.9336265       0.00480196

CENTRALITY INVARIANCE TEST p-value
          expectedInfluence
BPS               0.2163135
Tol_quant         0.6961216
Tol_qual          0.9048381
Binge             0.9068373
Tab_jump          0.9348261
Edging            0.5329868
```

Node centrality

Model 1: BPS items modelled as individual nodes

Sample 1 Sample 2

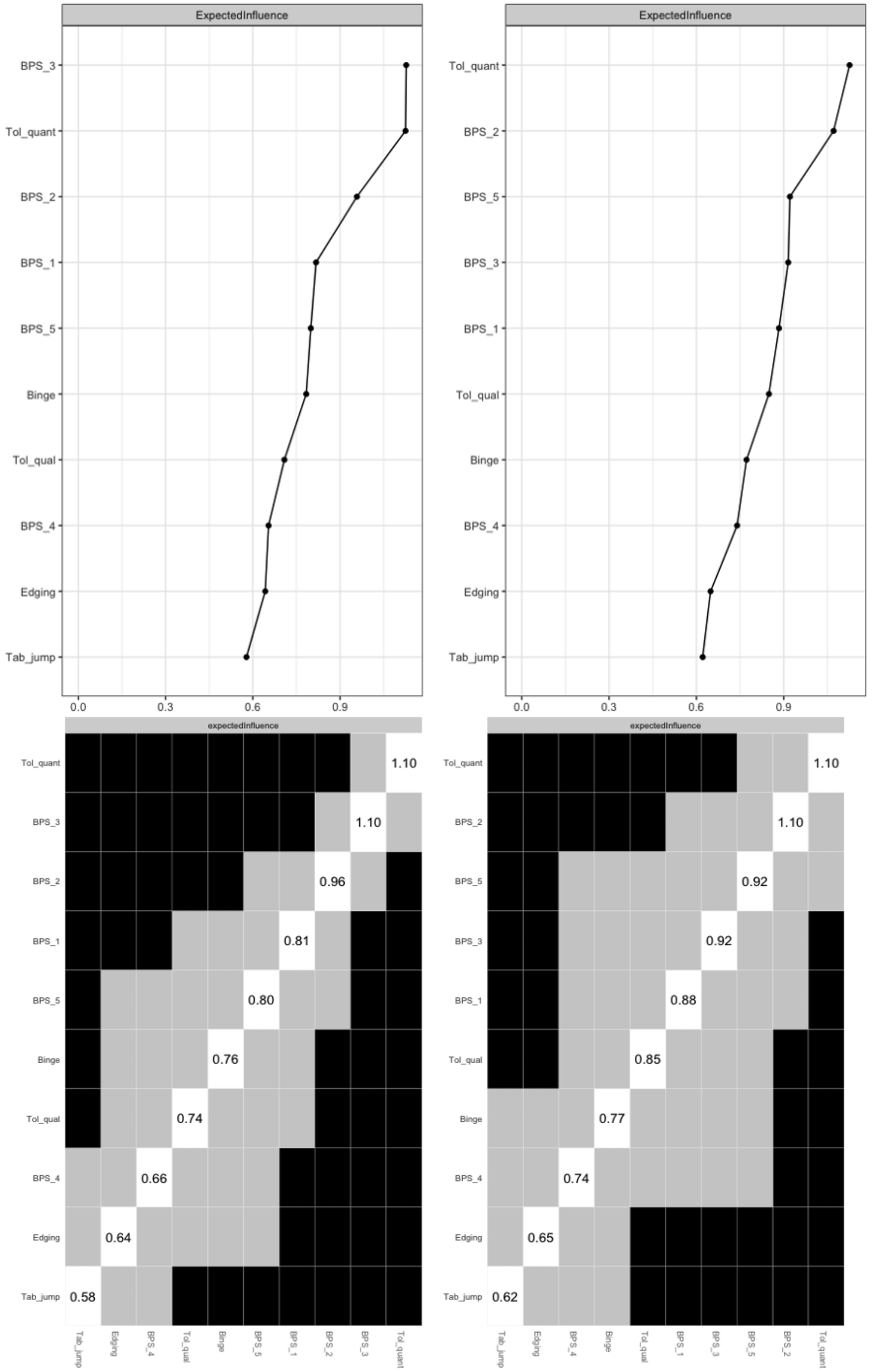

Model 2: BPS modelled as composite score

Sample 1 Sample 2

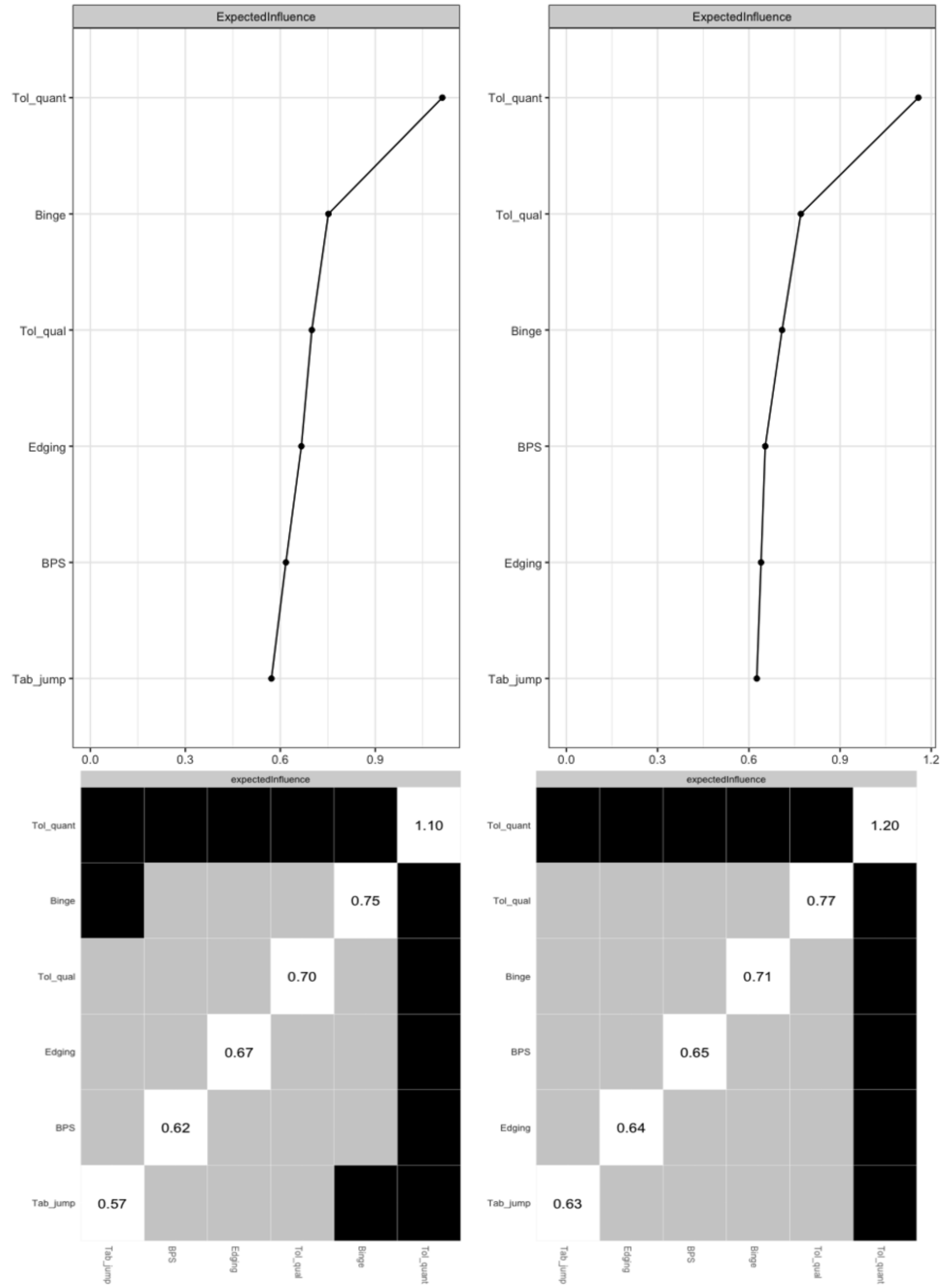

Bridge centrality

Model 1: BPS items modelled as individual nodes

Sample 1 Sample 2

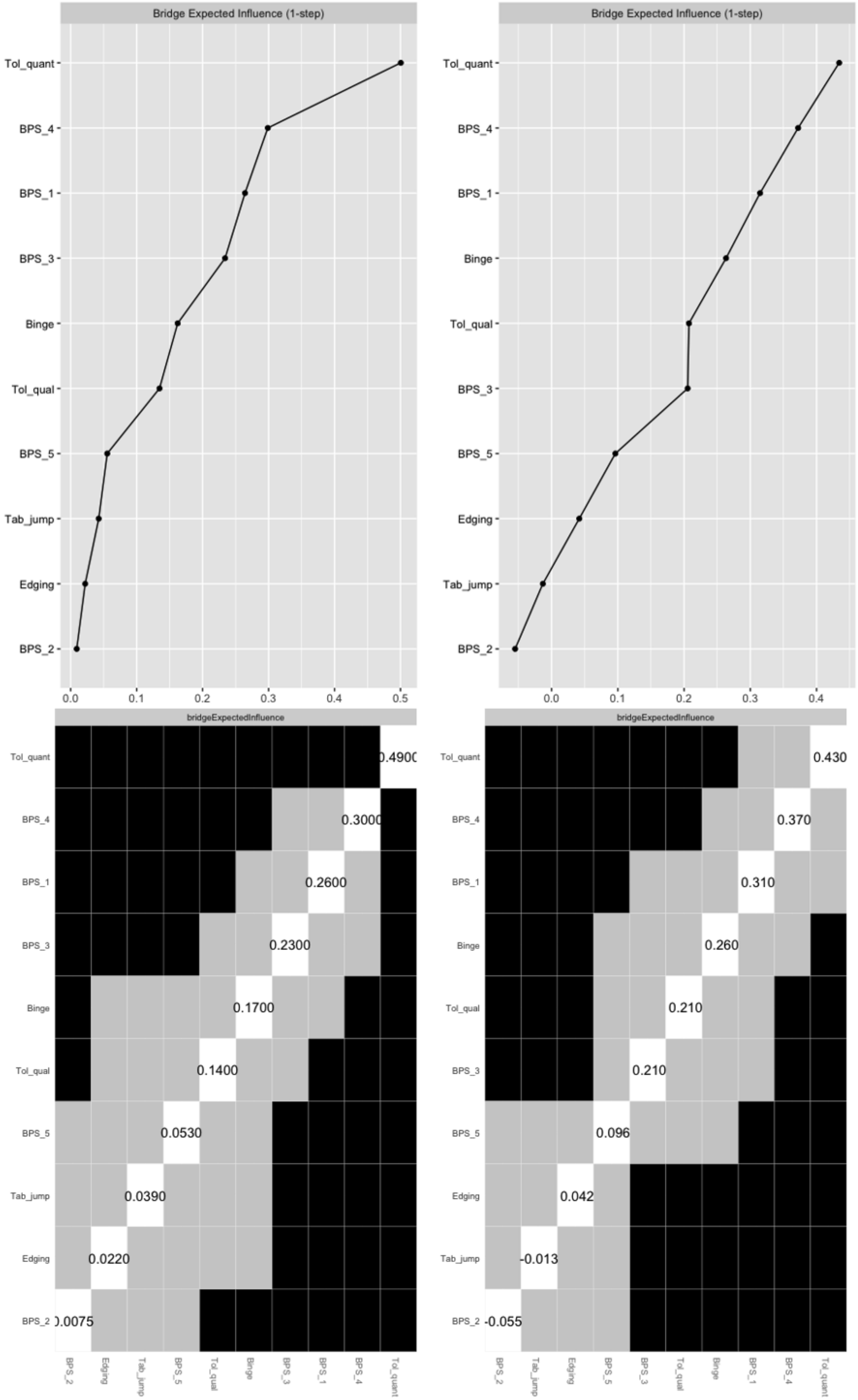

Network stability: correlation stability coefficients

Model 1: BPS items modelled as individual nodes

Expected influence: Correlation stability coefficient (expected influence): Sample 1= 0.75, Sample 2= 0.67

Sample 1 Sample 2

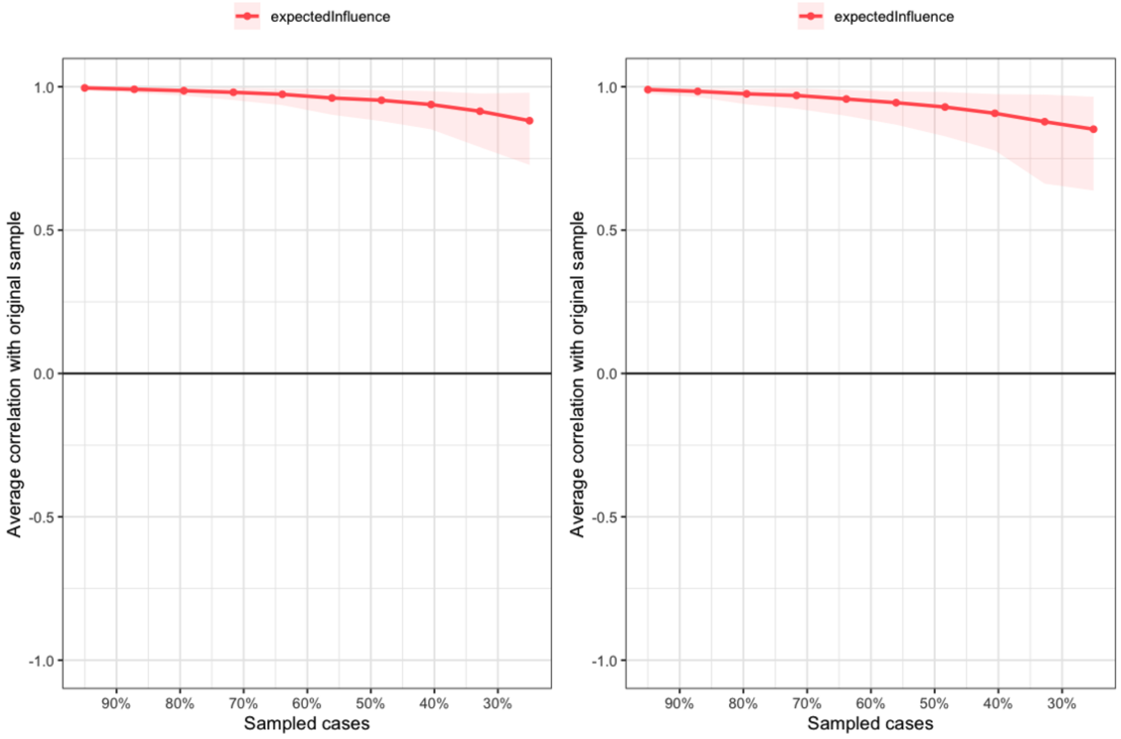

Bridge expected influence: Correlation stability coefficient (bridge expected influence): Sample 1= 0.75, Sample 2= 0.67

Sample 1 Sample 2

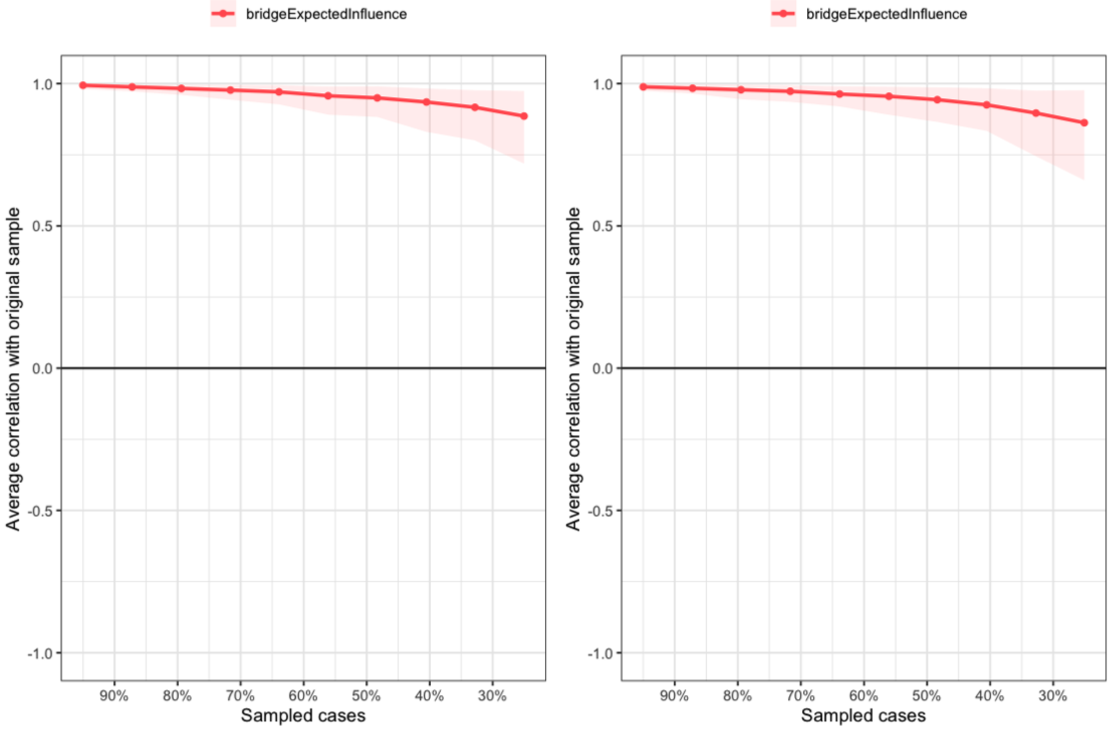

Model 2: BPS modelled as composite score

Expected influence: Correlation stability coefficient (expected influence): Sample 1= 0.75, Sample 2= 0. 75.

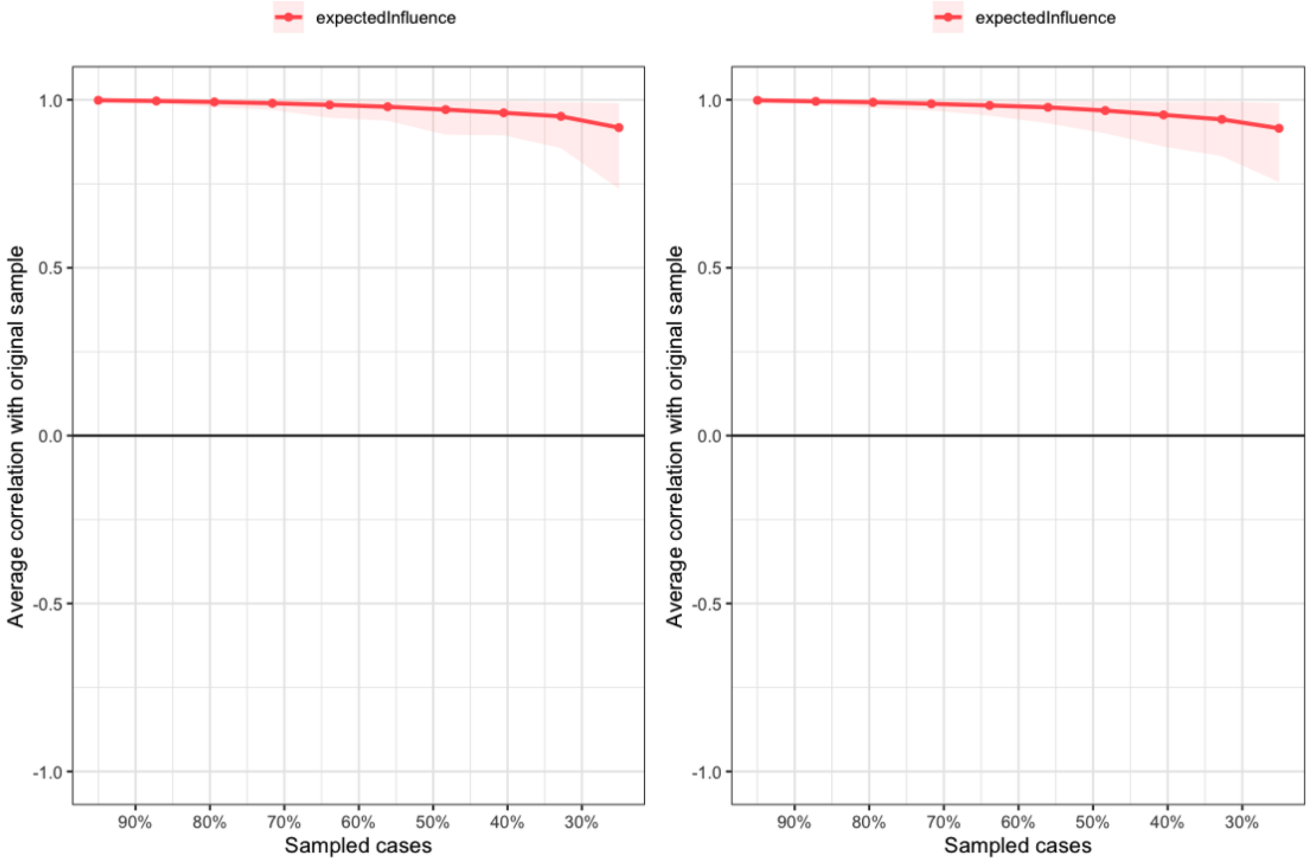

Sensitivity analysis 6 (SA6) Comparing networks for individuals ***below vs above BPS cut-off score (BPS≥4).***

NB: The proportion of individuals who met the identified cut-off for possible PPU is as follows: Sample 1: 877/1356 (35%); Sample 2: 627/944 (34%).

Estimated networks

Model 1: BPS items modelled as separate nodes

Below cutoff

Sample 1

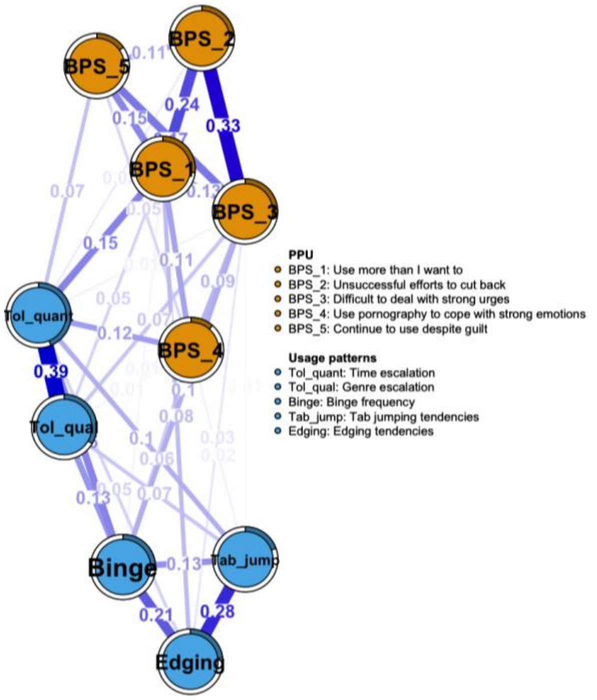

Sample 2

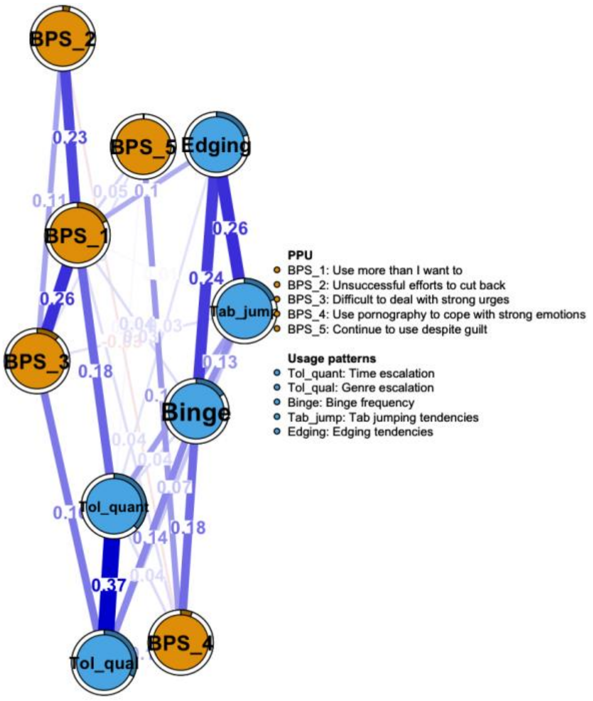

Above cutoff

Sample 1

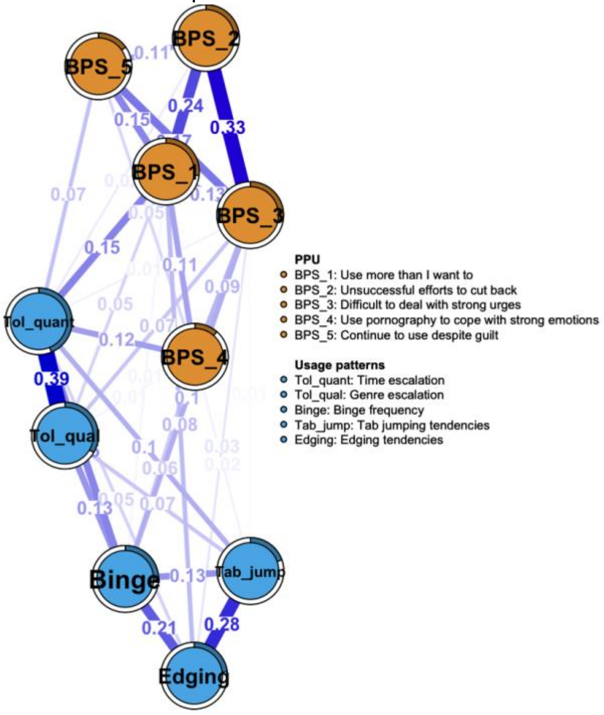

Sample 2

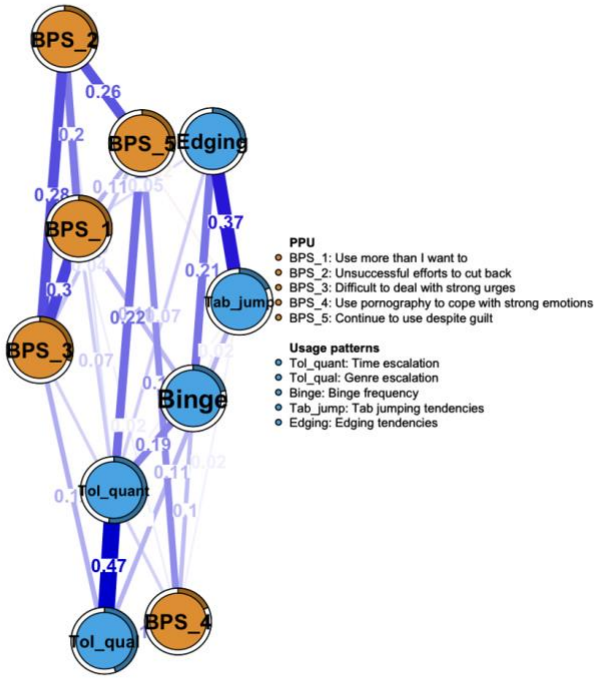

Model 2: BPS modelled as composite score

Below cutoff

Sample 1

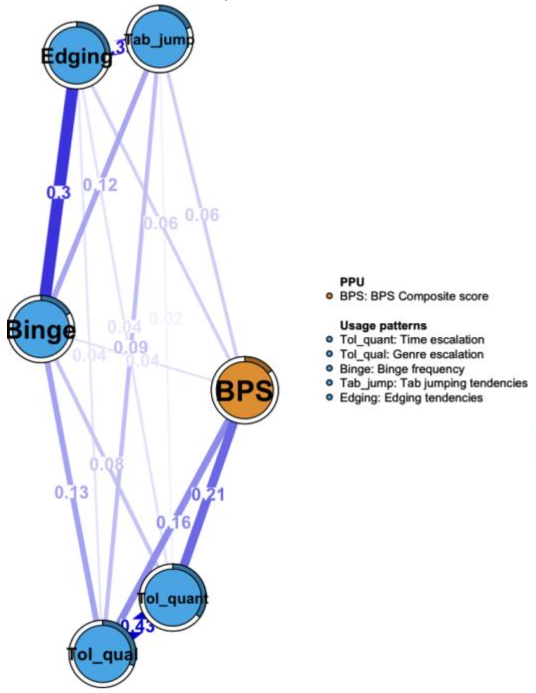

Sample 2

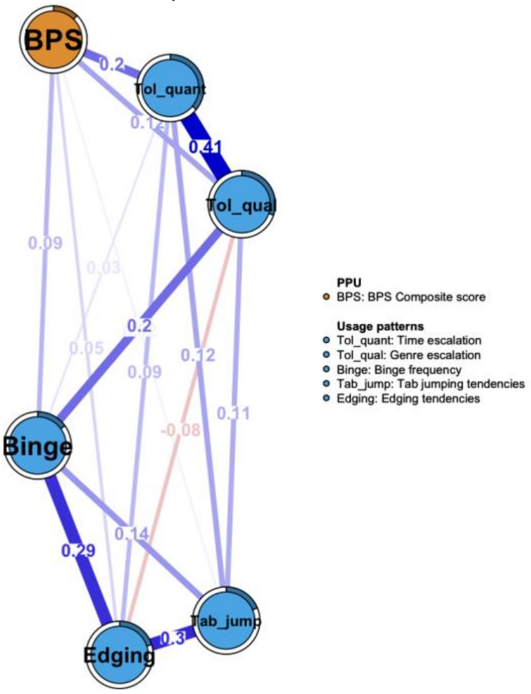

Above cutoff

Sample 1

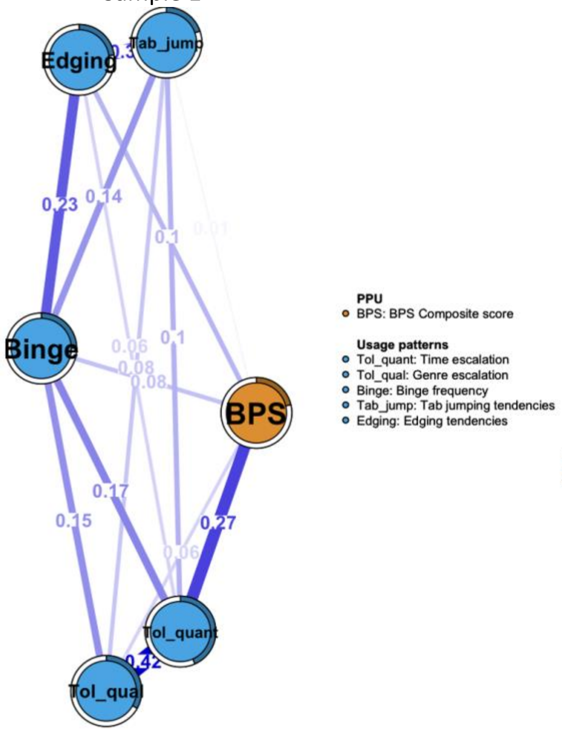

Sample 2

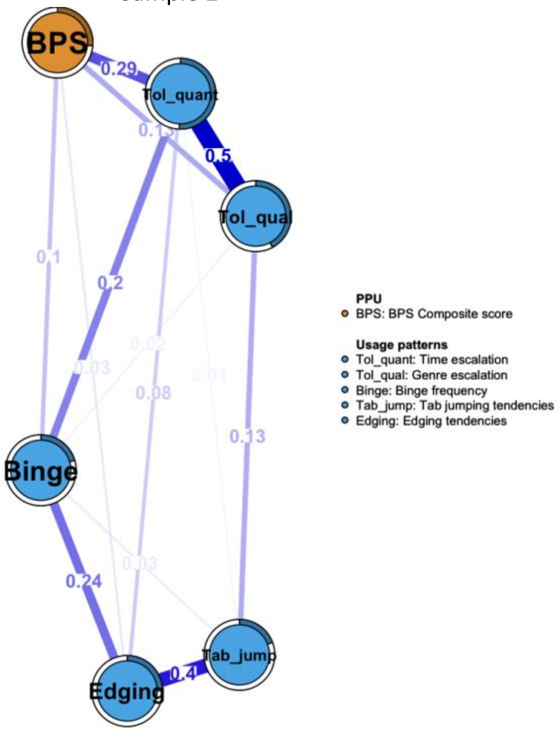

NCT: Individuals below vs above BPS cut-off score for each sample

Model 1: BPS items modelled as individual nodes

Sample 1

```
NETWORK INVARIANCE TEST
Test statistic M:
0.2646649
p-value 0.04918033

GLOBAL STRENGTH INVARIANCE TEST
Global strength per group:  3.717809 4.387982
Test statistic S:  0.6701728
p-value 0.09916034

EDGE INVARIANCE TEST
        Var1      Var2     p-value Test statistic E
11     BPS_1     BPS_2 0.361855258       0.08986388
21     BPS_1     BPS_3 0.256297481       0.11119596
22     BPS_2     BPS_3 0.005597761       0.26466495
31     BPS_1     BPS_4 0.459016393       0.06796235
32     BPS_2     BPS_4 0.003998401       0.20568278
33     BPS_3     BPS_4 0.339064374       0.08818349
41     BPS_1     BPS_5 0.208716513       0.12224588
42     BPS_2     BPS_5 0.150339864       0.13470196
43     BPS_3     BPS_5 0.220711715       0.11869749
44     BPS_4     BPS_5 0.167133147       0.12083727
51     BPS_1 Tol_quant 0.234306277       0.08608131
52     BPS_2 Tol_quant 0.621351459       0.03605601
53     BPS_3 Tol_quant 0.736105558       0.02464117
54     BPS_4 Tol_quant 0.341463415       0.06064807
55     BPS_5 Tol_quant 0.564974010       0.03862975
61     BPS_1  Tol_qual 0.933226709       0.00715397
62     BPS_2  Tol_qual 0.115153938       0.05592852
63     BPS_3  Tol_qual 0.236705318       0.09100390
64     BPS_4  Tol_qual 0.549380248       0.04320535
65     BPS_5  Tol_qual 1.000000000       0.00000000
66 Tol_quant  Tol_qual 0.740103958       0.01790722
71     BPS_1     Binge 0.863254698       0.01192887
72     BPS_2     Binge 0.907636945       0.01373269
73     BPS_3     Binge 0.828068772       0.02039885
74     BPS_4     Binge 0.359856058       0.07432770
75     BPS_5     Binge 0.166333467       0.11937540
76 Tol_quant     Binge 0.228708517       0.06909896
77  Tol_qual     Binge 0.756097561       0.02405588
81     BPS_1  Tab_jump 0.147141144       0.10818389
82     BPS_2  Tab_jump 0.343062775       0.07808041
83     BPS_3  Tab_jump 0.893242703       0.00300221
84     BPS_4  Tab_jump 0.574970012       0.04213989
85     BPS_5  Tab_jump 1.000000000       0.00000000
86 Tol_quant  Tab_jump 0.173930428       0.08108611
87  Tol_qual  Tab_jump 0.874850060       0.01106364
88     Binge  Tab_jump 0.663334666       0.03140261
91     BPS_1    Edging 0.053978409       0.08220660
92     BPS_2    Edging 0.125949620       0.06266654
93     BPS_3    Edging 0.251899240       0.02964909
94     BPS_4    Edging 0.074370252       0.08401791
95     BPS_5    Edging 0.740503798       0.01292831
96 Tol_quant    Edging 0.682127149       0.02196350
97  Tol_qual    Edging 0.551379448       0.03055516
98     Binge    Edging 0.350259896       0.06375019
99  Tab_jump    Edging 0.406637345       0.05420281

CENTRALITY INVARIANCE TEST p-value
          expectedInfluence
BPS_1           0.556577369
BPS_2           0.141143543
BPS_3           0.013194722
BPS_4           0.465413834
BPS_5           0.662534986
Tol_quant       0.005197921
Tol_qual        0.007996801
Binge           0.105957617
Tab_jump        0.794482207
Edging          0.023590564
```

Sample 2

```
NETWORK INVARIANCE TEST
Test statistic M:
0.2642896
p-value 0.1403439

GLOBAL STRENGTH INVARIANCE TEST
Global strength per group:  3.909677 3.110038
Test statistic S:  0.799639
p-value 0.1479408

EDGE INVARIANCE TEST
        Var1      Var2      p-value Test statistic E
11     BPS_1     BPS_2 0.8384646142       0.02498540
21     BPS_1     BPS_3 0.7301079568       0.03564071
22     BPS_2     BPS_3 0.1291483407       0.16978828
31     BPS_1     BPS_4 0.9048380648       0.01335394
32     BPS_2     BPS_4 0.8060775690       0.02990702
33     BPS_3     BPS_4 0.5285885646       0.06615072
41     BPS_1     BPS_5 0.6129548181       0.06738716
42     BPS_2     BPS_5 0.0143942423       0.26428962
43     BPS_3     BPS_5 0.6237504998       0.05251582
44     BPS_4     BPS_5 0.6117552979       0.05259524
51     BPS_1 Tol_quant 0.1299480208       0.10914593
52     BPS_2 Tol_quant 0.3406637345       0.04298051
53     BPS_3 Tol_quant 0.7904838065       0.00050522
54     BPS_4 Tol_quant 0.5477808876       0.03981802
55     BPS_5 Tol_quant 0.0003998401       0.21667909
61     BPS_1  Tol_qual 1.0000000000       0.00000000
62     BPS_2  Tol_qual 1.0000000000       0.00000000
63     BPS_3  Tol_qual 0.6477409036       0.04015373
64     BPS_4  Tol_qual 0.4034386246       0.07241596
65     BPS_5  Tol_qual 1.0000000000       0.00000000
66 Tol_quant  Tol_qual 0.1191523391       0.10067718
71     BPS_1     Binge 0.4666133547       0.06681183
72     BPS_2     Binge 1.0000000000       0.00000000
73     BPS_3     Binge 1.0000000000       0.00000000
74     BPS_4     Binge 0.3830467813       0.08040720
75     BPS_5     Binge 1.0000000000       0.00000000
76 Tol_quant     Binge 0.0291883247       0.14769254
77  Tol_qual     Binge 0.1111555378       0.13983174
81     BPS_1  Tab_jump 0.6969212315       0.00595773
82     BPS_2  Tab_jump 0.8388644542       0.02054907
83     BPS_3  Tab_jump 0.6933226709       0.03396368
84     BPS_4  Tab_jump 0.7688924430       0.02001405
85     BPS_5  Tab_jump 1.0000000000       0.00000000
86 Tol_quant  Tab_jump 0.1691323471       0.09542931
87  Tol_qual  Tab_jump 0.6029588165       0.04394821
88     Binge  Tab_jump 0.2215113954       0.10699405
91     BPS_1    Edging 0.5597760896       0.04688878
92     BPS_2    Edging 1.0000000000       0.00000000
93     BPS_3    Edging 1.0000000000       0.00000000
94     BPS_4    Edging 1.0000000000       0.00000000
95     BPS_5    Edging 0.7740903639       0.00660083
96 Tol_quant    Edging 0.5641743303       0.03965041
97  Tol_qual    Edging 1.0000000000       0.00000000
98     Binge    Edging 0.7337065174       0.02960100
99  Tab_jump    Edging 0.1375449820       0.11019452

CENTRALITY INVARIANCE TEST p-value
          expectedInfluence
BPS_1          0.8268692523
BPS_2          0.0027988804
BPS_3          0.0775689724
BPS_4          0.3410635746
BPS_5          0.0003998401
Tol_quant      0.0011995202
Tol_qual       0.7197121152
Binge          0.2083163733
Tab_jump       0.3142742903
Edging         0.4094362255
```

Model 2: BPS modelled as composite score

Sample 1

```
NETWORK INVARIANCE TEST
Test statistic M:
0.1016218
p-value 0.7357057

GLOBAL STRENGTH INVARIANCE TEST
Global strength per group:  2.162076 2.114356
Test statistic S:  0.0477204
p-value 0.4702119

EDGE INVARIANCE TEST
        Var1      Var2   p-value Test statistic E
7        BPS Tol_quant 0.2794882       0.05714705
13       BPS  Tol_qual 0.1147541       0.10162177
14 Tol_quant  Tol_qual 0.9092363       0.00554315
19       BPS     Binge 0.4774090       0.04450602
20 Tol_quant     Binge 0.1415434       0.08701369
21  Tol_qual     Binge 0.7445022       0.02367892
25       BPS  Tab_jump 0.2802879       0.05384779
26 Tol_quant  Tab_jump 0.1727309       0.08508083
27  Tol_qual  Tab_jump 0.8932427       0.00972496
28     Binge  Tab_jump 0.7668932       0.02093965
31       BPS    Edging 0.5801679       0.03240138
32 Tol_quant    Edging 0.7193123       0.02010784
33  Tol_qual    Edging 0.5649740       0.03553451
34     Binge    Edging 0.3458617       0.06568540
35  Tab_jump    Edging 0.4314274       0.05119741

CENTRALITY INVARIANCE TEST p-value
          expectedInfluence
BPS              0.76969212
Tol_quant        0.00159936
Tol_qual         0.09956018
Binge            0.22071172
Tab_jump         0.90963615
Edging           0.19712115
```

Sample 2

```
NETWORK INVARIANCE TEST
Test statistic M:
0.1816188
p-value 0.1747301

GLOBAL STRENGTH INVARIANCE TEST
Global strength per group:  2.157676 2.255957
Test statistic S:  0.09828098
p-value 0.4718113

EDGE INVARIANCE TEST
        Var1      Var2    p-value Test statistic E
7        BPS Tol_quant 0.13714514       0.09165213
13       BPS  Tol_qual 0.87045182       0.01285007
14 Tol_quant  Tol_qual 0.15433826       0.08860521
19       BPS     Binge 0.97840864       0.00206470
20 Tol_quant     Binge 0.02119152       0.17192864
21  Tol_qual     Binge 0.04038385       0.18161882
25       BPS  Tab_jump 0.44822071       0.01553720
26 Tol_quant  Tab_jump 0.13554578       0.10409935
27  Tol_qual  Tab_jump 0.80487805       0.02018198
28     Binge  Tab_jump 0.21871251       0.10818213
31       BPS    Edging 0.76529388       0.02082890
32 Tol_quant    Edging 0.88204718       0.01097968
33  Tol_qual    Edging 0.26749300       0.07881330
34     Binge    Edging 0.51979208       0.05764178
35  Tab_jump    Edging 0.21911236       0.09213745

CENTRALITY INVARIANCE TEST p-value
          expectedInfluence
BPS             0.393842463
Tol_quant       0.006797281
Tol_qual        0.843262695
Binge           0.111555378
Tab_jump        0.178728509
Edging          0.404238305
```

Node centrality

Model 1: BPS items modelled as individual nodes

Below cutoff

Sample 1 Sample 2

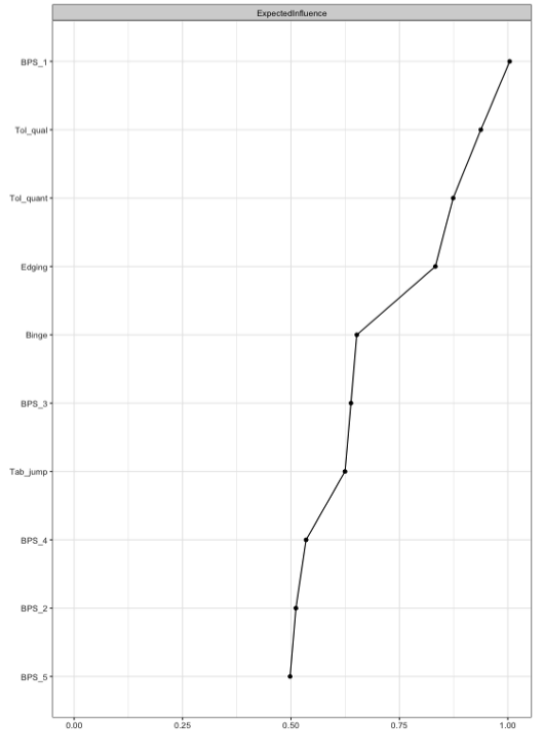

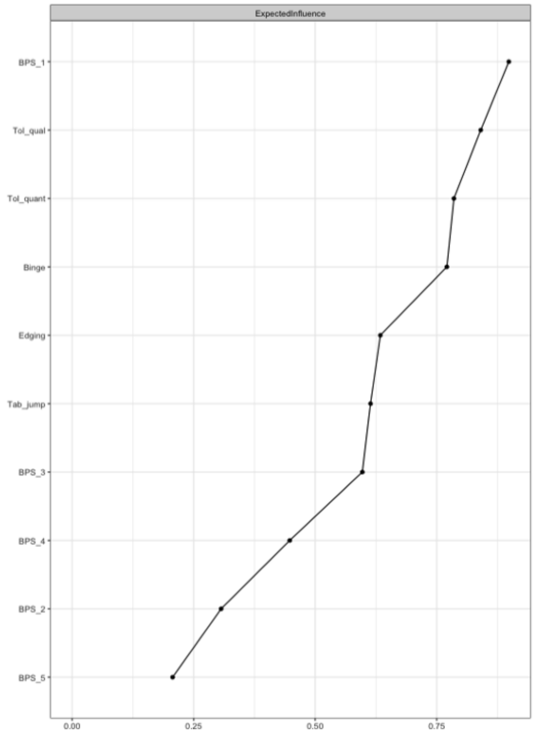

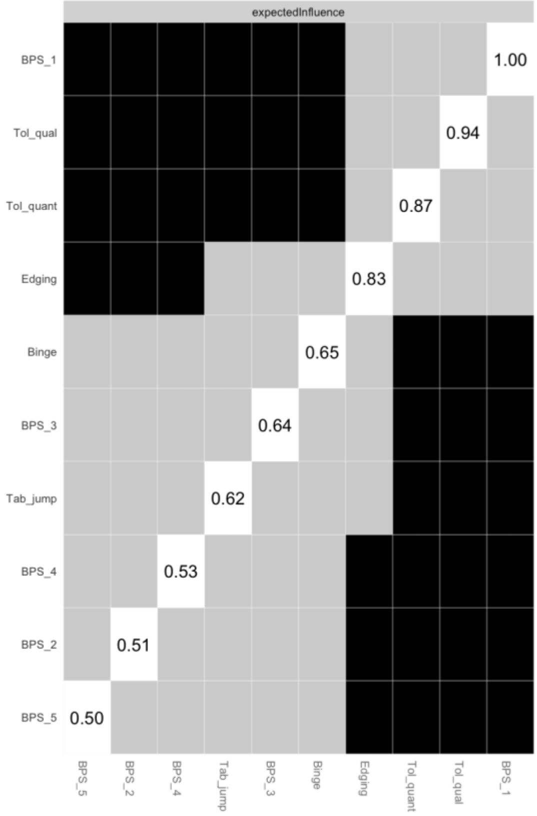

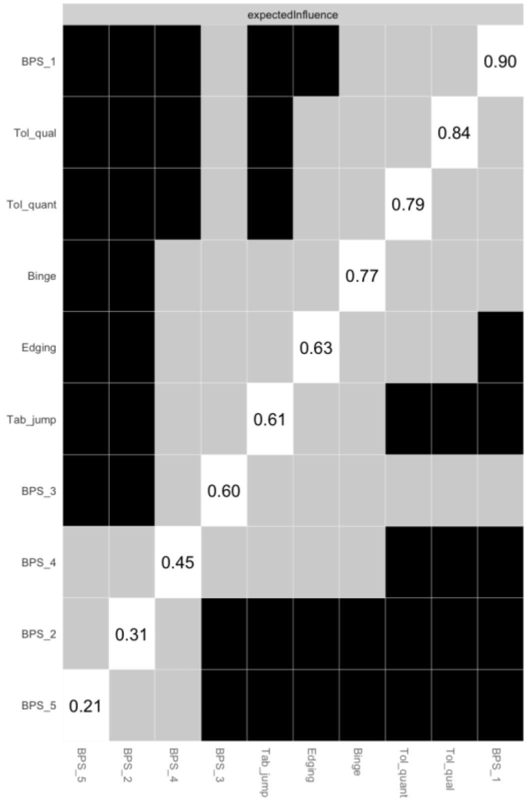

Above cutoff

Sample 1 Sample 2

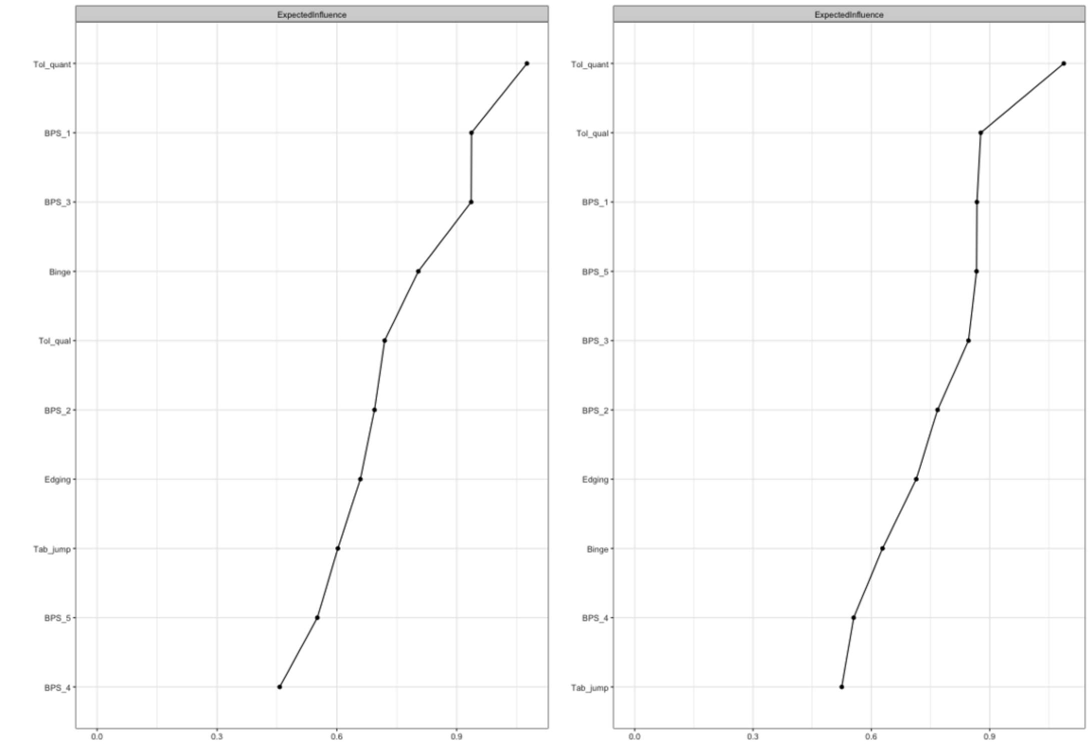

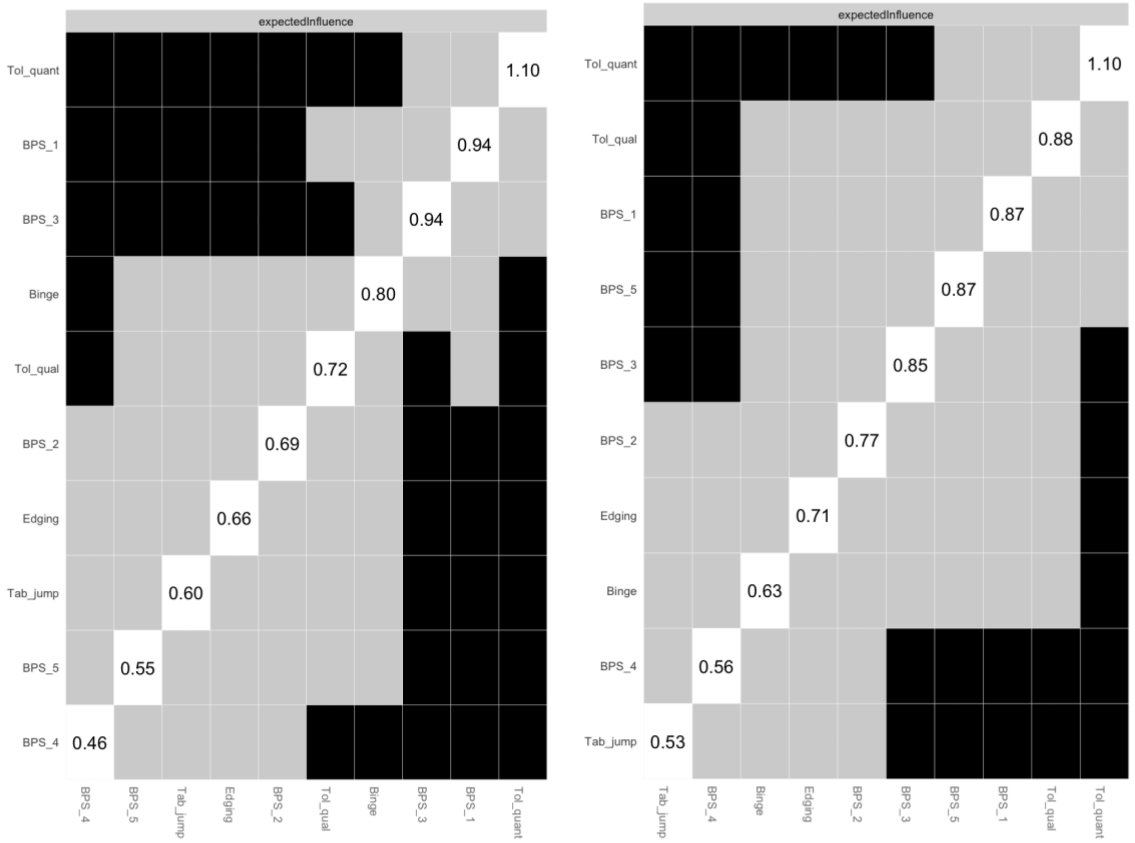

Model 2: BPS modelled as composite score

Below cutoff

Sample 1 | Sample 2

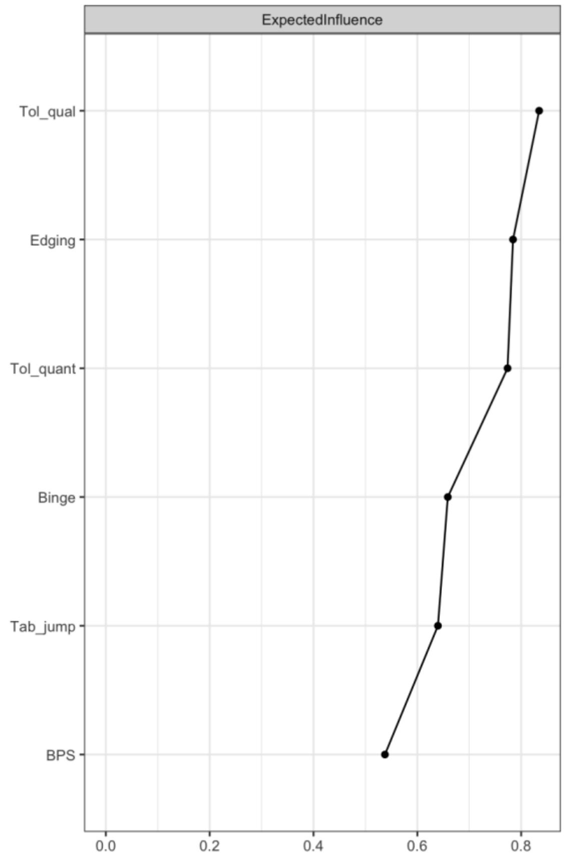

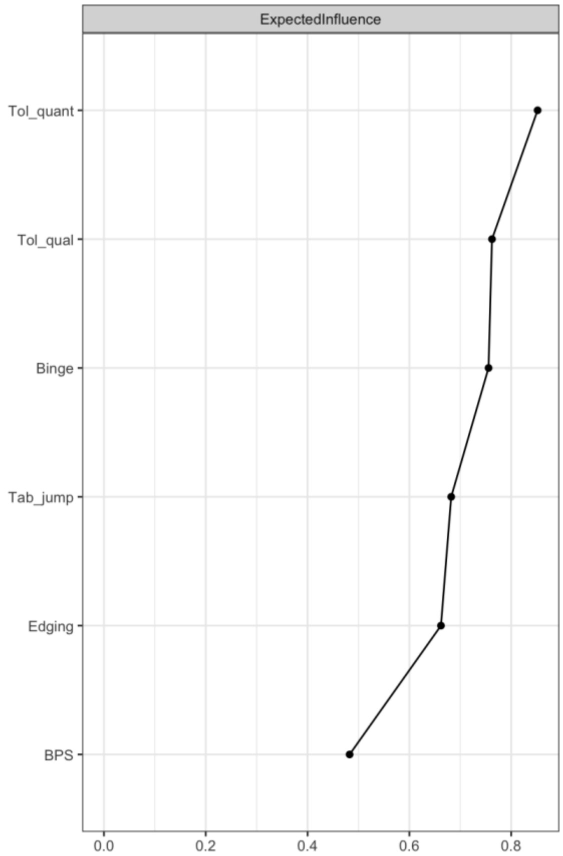

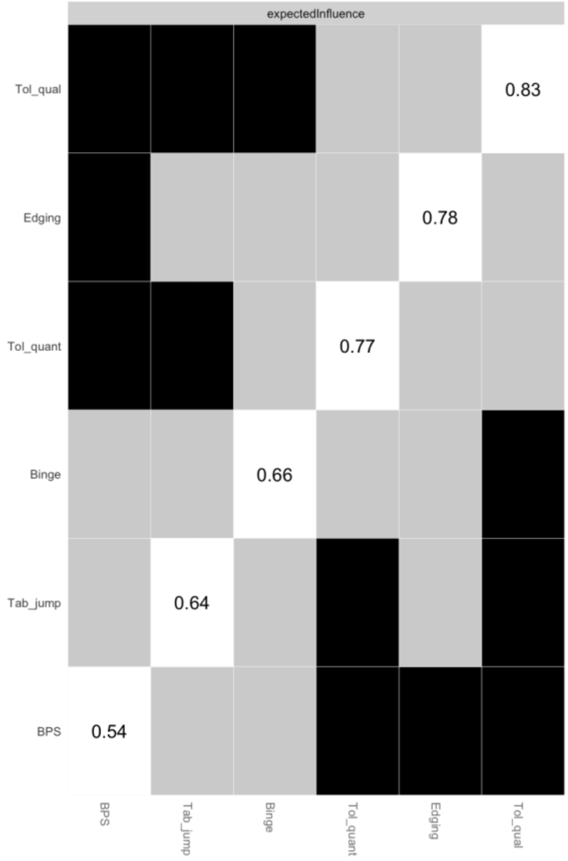

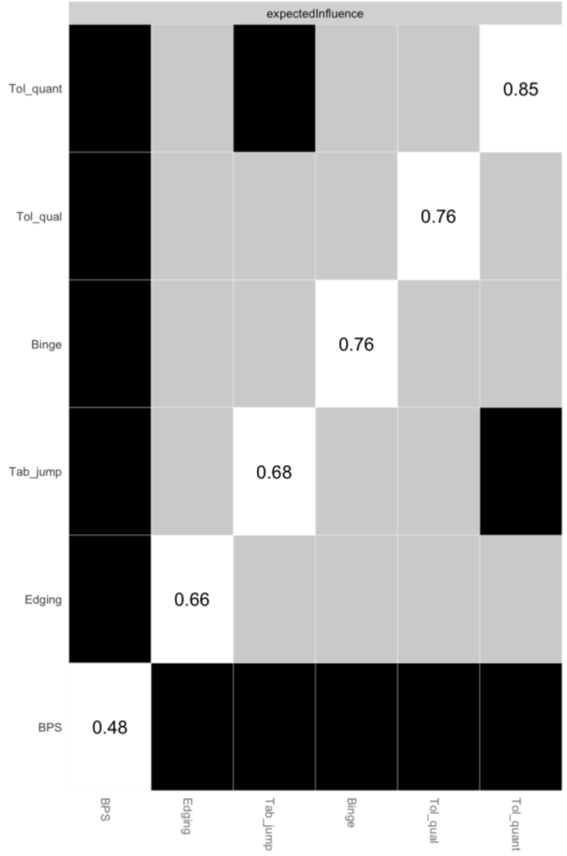

Above cutoff

Sample 1 Sample 2

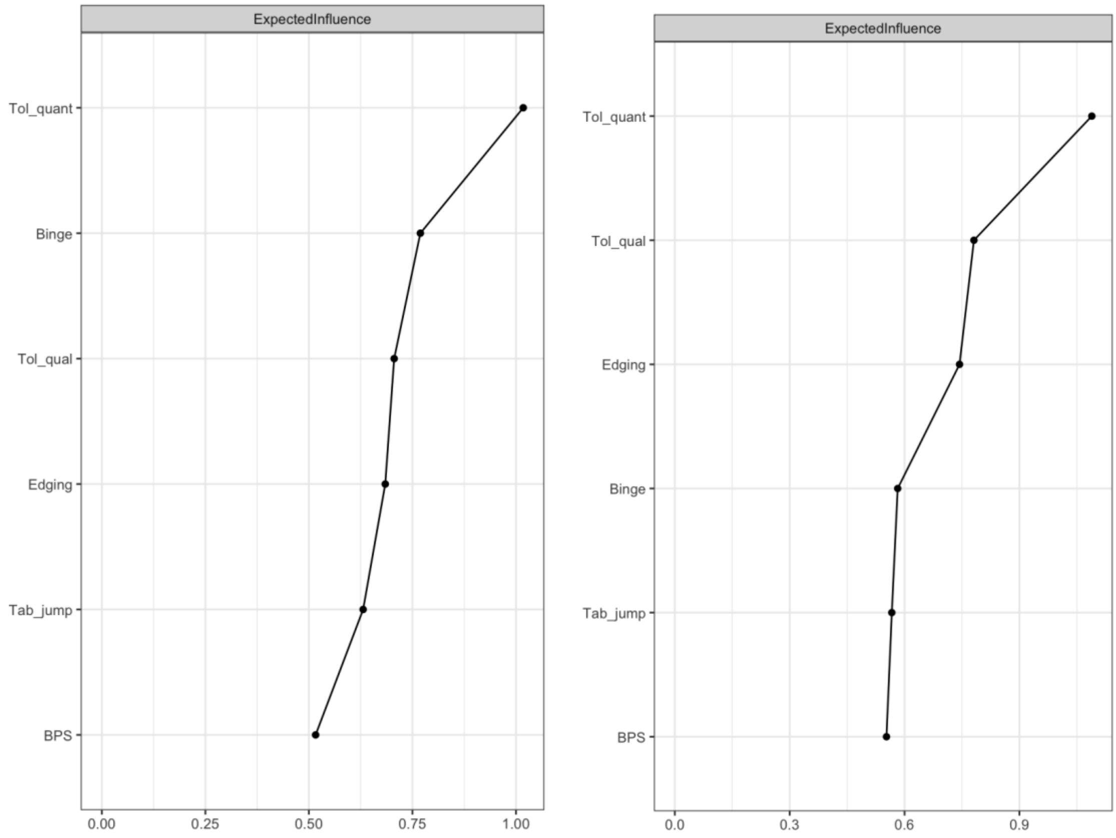

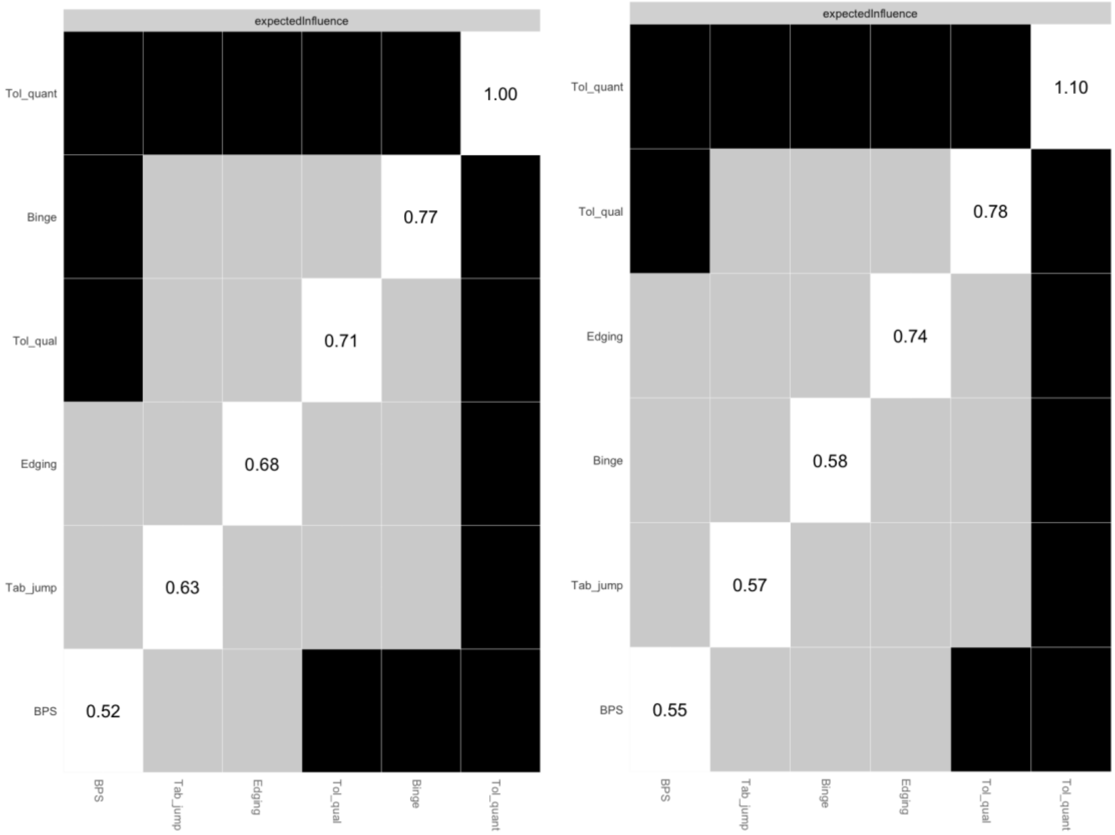

Bridge centrality

Model 1: BPS items modelled as individual nodes

Below cutoff

Sample 1 Sample 2

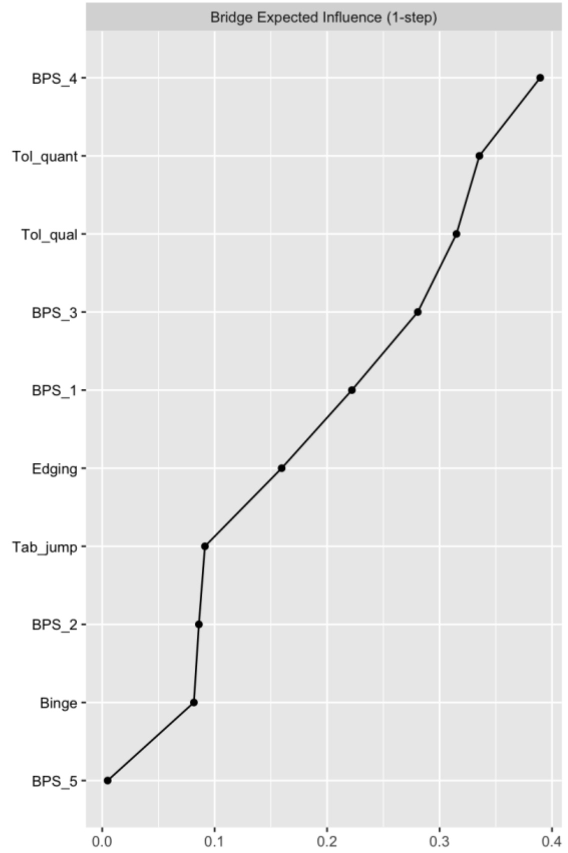

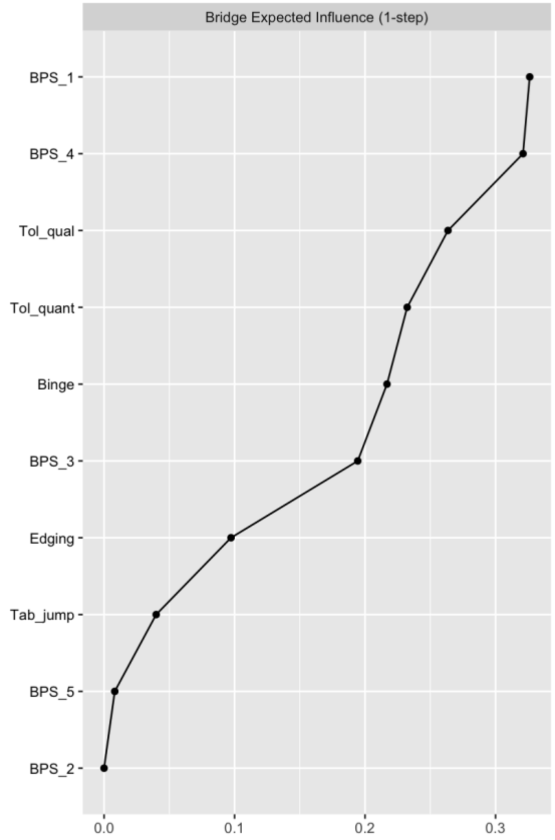

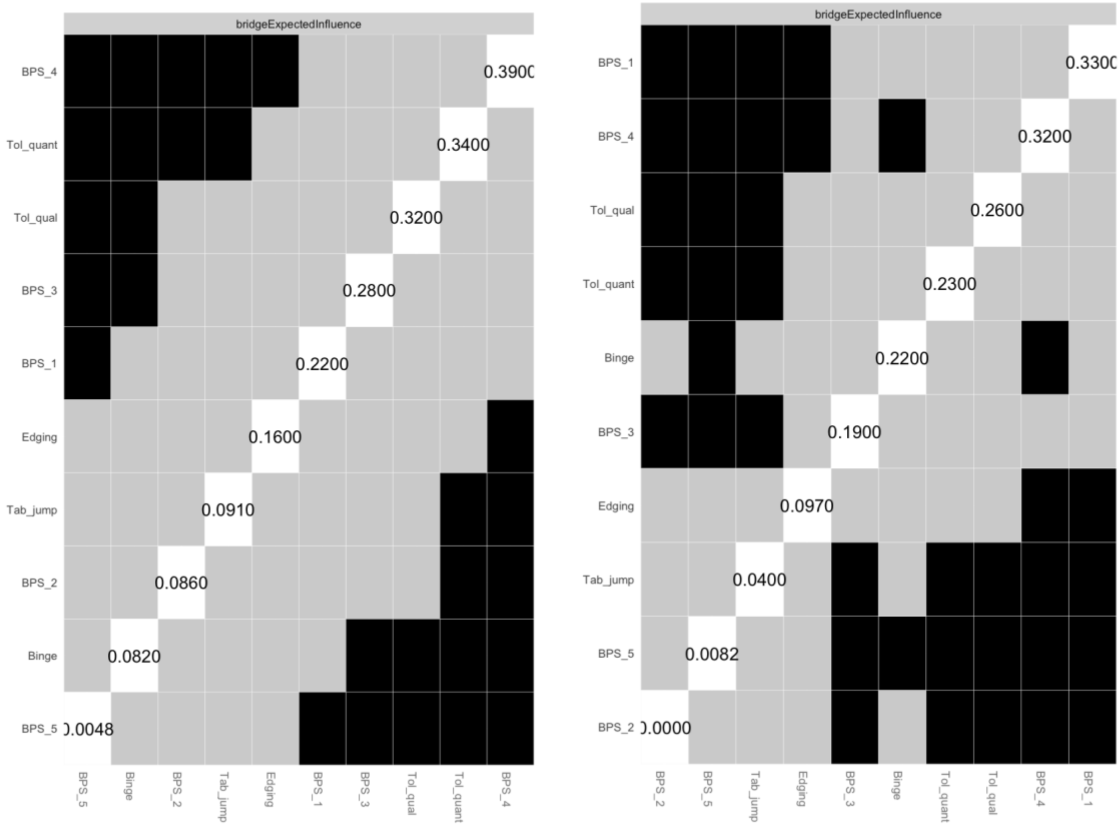
bridgeExpectedInfluence
BPS_4
Tol_quant
Tol_qual
BPS_3
BPS_1
Edging
Tab_jump
BPS_2
Binge
BPS_5
0.3900
0.3400
0.3200
0.2800
0.2200
0.1600
0.0910
0.0860
0.0820
0.0048
BPS_5
Binge
BPS_2
Tab_jump
Edging
BPS_1
BPS_3
Tol_qual
Tol_quant
BPS_4
bridgeExpectedInfluence
BPS_1
BPS_4
Tol_qual
Tol_quant
Binge
BPS_3
Edging
Tab_jump
BPS_5
BPS_2
0.3300
0.3200
0.2600
0.2300
0.2200
0.1900
0.0970
0.0400
0.0082
0.0000
BPS_2
BPS_5
Tab_jump
Edging
BPS_3
Binge
Tol_quant
Tol_qual
BPS_4
BPS_1

Above cutoff

Sample 1 Sample 2

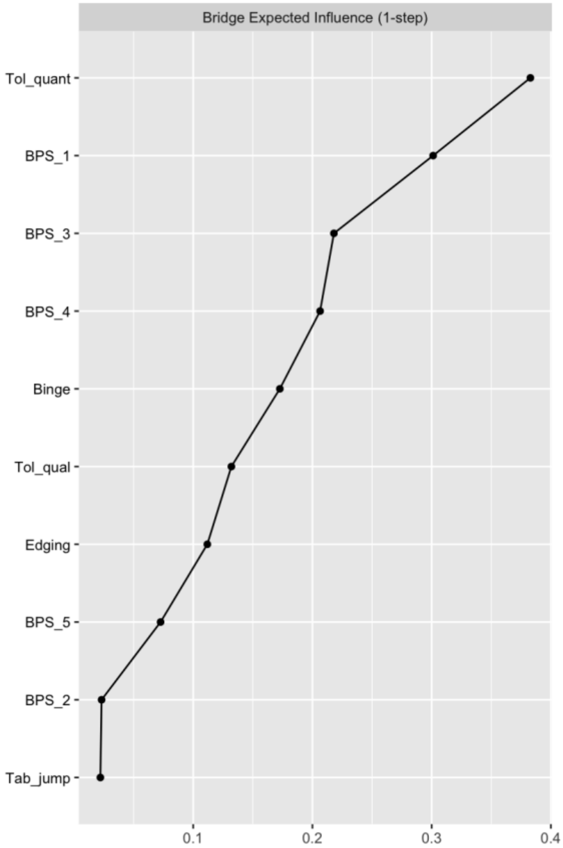

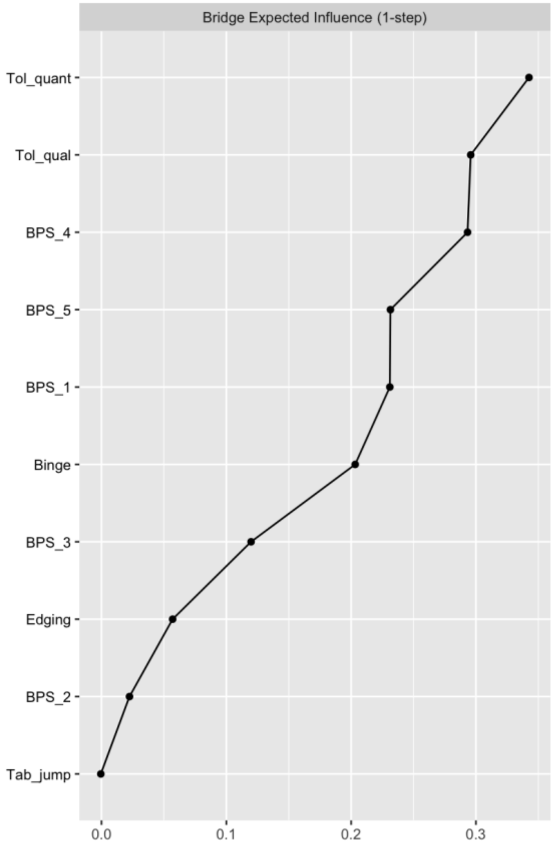

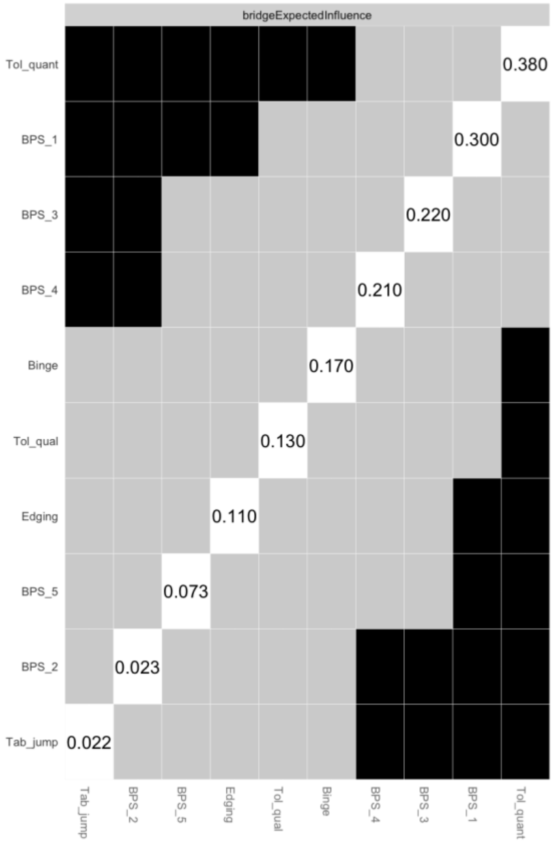

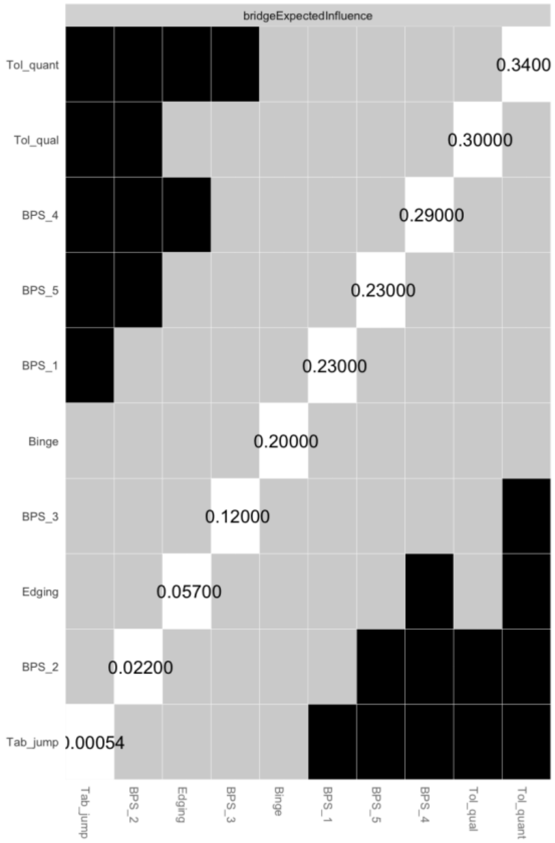

Network stability: correlation stability coefficients

Model 1: BPS items modelled as individual nodes

Above cutoff

Expected influence: Correlation stability coefficient (expected influence): Sample 1= 0.67, Sample 2= 0.52

Sample 1

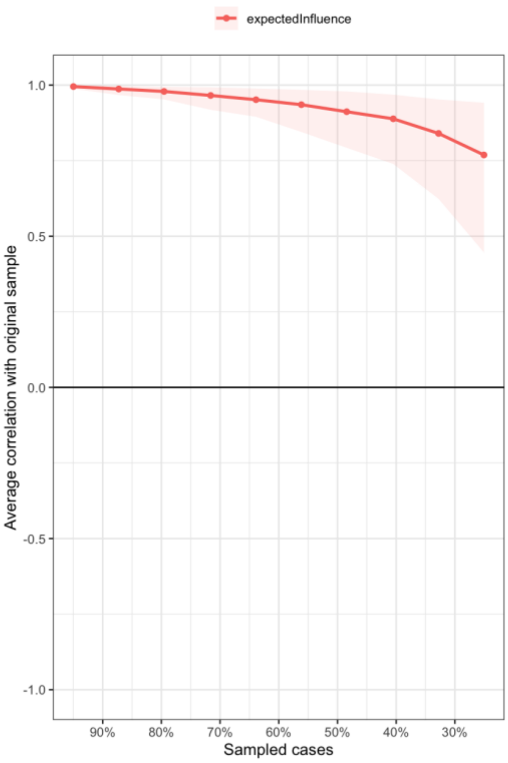

Sample 2

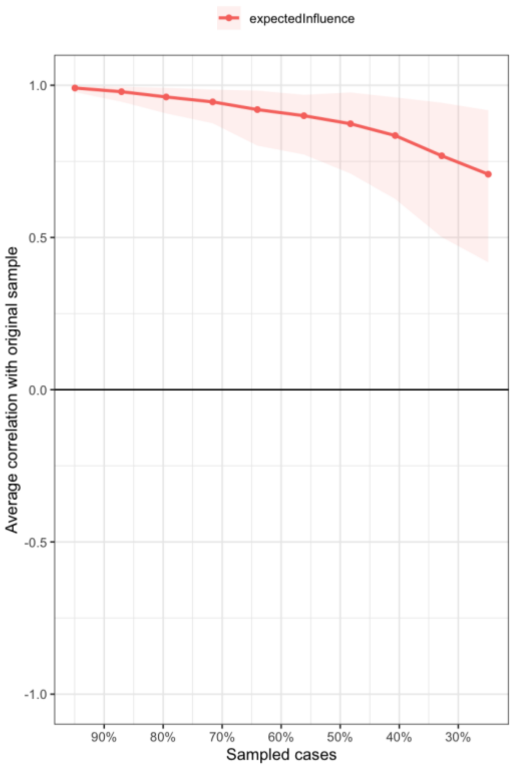

Bridge expected influence: Correlation stability coefficient (bridge expected influence): Sample 1= 0.52, Sample 2= 0.44

Sample 1 Sample 2

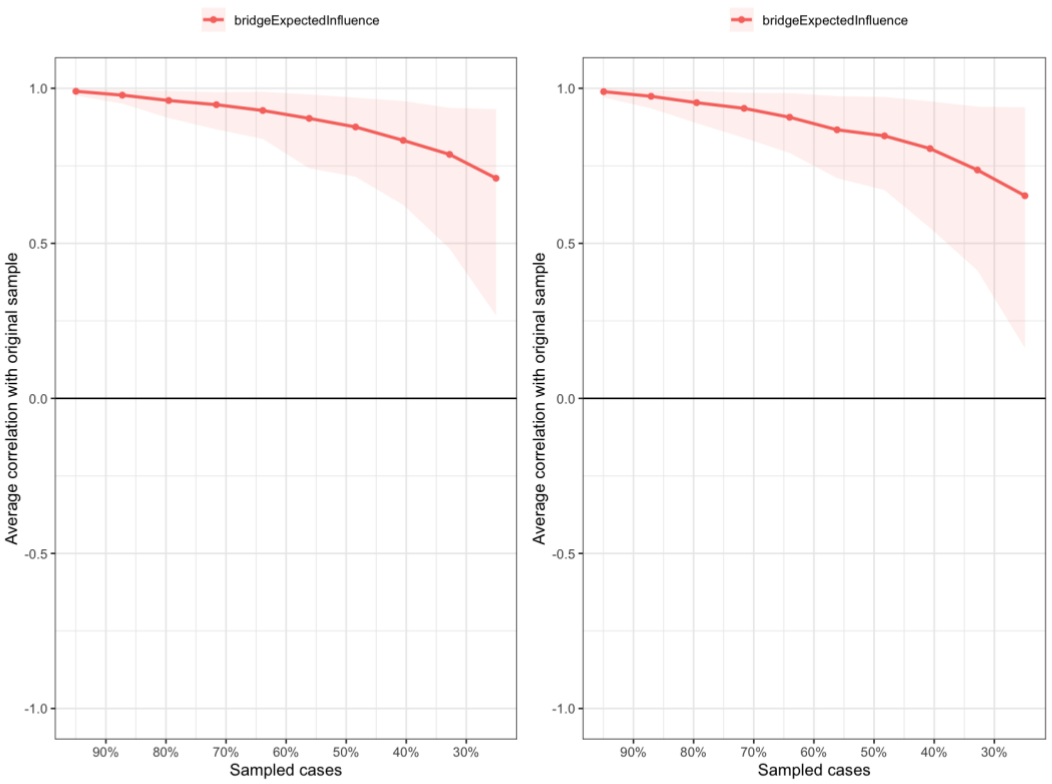

Below cutoff

Expected influence: Correlation stability coefficient (expected influence): Sample 1= 0.52, Sample 2= 0.60

Sample 1 Sample 2

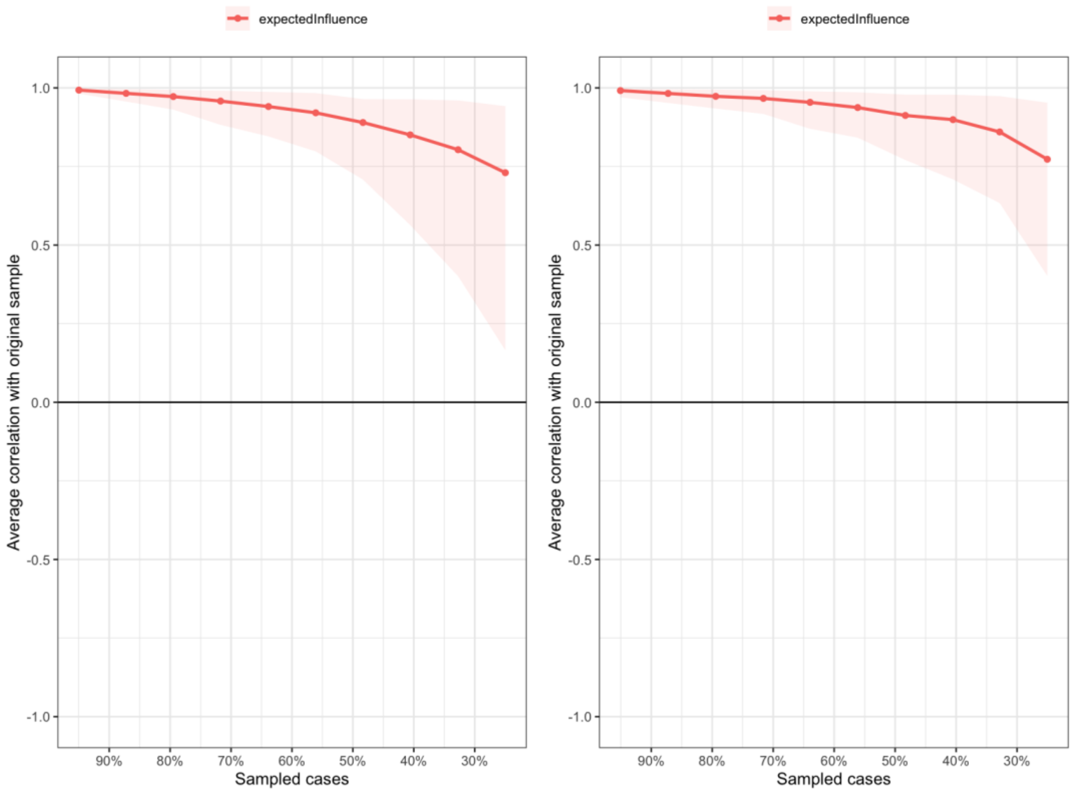

Bridge expected influence: Correlation stability coefficient (bridge expected influence): Sample 1= 0.52, Sample 2= 0.52

Sample 1

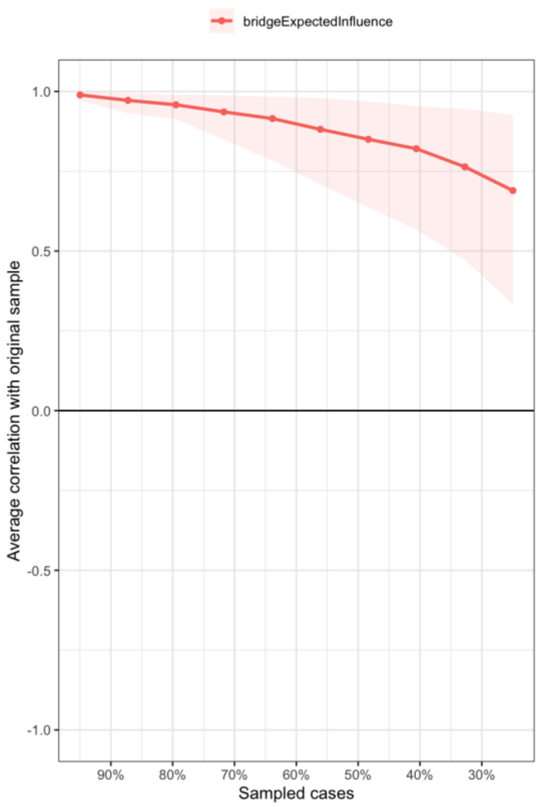

Sample 2

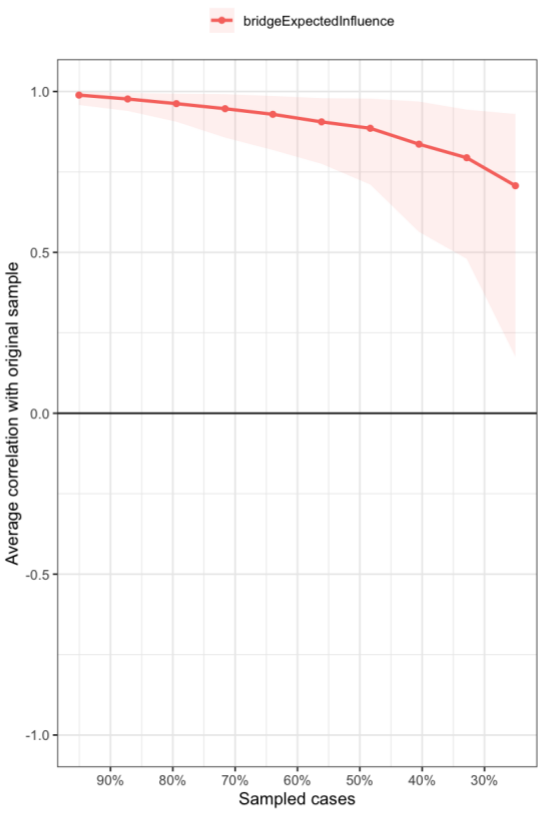

Model 2: BPS modelled as composite score

<u>Above cutoff</u>

Expected influence: Correlation stability coefficient (expected influence): Sample 1= 0.60, Sample 2= 0. 44.

Sample 1 Sample 2

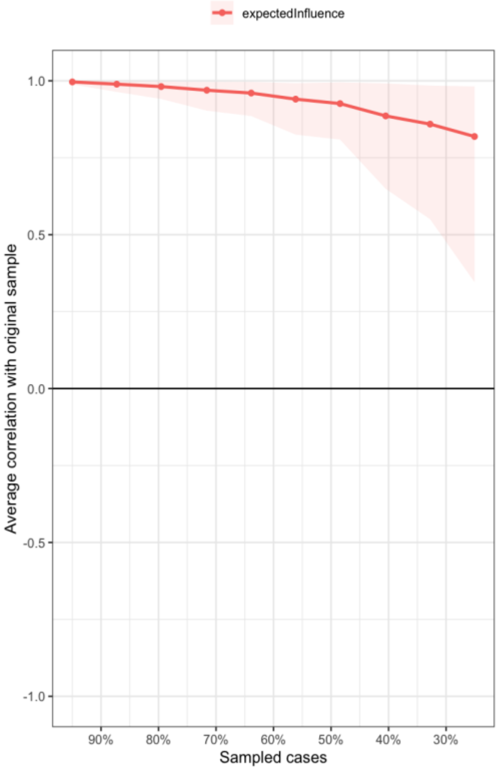

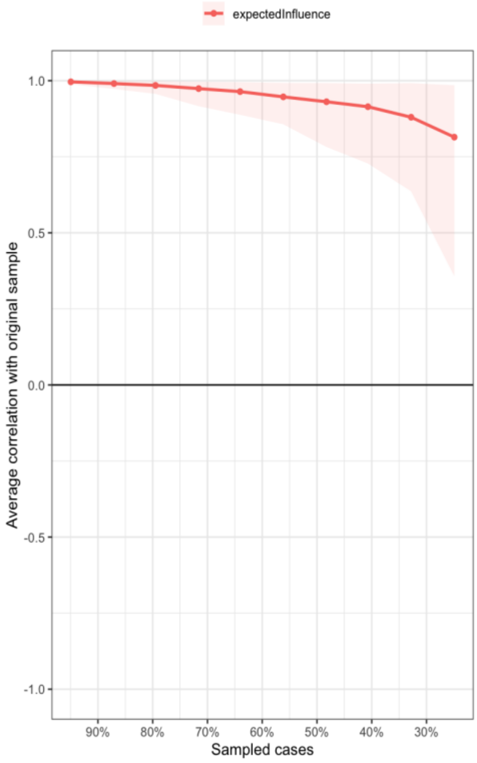

Below cutoff

Expected influence: Correlation stability coefficient (expected influence): Sample 1= 0.52, Sample 2= 0. 60.

Sample 1 Sample 2

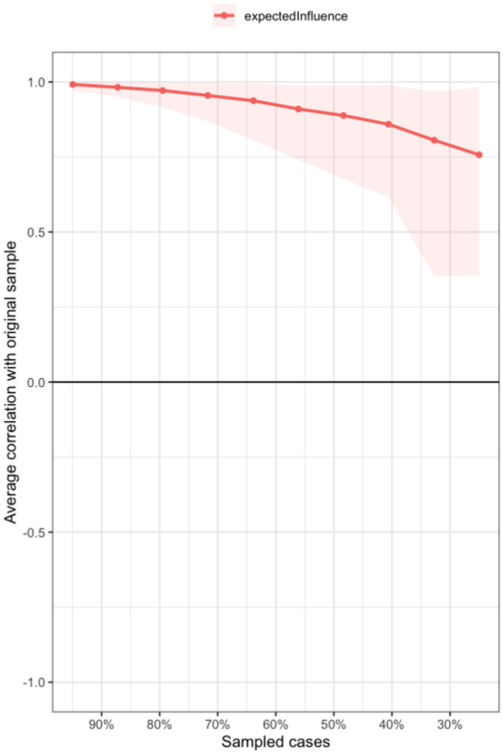

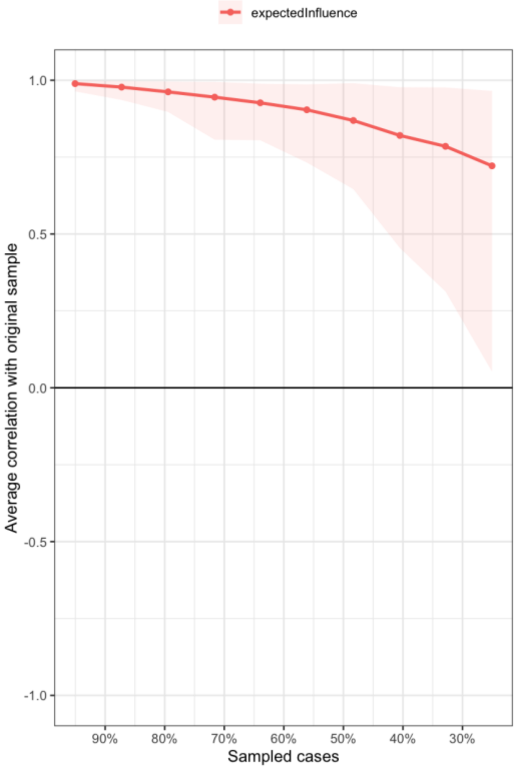

Supplement: Supplementary Materials [file EMS196188-supplement-Supplementary_Materials.pdf]
